# An illustrated key to the cuckoo wasps (Hymenoptera, Chrysididae) of the Nordic and Baltic countries, with description of a new species

**DOI:** 10.3897/zookeys.548.6164

**Published:** 2015-12-23

**Authors:** Juho Paukkunen, Alexander Berg, Villu Soon, Frode Ødegaard, Paolo Rosa

**Affiliations:** 1 Finnish Museum of Natural History, Zoology Unit, P.O. Box 17, FI-00014 University of Helsinki, Finland; 2 Kämnarsvägen 33F 1201, 226 46 Lund, Sweden; 3 Natural History Museum and Institute of Ecology and Earth Sciences, University of Tartu, Vanemuise 46, 51014 Tartu, Estonia; 4 Norwegian Institute for Nature Research – NINA, P.O. Box 5685 Sluppen, NO-7485 Trondheim, Norway; 5 Via Belvedere 8/d, I-20881 Bernareggio (MB), Italy

**Keywords:** Morphology, distribution, phenology, host species, Denmark, Estonia, Finland, Latvia, Lithuania, Norway, Sweden, *Chrysis borealis* sp. n.

## Abstract

The Chrysididae are a group of cleptoparasitic and parasitoid aculeate wasps with a large number of rare and endangered species. The taxonomy of this group has long been confusing due to the similarity of species and extensive intraspecific variation. We present for the first time a comprehensive dichotomous key for all 74 species found in the Nordic and Baltic countries. In addition to diagnostic characters, information on the distribution and biology of each species is also presented. A new species, *Chrysis borealis* Paukkunen, Ødegaard & Soon, **sp. n.** is described on the basis of specimens collected from Fennoscandia. *Chrysis gracillima* Förster, 1853 is recorded as new to the Nordic and Baltic countries.

## Introduction

Chrysidid wasps, also known as cuckoo wasps, represent one of the largest families of aculeate Hymenoptera within the superfamily Chrysidoidea. More than 2.500 species are known worldwide (Aguiar et al. 2013) and approximately 490 of these have been recorded from Europe (Mitroiu et al. 2015). The species richness decreases towards the north of Europe, and a total of 74 species have been found in Fennoscandia, Denmark and the Baltic countries (Paukkunen et al. 2014). Cuckoo wasps, excluding Amiseginae and Loboscelidiinae, which are not present in Europe, are well known for their bright metallic colours and cleptoparasitic or parasitoid lifestyle. In northern Europe, species of the subfamily Chrysidinae parasitise solitary wasps (Vespidae and Crabronidae) and solitary bees (Megachilidae), whereas species of Cleptinae attack tenthredinid and diprionid sawflies. Despite their attractive appearance, chrysidids have a reputation for being a taxonomically difficult group, and the biology of several species is still poorly known.

A detailed history of cuckoo wasp research in the Nordic and Baltic countries was presented recently by Paukkunen et al. (2014). More than 200 publications including information on chrysidids have been published from the region, but most of them consist of only scattered records in poorly circulated journals or reports. Only a few authors have conducted more extensive faunistic studies at a national or wider scale. Faunistic surveys, including identification keys, have been compiled by Dahlbom (1829, 1831), Thomson (1862, 1870) and Aurivillius (1911) in Sweden, by Borries (1891) in Denmark, and by Sahlberg (1910) and Hellén (1920) in Finland. The monographs of Dahlbom (1845, 1854) also include keys and descriptions of several Nordic species. A simple key to the Swedish genera was later presented by Landin (1971). The faunistic studies from Estonia (Soon 2004), Latvia (Tumšs and Maršakovs 1970) and Lithuania (Orlovskytė et al. 2010) did not include identification keys of chrysidids. In recent decades, species determination of chrysidids in the Nordic and Baltic countries has mainly relied on the works of Linsenmaier (1951, 1959, 1997), Morgan (1984), Kunz (1994) and van der Smissen (2010), which focus primarily on the central European fauna, but include most of the North European species.

Scattered notes on the biology of European cuckoo wasps have been published by several authors in numerous articles and reports, and these data have been compiled by e.g. Kunz (1994) and Rosa (2006). An important contribution to the knowledge of hosts of North European species was recently made by Pärn et al. (2014) in Estonia. The Nordic open access databases of entomological observations, *Artportalen* (http://www.artportalen.se) in Sweden, *Artskart* (http://artskart.artsdatabanken.no) in Norway and *Hyönteistietokanta* (http://hyonteiset.luomus.fi) in Finland provide extensive sources of information on the phenology and habitats of cuckoo wasps. Many unpublished records of hosts, habitats and phenology can also be found in public and private cuckoo wasp collections.

As most publications with information on the identification and biology of the Nordic and Baltic cuckoo wasps are scattered, outdated and/or difficult to find or use, there is a need for a new comprehensive key for the North European species including biological information. Cuckoo wasps include an exceptionally large number of red-listed and endangered species in the Nordic countries, which also highlights the importance of their reliable identification (Cederberg et al. 2010, Hansen et al. 2010, Paukkunen 2010).

The aim of this study is to present a simple dichotomous identification key for the Nordic and Baltic species, and to compile all relevant and reliable information on their distribution, abundance and biology, including phenology and host species from publications and collections. The key will hopefully arouse more interest in chrysidids among entomologists, and provide a basis for further, more detailed studies on the distribution, biology and morphology of North European species.

## Material and methods

**Figure 1.**
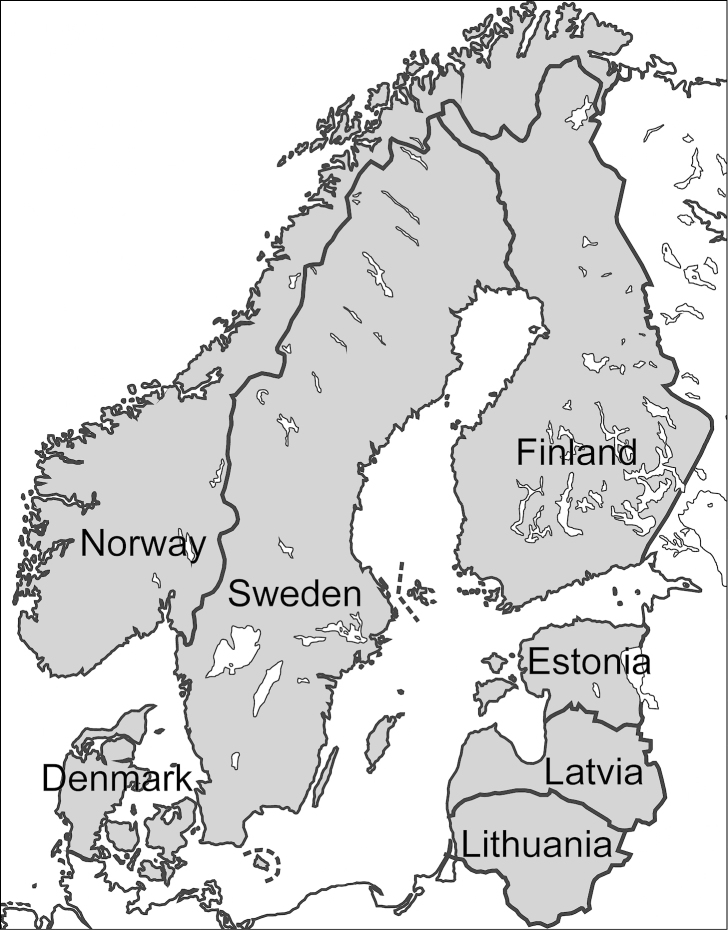
Map of the study area.

The geographic area covered by the study includes the Nordic and Baltic countries, which are located in northern Europe (Fig. 1). The nomenclature and arrangement of the taxa follows Paukkunen et al. (2014) and Rosa et al. (2015), and the morphological terminology is based on Morgan (1984) and Kimsey and Bohart (1991), with a few exceptions. Most notably, mesosoma is used instead of thorax, metasoma instead of abdomen or gaster, mesoscutum instead of scutum and mesoscutellum instead of scutellum. These exceptions are made in order to harmonise modern general Hymenoptera terminology (Hymenoptera Anatomy Consortium 2015). The following abbreviations are used: T = tergite, S = sternite and F = flagellomere (= flagellar segment, pseudosegment of flagellum). Numbers are used for antennal and metasomal segments, for example, F2 refers to the second flagellomere. The key is mainly based on the works of Linsenmaier (1997) and van der Smissen (2010). In order to keep the key simple and concise, only easily visible and relatively constant characters have been selected. Several new diagnostic characters have also been found and included in the key. In addition to figures of morphological details, one dorsal habitus picture of an entire specimen is presented for each genus.

The species treatments consist of the following information: name, synonymy, diagnosis, distribution and biology. Only the more common synonyms and erroneously interpreted names, which have been used in connection with cited records from the study area, are presented below the valid name of the taxon. If the currently used name differs from the original combination, it is added to the synonymic list with a citation of the study, in which the rearrangement was made. The abundance of each species is estimated using the scale 1) very common (more than 5000 records), 2) common (ca 1000–5000 records), 3) relatively common (ca 500–1000 records), 4) relatively rare (ca 200–500 records), 5) rare (ca 10–200 records), 6) very rare (less than 10 records). This estimation is mainly based on collected material and therefore it essentially shows how commonly a species is collected, but might not accurately indicate its actual abundance in nature. A summary of the distribution of chrysidid species in the Nordic and Baltic countries is presented in Table 3.

The biology section includes information on the habitat, flight season and host species. The presented information on the distribution, abundance and biology has been compiled from published literature, entomological databases and several public and private collections, as well as our own observations. Host species, plants and habitats that are not found in the Nordic and Baltic countries are usually not mentioned. The most important studied collections are listed below:

**LMSZ** Museum of Zoology, University of Latvia; Riga, Latvia

**MZH** Finnish Museum of Natural History, University of Helsinki; Helsinki, Finland

**MZLU** Zoological Museum, Lund University; Lund, Sweden

**NHRS** Swedish Museum of Natural History; Stockholm, Sweden

**NMLS** Natur-Museum Luzern; Luzern, Switzerland

**NUM** NTNU University Museum, Trondheim, Norway

**TUZ** Natural History Museum, University of Tartu; Tartu, Estonia

**ZMAA** Zoological Museum, Åbo Akademi University; Turku, Finland

**ZMUC** Zoological Museum, University of Copenhagen; Copenhagen, Denmark

**ZMUO** Zoological Museum, University of Oulu; Oulu, Finland

**ZMUT** Zoological Museum, University of Turku; Turku, Finland

Lists of examined material have not been included in the species treatments due to the large number of studied specimens. Accurate data is given only if a species is recorded for the first time from a country. Some information about the examined material has been published earlier by Paukkunen et al. (2014), and most of the Finnish data is openly accessible through the Finnish Biodiversity Info Facility (http://laji.fi). Data on DNA barcoded specimens are available at the Barcode of Life Data System (http://www.boldsystems.org, Ratnasingham and Hebert 2007).

Morphological measurements were prepared using an ocular micrometer on a Wild M5 and a Leica MZ75 stereomicroscope. All pictures were prepared by Alexander Berg, if not otherwise specified. The photos were taken with a Canon6D camera, using a Schneider-Kreuznach Componon-S 50 mm f2.8 and Schneider-Kreuznach Componon 28 mm f1.4 enlarger lenses extended on Pentacon M42 bellows. A Proxxon KT-70 microstage was used for photo stepping and Zerene Stacker v1.04 for stacking the photos.

In order to use the key successfully, specimens should be properly mounted or pinned with both the dorsal and ventral surfaces of the metasoma visible. In the *Chrysis ignita* and *Chrysis fasciata* species-groups, the mandibles of both sexes should be opened, genital capsules of males should be extracted and ovipositors of females everted. Colouration of specimens collected with traps containing liquid preservatives, softened using hot water or having been kept in sunlight for a long time, can deviate from the original colouration. Additionally, the colour of fresh and liquid preserved specimens can change when they are dried, most notably greenish shades turn bluish in dry specimens. Geographical variation in colouration is also observed in many species, whereby northern specimens tend to be darker than southern ones.

Distinguishing the sexes of chrysidids can be difficult if the telescope-like ovipositor of the female is not exserted, or the genital capsule of the male has not been extracted. In males, the third metasomal sternite is completely flat and the semitransparent membranous posterior margin of the fourth sternite is usually visible. In females, the third sternite is generally thicker posteriorly and the posterior margin of the fourth sternite is opaque. Additionally, a slender needle-like structure (formed by the first valvulae) can be seen on the tip of the ovipositor in females. This structure is visible even if the ovipositor is not fully exerted.

**Figures 2–10.**
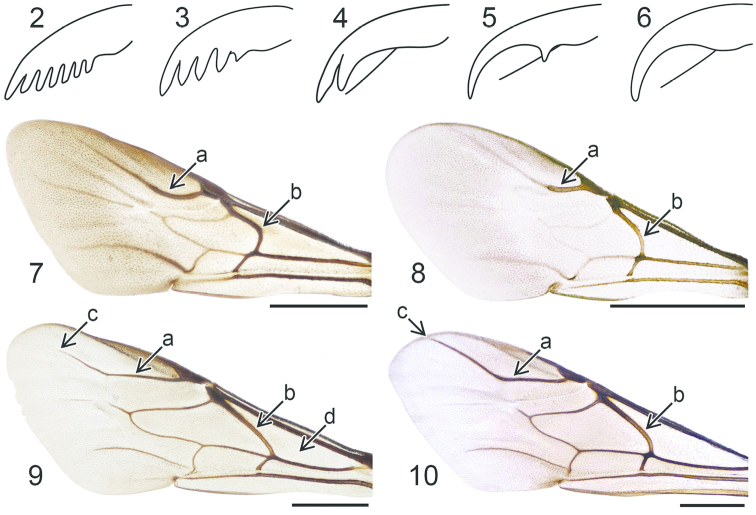
Tarsal claw: **2** *Pseudomalus triangulifer* **3** *Holopyga generosa* **4** *Hedychrum nobile* **5** *Hedychridium roseum* **6** *Chrysis longula*. Forewing: **7** *Holopyga generosa* **8** *Hedychridium ardens* **9** *Pseudospinolia neglecta* **10** *Chrysis impressa*, a and c radial sector vein, b medial vein, d medial cell.

## Systematics

### Key to chrysidid genera of the Nordic and Baltic countries

**Table:** 

1	Metasoma with four (female) or five (male) external tergites, ventral surface convex, colour anteriorly non-metallic red, posteriorly black, often with blue-green metallic reflections (Figs 11, 16, 17). Pronotum campanulate (Figs 11–13)	***Cleptes* Latreille (Cleptinae)**
–	Metasoma with three (or four in *Parnopes* male) external tergites, ventral surface flat or concave, colour variable, often completely metallic, if non-metallic red then never posteriorly extensively black. Pronotum not campanulate. (Chrysidinae)	**2**
2	Metasoma with three (female) or four (male) external tergites (Fig. 209). T1 mostly metallic and subsequent tergites usually non-metallic red (Fig. 209). Posterior margin of metasoma with numerous small irregular teeth. Mouthparts (galea and glossa) strongly developed, longer than rest of head. Tegula large and broad, covering base of forewing and hindwing (Fig. 209)	***Parnopes grandior* (Pallas) (Parnopini)**
–	Metasoma with three external tergites in both sexes. All tergites usually with metallic colour, and if non-metallic, then T1 also without metallic reflections. Posterior margin of metasoma without small irregular teeth. Mouthparts short. Tegula small, covering only base of forewing	**3**
3	Tarsal claw with one or more subapical teeth (Figs 2–5). Radial sector vein of forewing basally curved (Figs 7a, 8a). Head without preoccipital carina. T3 without subapical pit row and apical teeth. (Note: *Elampus bidens* (Förster), which might be found in North Europe in the future, has two apical teeth on T3, but also a tongue-like metascutellar projection, as in Fig. 38.) (Elampini)	**4**
–	Tarsal claw simple, without subapical teeth (Fig. 6). Radial sector vein of forewing basally straight (Figs 9a, 10a). Head with preoccipital carina, ending in hook (Figs 138–149). T3 with subapical pit row, with or without apical teeth. (Chrysidini)	**10**
4	Tarsal claw with single subapical tooth (Figs 4, 5). Temple rounded in dorsal view (Fig. 74). Medial cell of forewing with short setae	**5**
–	Tarsal claw with more than one subapical tooth (Figs 2, 3). Temple angular in dorsal view (Fig. 48). Medial cell of forewing with or without setae	**6**
5	Subapical tooth of tarsal claw almost as large as apex, so that tip appears forked (Fig. 4). T3 usually laterally with two small angular projections (Figs 61–63)	***Hedychrum* Latreille**
–	Tarsal claw with very small submedial tooth, remote from apex (Fig. 5). T3 without angular projections	***Hedychridium* Abeille de Perrin**
6	Medial cell of forewing with setae. Medial vein of forewing strongly curved (Fig. 7b). T3 without apical notch	***Holopyga* Dahlbom**
–	Medial cell of forewing without setae. Medial vein of forewing only slightly curved (Fig. 8b). T3 usually with apical notch (Figs 21, 23, 25, 31, 32, 36, 44–46)	**7**
7	Metascutellum dorsally with large tongue-like projection (Fig. 38). Gena of female with row of dense short setae (Fig. 41)	***Elampus* Spinola**
–	Metascutellum without projection. Gena without row of short setae	**8**
8	Mesoscutum with large punctures concentrated postero-medially between notauli (Fig. 27). Ventral margin of mesopleuron strongly projecting (Fig. 28). Head and mesosoma usually with long pubescence, setae twice as long as diameter of mid-ocellus	***Pseudomalus* Ashmead**
–	Mesoscutum without punctures, with irregularly scattered punctures (Figs 20, 22, 24) or with punctures clumped along notauli (Fig. 35). Ventral margin of mesopleuron weakly projecting (Fig. 19). Head and mesosoma with short pubescence, setae not more than twice as long as diameter of mid-ocellus	**9**
9	Apical notch of T3 with thickened margin (Fig. 36). Lateral margin of T3 with concave depression prior to apical notch (Fig. 36). Mesoscutum with coarse punctation (Fig. 35). Metascutellum sharply elevated	***Philoctetes truncatus* (Dahlbom)**
–	Apical notch of T3 without thickened margin (Figs 21, 23, 25). Lateral margin of T3 rounded or relatively straight, without concave depression prior to apical notch (Figs 21, 23, 25). Mesoscutum without or with finer punctation (Figs 20, 22, 24). Metascutellum not sharply elevated	***Omalus* Panzer**
10	Radial sector vein of forewing ending before wing margin, at a distance approximately equal to the length of the pterostigma (Fig. 9c). Posterior margin of T3 without apical teeth	**11**
–	Radial sector vein of forewing extending to wing margin, or nearly so (Fig. 10c). Posterior margin of T3 with or without apical teeth	**12**
11.	Body entirely blue (Fig. 76). Medial cell of forewing without setae. Scapal basin with fine punctation	***Spinolia unicolor* (Dahlbom)**
–	Body bicoloured, head and mesosoma blue-green, metasoma red (Fig. 75). Medial cell of forewing with setae. Scapal basin medially with fine cross-ridging	***Pseudospinolia neglecta* (Shuckard)**
12	Posterior margin of T3 without apical teeth or angular prominences (Figs 206–208). Frons flat, without transverse frontal carina (Figs 201, 202). Male usually with F2 to F5 ventrally bulging (Fig. 204)	***Chrysura* Dahlbom**
–	Posterior margin of T3 with apical teeth or angular prominences (Figs 78–88, 198), or rarely bluntly triangular or rounded without teeth (*Chrysis gracillima*, *Chrysis succincta* and *Chrysis leachii*) (Fig. 77). Frons with deep scapal basin and transverse frontal carina (Figs 150–154)	**13**
13	Posterior margin of T3 with three apical teeth, lateral teeth may be angular projections (Fig. 198). Black spots of S2 small and joined together into one central spot of variable shape (Fig. 199). Body entirely blue-green, sometimes blackish (Fig. 197)	***Trichrysis cyanea* (Linnaeus)**
–	Posterior margin of T3 with different number of teeth (Figs 78–88), or rarely without teeth (Fig. 77). Black spots on S2 not as above. Body colour variable	***Chrysis* Linnaeus**

### I. Subfamily Cleptinae

**Figure 11.**
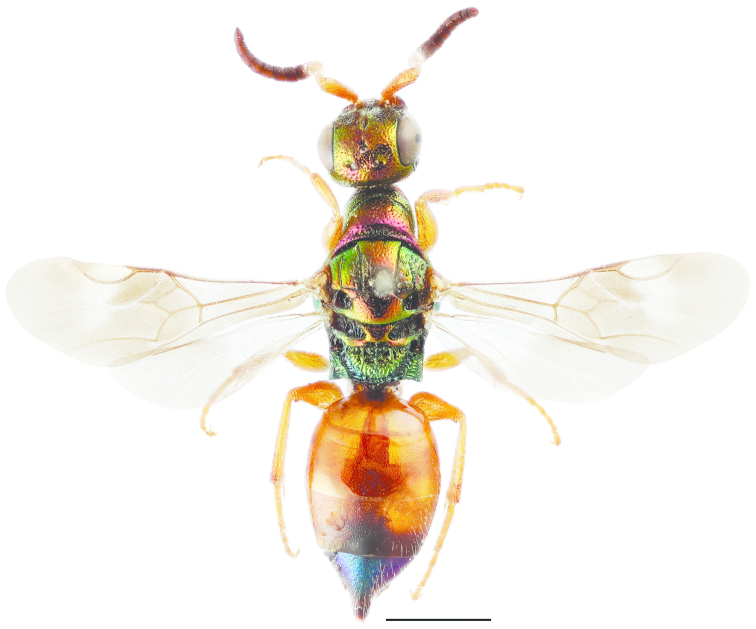
*Cleptes semiauratus* ♀. Scale 1 mm. (Photo: Pekka Malinen).

**Figures 12–17.**
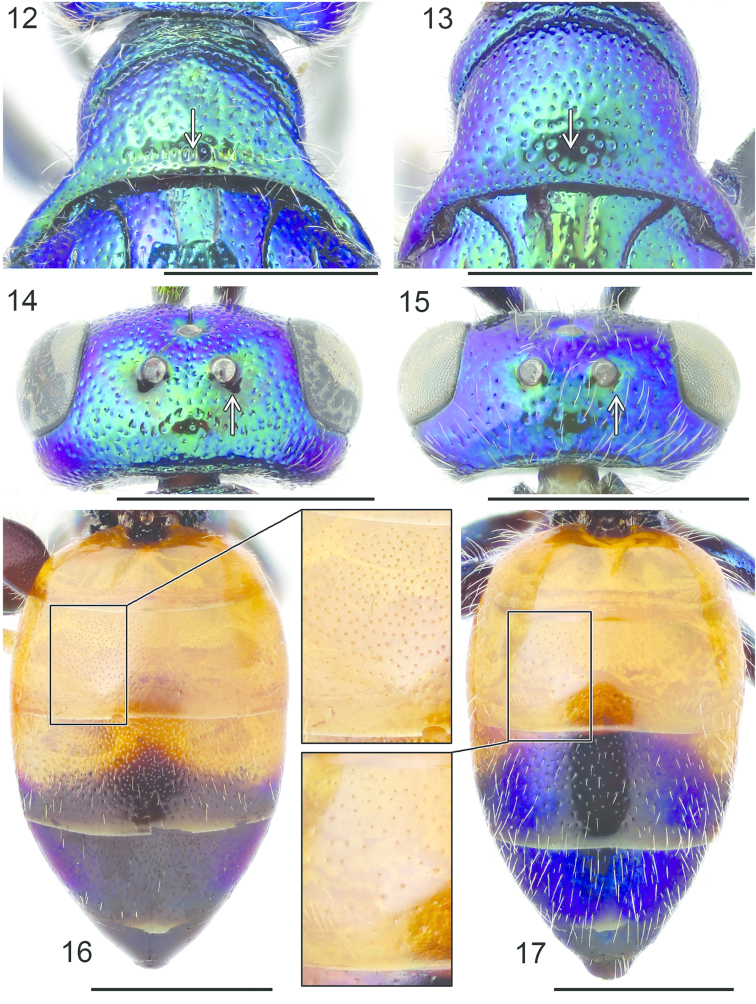
Pronotum, dorsal view: **12** *Cleptes semiauratus* ♂ **13** *Cleptes nitidulus* ♂. Head, dorsal view (arrow indicating postocellar fovea): **14** *Cleptes nitidulus* ♂ **15** *Cleptes semicyaneus* ♂. Metasoma, dorsal view: **16** *Cleptes nitidulus* ♂ **17** *Cleptes semicyaneus* ♂. Scale 1 mm.

This subfamily represents the most basally arising lineage of Chrysididae (Kimsey and Bohart 1991, Carpenter 1999). It is characterised by the following features: frons without scapal basin, pronotum narrowed submedially and campanulate in dorsal view (Figs 11–13), propodeum rectangular in profile, with horizontal dorsal surface, metasoma with four external segments in the female and five segments in the male (Figs 11, 16, 17), and metasomal venter convex. Cleptinae are parasitoids of sawfly prepupae (Hymenoptera: Symphyta) of the families Tenthredinidae and Diprionidae (Kimsey and Bohart 1991). The subfamily includes three genera, *Cleptes* Latreille, 1802, *Cleptidea* Mocsáry, 1904 and *Lustrinia* Kurian, 1955, of which only *Cleptes* is known from Europe (Kimsey and Bohart 1991, Móczár 1996). Currently, a total of 121 Cleptinae species are recognised worldwide (Wei et al. 2013, Arens 2014).

#### Genus Cleptes

Taxon classificationAnimaliaHymenopteraChrysididae

Latreille, 1802

Figs 11 , 12–17

Cleptes Latreille, 1802: 316.

##### Note.

*Cleptes* females search for tenthredinid and diprionid sawfly cocoons either on the host’s foodplant or on the ground beneath and lay one egg per cocoon (Morgan 1984). The emerging larva develops as an ectoparasitoid of the sawfly prepupa within the cocoon (Darling and Smith 1985, Kimsey and Bohart 1991). The genus consists of around 100 known species, the majority of which occur in the Holarctic Region (Kimsey and Bohart 1991, Wei et al. 2013, Arens 2014). A total of 27 species are known from Europe (Rosa and Soon 2012, Arens 2014) and three from the Nordic and Baltic countries (Paukkunen et al. 2014). We have divided the genus into species-groups according to Móczár (1997, 2001).

##### Key to *Cleptes* species of the Nordic and Baltic countries

**Table:** 

1	Pronotum posteriorly with furrow of foveae (Figs 11, 12). Female mesoscutum metallic golden-red (Fig. 11)	***Cleptes semiauratus* (Linnaeus)**
–	Pronotum without furrow of foveae (Fig. 13). Female mesoscutum black, sometimes with greenish reflections	**2**
2	Vertex with deep postocellar foveae (Fig. 14). Female head black without metallic reflections. Mesoscutum with regular punctation. Meso- and metatibiae yellowish. T2 with dense punctation (Fig. 16). T3 and T4 without metallic sheen, or only laterally with weak violet or bluish reflections (Fig. 16)	***Cleptes nitidulus* (Fabricius)**
–	Vertex without postocellar foveae (Fig. 15). Female head usually with metallic violet reflections. Mesoscutum with only a few punctures. Meso- and metatibiae brownish. T2 with sparse punctation (Fig. 17). T3 and T4 with strong blue or violet metallic sheen (Fig. 17)	***Cleptes semicyaneus* Tournier**

#### *Cleptes nitidulus* group

##### Cleptes nitidulus

Taxon classificationAnimaliaHymenopteraChrysididae

(Fabricius, 1793)

Figs 13 , 14 , 16

Ichneumon nitidulus Fabricius, 1793: 184.
Cleptes nitidula : Fabricius 1804: 154.

###### Diagnosis.

Length 5–7 mm. Both sexes differ from *Cleptes semiauratus* by not having a foveate furrow posteriorly on the pronotum (Fig. 13). The female also differs from *Cleptes semiauratus* by its black head and mesoscutum, non-metallic yellow or orange pronotum, mostly metallic blue mesoscutellum, metanotum and propodeum, and non-metallic black apex of the metasoma. As opposed to the female, the head and mesosoma of the male are entirely metallic green and the apex of the metasoma has faint metallic reflections laterally (Fig. 16). Both sexes differ from *Cleptes semicyaneus* by having pale brown or yellow (not dark brown) legs, denser punctation on the tergites (Fig. 16) and deep postocellar foveae on the vertex (Fig. 14).

###### Distribution.

Denmark, Estonia, Finland, Latvia, Sweden. Rare. – West Palearctic: Europe and Turkey (Linsenmaier 1959), records from China are erroneous (Rosa et al. 2014).

###### Biology.

Habitat: sparsely vegetated sandy areas, such as dry meadows and dunes (Morgan 1984). Occasionally found on flowers of Apiaceae (Heinrich 1964). Flight period: June to August. Host: *Caliroa cerasi* (Linnaeus) and *Euura ribesii* (Scopoli) (Tenthredinidae) (Dahlbom 1854, Morgan 1984).

##### Cleptes semicyaneus

Taxon classificationAnimaliaHymenopteraChrysididae

Tournier, 1879

Figs 15 , 17

Cleptes semicyanea Tournier, 1879: 88.

###### Diagnosis.

Length 4–7 mm. Both sexes resemble *Cleptes nitidulus* superficially, but the legs are darker brown, the punctation of the tergites is sparser (Fig. 17) and the vertex does not have postocellar foveae (Fig. 15). The female differs also from *Cleptes nitidulus* by having blue-violet metallic sheen posteriorly on the metasoma. In the male, this blue-violet sheen is more extensive (Fig. 17) than in *Cleptes nitidulus*.

###### Distribution.

Denmark, Norway, Sweden. Very rare. – Trans-Palearctic: from western Europe to Siberia (Irkutsk) (Móczár 1997).

###### Biology.

Habitat: sparsely vegetated coastal sandy areas (Hansen et al. 2010, Fritz and Larsson 2010). Flight period: June to August. Host: unknown, possibly a sawfly living on creeping willows (*Salix repens* Linnaeus) (Ødegaard et al. 2009).

#### *Cleptes semiauratus* group

##### Cleptes semiauratus

Taxon classificationAnimaliaHymenopteraChrysididae

(Linnaeus, 1761)

Figs 11 , 12

Sphex semiauratus Linnaeus, 1761: 413.
Cleptes semi-auratus : Latreille 1802: 316
Cleptes pallipes Lepeletier, 1806: 119. Synonymised with *Cleptes semiauratus* by Dahlbom (1854) and synonymy reinstated by Rosa et al. (2015).

###### Diagnosis.

Length 5–8 mm. Both sexes differ from *Cleptes nitidulus* and *Cleptes semicyaneus* by having a transverse foveate furrow posteriorly on the pronotum (Figs 11, 12). The female differs also by its metallic golden red head, pronotum, mesoscutum and mesoscutellum (Fig. 11), and distinctly banded wings (Fig. 11). The head and mesosoma of the male are entirely metallic blue-green. The metasoma is anteriorly non-metallic red and posteriorly black with blue reflections in both sexes (Fig. 11).

###### Distribution.

Denmark, Estonia, Finland, Latvia, Lithuania, Norway, Sweden. Relatively rare. – Trans-Palearctic/Holarctic? The general distribution is poorly known due to confusion of *Cleptes semiauratus* with *Cleptes striatipleuris* Rosa, Forshage, Paukkunen & Soon, 2015 (= *Cleptes semiauratus* sensu Lepeletier, 1806) by several authors (Rosa et al. 2015). According to Móczár (2001), *Cleptes semiauratus* has been found in the Palearctic, Nearctic and Oriental Regions (Sumatra). In the Nearctic and Oriental Regions the species has probably been accidentally introduced (Kimsey and Bohart 1991).

###### Biology.

Habitat: forest margins and clearings, gardens and parks. Flight period: June to August. Host: *Endelomyia aethiops* (Gmelin), *Euura ribesii* (Scopoli) and *Pristiphora incisa* (Lindqvist) (Tenthredinidae) (Alfken 1915, Burger and Sobczyk 2011, V. Vikberg, pers. obs.). Several other tenthredinid sawfly species reported as hosts for *Cleptes semiauratus* might actually represent hosts of *Cleptes striatipleuris*.

### II. Subfamily Chrysidinae

The majority of all chrysidids, about 80%, belong to this subfamily (Kimsey and Bohart 1991). Its members are characterised by a bright metallic colouration (with a few rare exceptions), three (or less commonly two or four) external tergites, and a concave or flat metasomal venter (with one rare exception). They are mainly nest parasites of solitary wasps and bees, although one genus, *Praestochrysis*, attacks prepupal moth larvae. The subfamily is distributed in all zoogeographical regions and consists of four tribes: Allocoelini (not present in Europe), Elampini, Chrysidini and Parnopini (Kimsey and Bohart 1991, Carpenter 1999).

#### Tribe Elampini

Chrysidid wasps of this tribe are characterised by three external metasomal tergites, the absence of a pit row or sublateral foveae on T3, and the usually dentate tarsal claw. The tribe has a worldwide distribution, though most of the genera and species occur in arid areas of the Holarctic Region. A total of 21 genera are recognised, seven of which are found in North Europe.

##### Genus Omalus

Taxon classificationAnimaliaHymenopteraChrysididae

Panzer, 1801

Figs 18 , 19–25

Omalus Panzer, 1801: 13.

###### Note.

Many authors have treated this genus in the broad sense and divide it into several subgenera (see summary by Rosa 2006). We follow the classification of Kimsey and Bohart (1991), whereby *Elampus*, *Philoctetes* and *Pseudomalus* are recognised as valid genera. *Omalus* sensu stricto is characterised by the following morphological features: pronotum and mesoscutum without or with small punctures which are arranged evenly over the entire surface; mesopleuron projecting ventrally weakly, its lateroventral margin forming an obtuse angle in lateral view (Fig. 19); genal carina bisecting malar space. The larvae develop as parasitoids of crabronid wasps of the subfamily Pemphredoninae. Currently, 26 species are recognised worldwide, most of which are found in the Holarctic Region (Kimsey and Bohart 1991, Wei et al. 2014). A total of eight species are found in Europe (Rosa and Soon 2012), and three are known from the Nordic and Baltic countries (Paukkunen et al. 2014). The status of several European taxa is uncertain, and the genus is in need of taxonomic revision.

**Figure 18.**
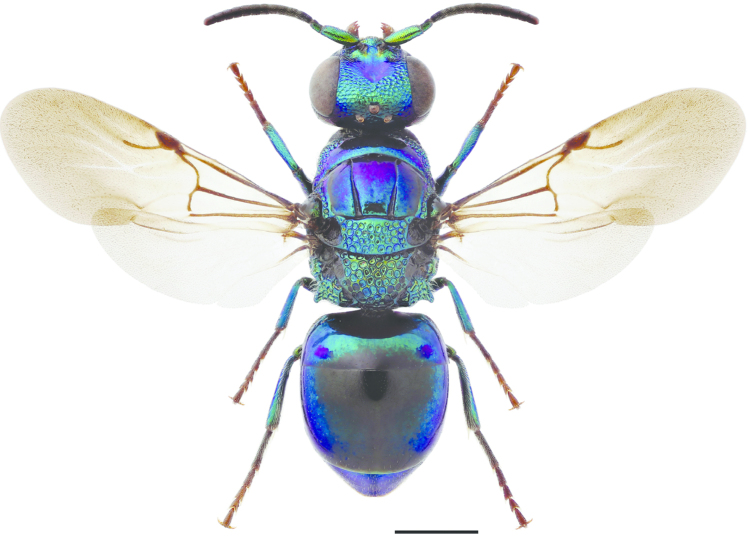
*Omalus aeneus* ♀. Scale 1 mm.

###### Key to *Omalus* species of the Nordic and Baltic countries

**Table:** 

1	Mesoscutum wrinkled and dull, without punctures (Fig. 20). Apical notch of T3 deep and triangular (Fig. 21). Body bicoloured with blue, violet or black head and mesosoma, and contrastingly greenish, golden or reddish metasoma	***Omalus biaccinctus* (du Buysson)**
–	Mesoscutum smooth and shining, without punctures or with evenly distributed punctures (Figs 22, 24). Apical notch smaller (Figs 23, 25). Body concolorous green, blue, violet or mostly black	**2**
2	Mesoscutum mostly with tiny punctures or impunctate (Fig. 22) and only laterally with short pubescence. If punctures coarser, then flagellomeres slightly longer than broad. Setae laterally on pronotum less than twice as long as diameter of mid-ocellus. Apical notch of T3 shallow (Fig. 23). Flagellomeres as long as or slightly longer than broad	***Omalus aeneus* (Fabricius)**
–	Mesoscutum with relatively coarse punctures (Fig. 24) and long pubescence. Setae laterally on pronotum at least twice as long as diameter of mid-ocellus. Apical notch of T3 relatively deep (Fig. 25). Flagellomeres short, not longer than broad	***Omalus puncticollis* (Mocsáry)**

##### Omalus biaccinctus

Taxon classificationAnimaliaHymenopteraChrysididae

(du Buysson, 1892)

Figs 20 , 21

Ellampus biaccinctus du Buysson (in André), 1892: 152.
Omalus biaccinctus : Trautmann 1927: 41.

###### Diagnosis.

Length 3–5 mm. Both sexes differ from *Omalus aeneus* and *Omalus puncticollis* by having a bicoloured body (head and mesosoma violet or black, mesosoma reddish or greenish) and a dull and wrinkled mesoscutum without punctures (Fig. 20). The apical notch of T3 is also more deeply triangular (Fig. 21) than in the other two species. The colour of the mesosoma is violet in the female, but dorsally black or dark violet in the male. The metasoma is greenish, golden or reddish in the female, whereas it is greenish, rarely golden or reddish, and dorsally usually black in the male.

###### Distribution.

Denmark, Estonia, Finland, Latvia, Norway, Sweden. Relatively rare. – West Palearctic: from western Europe to western Asia (Linsenmaier 1959).

###### Biology.

Habitat: pine forest margins and clearings, semi-open sandy areas. Occasionally found on flowers of Apiaceae and Asteraceae (Kofler 1975, Rosa 2004). Flight period: June to August. Host: *Passaloecus turionum* Dahlbom, *Passaloecus gracilis* (Curtis) and *Passaloecus eremita* Kohl (Crabronidae) (Lomholdt 1975, Tormos et al. 1996, Wickl 2001, our own obs.). Adults have been reared from old resin-galls of *Retinea resinella* (Linnaeus) (Tortricidae) (V. Vikberg, pers. obs.) and pieces of pine wood (Kofler 1975) with host nests inside. The females oviposit in living aphids at the hunting site of their host, and the egg is brought into the host’s nest concealed in the aphid prey (Winterhagen 2015). Thus, the females do not enter the nest of their host for oviposition.

**Figures 19–25.**
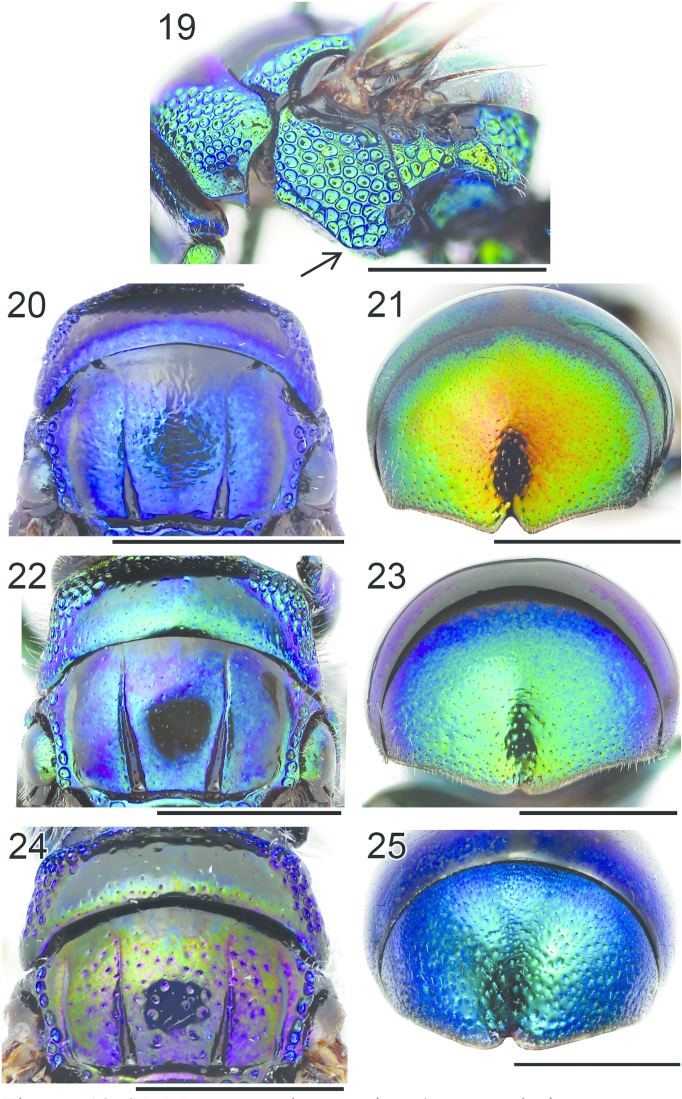
Mesosoma, lateral view (arrow indicating mesopleuron): **19** *Omalus aeneus* ♀. Pronotum and mesoscutum, dorsal view: **20** *Omalus biaccinctus* ♀ **22** *Omalus aeneus* ♀ **24** *Omalus puncticollis* ♀. T3, postero-dorsal view: **21** *Omalus biaccinctus* ♀ **23** *Omalus aeneus* ♀ **25** *Omalus puncticollis* ♀. Scale 1 mm.

##### Omalus aeneus

Taxon classificationAnimaliaHymenopteraChrysididae

(Fabricius, 1787)

Figs 18 , 19 , 22 , 23

Chrysis aenea Fabricius, 1787: 284.
Omalus aeneus : Panzer 1801: 13.

###### Diagnosis.

Length 3–6 mm. The species resembles closely *Omalus puncticollis*, but usually has only very small punctures and short pubescence on the mesoscutum (Figs 18, 22), sparser and finer punctation on the pronotum (Figs 18, 22), and a shallower apical notch on T3 (Fig. 23). Some specimens have relatively coarse punctation medially on the pronotum and mesoscutum, but compared to *Omalus puncticollis* their pubescence is shorter, flagellomeres are longer, and the apical notch of T3 is shallower. In the female, the body is completely deep blue, violet or green (Fig. 18), whereas in the male it is dorsally black and laterally with green or blue reflections.

###### Distribution.

Denmark, Estonia, Finland, Latvia, Lithuania, Norway, Sweden. Common. – Trans-Palearctic/Holarctic: from western Europe and northern Africa to Japan, China and Taiwan. Possibly accidentally introduced to North America (Kimsey and Bohart 1991) and Australia (Krombein 1979).

###### Biology.

Habitat: forest margins and clearings, *semi*-open sandy areas. Adults are often observed on sun-exposed leaves of trees and bushes, and they are attracted to honeydew of aphids. Occasionally they are also found on flowers of Apiaceae and Euphorbiaceae (Trautmann 1927, Hoop 1971, Linsenmaier 1997). Flight period: June to August. Host: *Passaloecus corniger* Shuckard, *Passaloecus eremita* Kohl, *Passaloecus gracilis* (Curtis), *Passaloecus singularis* Dahlbom, *Passaloecus turionum* Dahlbom, *Pemphredon lethifer* (Shuckard), *Pemphredon lugubris* (Fabricius) and *Psenulus pallipes* (Panzer) (Crabronidae) (Barbey and Ferriere 1923, Strumia 1997, Gathmann and Tscharntke 1999, our own obs.). The species has been reared from old resin-galls of *Retinea resinella* (Tortricidae) containing host nests. Females oviposit in live aphids and do not enter the host nest (our own obs.). A similar behaviour has been observed in *Omalus biaccinctus* (Winterhagen 2015).

###### Remarks.

Mitochondrial DNA studies indicate that the Nordic and Baltic specimens of *Omalus aeneus* belong to at least five genetically distinct lineages (excl. *Omalus puncticollis*), and several other lineages have been found in other countries (Paukkunen et al. 2014). It is very likely that more than one species is involved.

##### Omalus puncticollis

Taxon classificationAnimaliaHymenopteraChrysididae

(Mocsáry, 1887)

Figs 24 , 25

Ellampus puncticollis Mocsáry, 1887: 291.
Omalus puncticollis : Morgan 1984: 16.

###### Diagnosis.

Length 3–6 mm. The species is easily confused with *Omalus aeneus*, but the mesoscutum always has relatively large scattered punctures and long setae (Fig. 24). The pronotum has also larger punctures medially (Fig. 24) and the apical notch of T3 is deeper (Fig. 25). The body colouration is similar to *Omalus aeneus*. Habitus of large specimens can sometimes resemble small specimens of *Pseudomalus violaceus*, but the ventral margin of the mesopleuron is not as strongly projecting in *Omalus puncticollis* (as in Fig. 19) and the large punctures of the mesoscutum are not clumped postero-medially.

###### Distribution.

Norway, Sweden. Rare. – West Palearctic (?): Europe, Turkey and northern Africa (Linsenmaier 1959, 1968, 1999). The general distribution is poorly known, because many authors have considered *Omalus puncticollis* to be conspecific with *Omalus aeneus*.

###### Biology.

Habitat: forest margins and clearings, *semi*-open sandy areas. Adults are usually found sitting on or flying near leaves of trees and bushes, occasionally also on flowers of Apiaceae (Kunz 1994). Flight period: June to August. Host: *Passaloecus gracilis* (Curtis), *Passaloecus eremita* Kohl, *Passaloecus corniger* Shuckard and *Passaloecus turionum* Dahlbom (Crabronidae) (Spooner 1954, Gauss 1967, Mocsáry 1912).

###### Remarks.

Mitochondrial DNA studies support the recognition of *Omalus puncticollis* as a distinct species in relation to *Omalus aeneus* (Paukkunen et al. 2014).

##### Genus Pseudomalus

Taxon classificationAnimaliaHymenopteraChrysididae

Ashmead, 1902

Figs 2 , 26 , 27–34

Pseudomalus Ashmead, 1902: 229.

###### Note.

This taxon was raised to generic rank by Kimsey and Bohart (1991). It is characterised by the structure and punctation of the mesosoma: the large punctures are clumped posteriorly between the notauli on the mesoscutum (Fig. 27), and the lateroventral margin of the mesopleuron is strongly projecting ventrally, forming a sharp angle in lateral view (Fig. 28). The posterior margin of T3 is usually deeply notched medially (Figs 31, 32). *Pseudomalus* is a Holarctic genus with approximately 40 recognised species (Kimsey and Bohart 1991). The larvae are parasitoids of crabronid wasps of the subfamily Pemphredoninae. The European fauna consists of ten species (Rosa and Soon 2012), of which four have been found in the Nordic and Baltic countries (Paukkunen et al. 2014).

**Figure 26.**
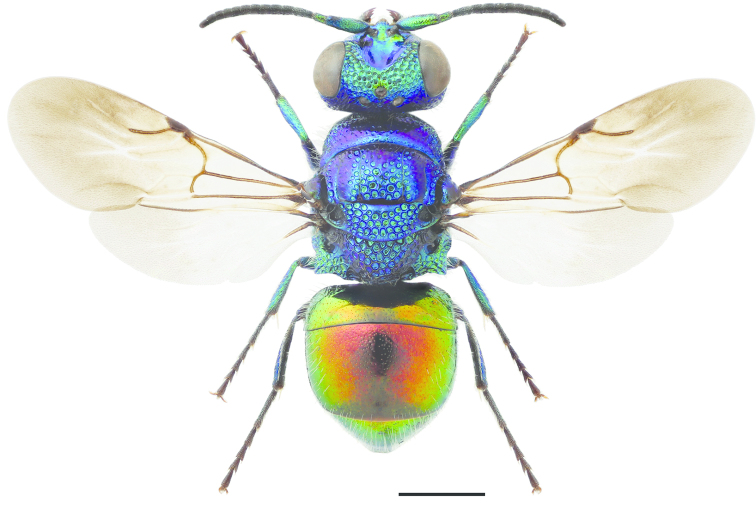
*Pseudomalus auratus* ♀. Scale 1 mm.

###### Key to *Pseudomalus* species of the Nordic and Baltic countries

**Table:** 

1	Body entirely green to greenish-golden, violet-blue or blackish-green	**2**
–	Body bicoloured with greenish to bluish head and mesosoma and at least laterally red metasoma	**3**
2	Body entirely green or green-golden, usually with golden reflections on mesoscutum and mesoscutellum. Metascutellum sharply convex (Fig. 29). Head and mesosoma with short and sparse pubescence, setae not more than twice as long as diameter of mid-ocellus. Smaller species, body length 3–5 mm	***Pseudomalus pusillus* (Fabricius)**
–	Body uniformly violet-blue or black-green. Metascutellum rounded (Fig. 30). Setae more than twice as long as diameter of mid-ocellus. Larger species, body length 5–7 mm	***Pseudomalus violaceus* (Scopoli)**
3	Antenna long and slender, medial flagellomeres longer than broad (Fig. 34). Larger species, body length normally not less than 5 mm. Apical notch of T3 triangular, not deeper than broad (Fig. 31)	***Pseudomalus triangulifer* (Abeille de Perrin)**
–	Antenna shorter, medial flagellomeres approximately as long as broad (Fig. 33). Smaller species, body length usually 3–5 mm, exceptionally up to 6 mm. Apical notch of T3 dorsally arched, as deep as or deeper than broad (Fig. 32)	***Pseudomalus auratus* (Linnaeus)**

##### Pseudomalus pusillus

Taxon classificationAnimaliaHymenopteraChrysididae

(Fabricius, 1804)

Fig. 29

Chrysis pusilla Fabricius, 1804: 176.
Pseudomalus pusillus : Kimsey and Bohart 1991: 268.

###### Diagnosis.

Length 3–5 mm. The species differs from other *Pseudomalus* species by having an entirely green, green-golden or green-blue body with usually golden reflections on the mesoscutum, mesoscutellum and metanotum. Dark specimens can be confused with unusually dark specimens of *Pseudomalus auratus*, but the apex of the metasoma protrudes more narrowly, the metascutellum is more elevated medially (Fig. 29) and the pubescence is shorter.

###### Distribution.

Denmark, Latvia and Lithuania. Very rare. – Trans-Palearctic: from western Europe and northern Africa to Russian Far East (Kurzenko and Lelej 2007).

###### Biology.

Habitat: sparsely vegetated sandy areas, such as river banks and dunes. Adults are attracted to honeydew of aphids (Trautmann 1927) and they are occasionally found on flowers of Apiaceae, Asteraceae, Euphorbiaceae and Resedaceae (Kusdas 1956, Rosa 2004). Flight period: June to August. Host: *Passaloecus eremita* Kohl, *Passaloecus insignis* (Vander Linden) and *Pemphredon lethifer* (Shuckard) (Crabronidae) (Benno 1950, Wickl 2001). We consider records mentioning other crabronids (e.g. *Rhopalum coarctatum* (Scopoli) and species of *Trypoxylon* Latreille) as hosts to be uncertain, because their biology is quite different from other hosts.

**Figures 27–34.**
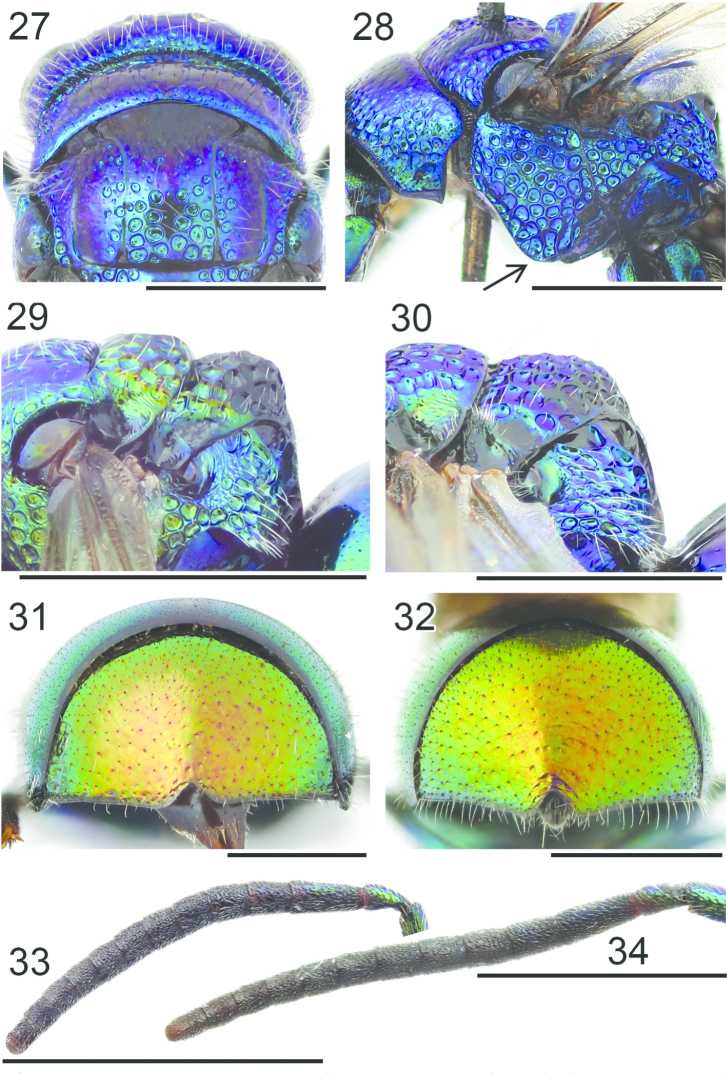
Pronotum and mesoscutum, dorsal view: **27** *Pseudomalus auratus* ♀. Mesosoma, lateral view (arrow indicating mesopleuron): **28** *Pseudomalus auratus* ♀. Mesoscutellum, metanotum and propodeum, lateral view: **29** *Pseudomalus pusillus* ♀ **30** *Pseudomalus violaceus* ♀. T3, postero-dorsal view: **31** *Pseudomalus triangulifer* ♀ **32** *Pseudomalus auratus* ♀. Antenna: **33** *Pseudomalus auratus* ♀ **34** *Pseudomalus triangulifer* ♀. Scale 1 mm.

##### Pseudomalus auratus

Taxon classificationAnimaliaHymenopteraChrysididae

(Linnaeus, 1758)

Figs 26 , 27 , 28 , 32 , 33

Sphex aurata Linnaeus, 1758: 572.
Pseudomalus auratus : Kimsey and Bohart 1991: 265.

###### Diagnosis.

Length 3–6 mm. Both sexes have a bicoloured body with a blue-green or violet head and mesosoma, and a red (or rarely entirely greenish) metasoma with green reflections (Fig. 26). The species is very similar to *Pseudomalus triangulifer*, but the antennal segments are shorter (Fig. 33) and the body is usually smaller. The apical notch of T3 is also deeper and more rounded dorsally (Fig. 32).

###### Distribution.

Denmark, Estonia, Finland, Latvia, Lithuania, Norway, Sweden. Common. – Trans-Palearctic/Holarctic: from western Europe and northern Africa to China, Korea and Japan. Introduced accidentally to North America (Kimsey and Bohart 1991).

###### Biology.

Habitat: forest margins and clearings, gardens and parks. Often found on sun-exposed leaves of deciduous trees and bushes. Adults are attracted to honeydew of aphids and occasionally visit flowers of Apiaceae and Euphorbiaceae (Rosa 2004, our own obs.). Flight period: May to August. Host: cavity-nesting crabronid wasps that prey on aphids, e.g. *Passaloecus corniger* Shuckard, *Passaloecus eremita* Kohl, *Passaloecus insignis* (Vander Linden), *Passaloecus gracilis* (Curtis), *Passaloecus monilicornis* Dahlbom, *Passaloecus singularis* Dahlbom, *Passaloecus turionum* Dahlbom, *Pemphredon inornata* Say, *Pemphredon lethifer* (Shuckard), *Passaloecus lugens* Dahlbom, *Pemphredon lugubris* (Fabricius) and *Passaloecus rugifer* (Dahlbom) (Schenck 1856, Benno 1957, van Lith 1958, Brechtel 1986, Blösch 2002, our own obs.), but also *Diodontus tristis* (Vander Linden), which is a soil-nesting species (Blösch 2002). Host records mentioning other crabronids, such as species of *Rhopalum* Stephens, *Trypoxylon* Latreille and *Crabro* Fabricius, are questionable, because the prey does not consist of aphids in these taxa. Females oviposit in aphids before they have been captured and brought to the nest by the host (our own obs.). A similar behaviour has been observed also in *Omalus biaccinctus* (Winterhagen 2015) and postulated for *Pseudomalus triangulifer* (Veenendaal 2011).

##### Pseudomalus triangulifer

Taxon classificationAnimaliaHymenopteraChrysididae

(Abeille de Perrin, 1877)

Figs 2 , 31 , 34

Omalus triangulifer Abeille de Perrin, 1877: 65.
Pseudomalus triangulifer : Kimsey and Bohart 1991: 269.

###### Diagnosis.

Length 6–7 mm. The species resembles closely *Pseudomalus auratus*, but the antennal segments are longer (Fig. 34), the body is usually larger and the shape of the apical notch of T3 is shallower and more triangular (Fig. 31). The colour of the metasoma varies from mostly red to almost green. The darkest specimens can be somewhat similar to *Pseudomalus violaceus*, but the apical notch is always deeper in *Pseudomalus triangulifer*.

###### Distribution.

Denmark, Estonia, Finland, Latvia, Lithuania, Norway, Sweden. Relatively rare. – Trans-Palearctic: from Europe and Turkey to China (Linsenmaier 1959, 1968, Rosa et al. 2014).

###### Biology.

Habitat: forest margins and clearings, gardens and parks. Often collected from sun-exposed leaves of trees and bushes. Adults are attracted to honeydew of aphids and occasionally also to flowers of Apiaceae and Euphorbiaceae (Linsenmaier 1997). Flight period: late April to August. Host: *Passaloecus insignis* (Vander Linden), *Pemphredon lugubris* (Fabricius), *Pemphredon lethifer* (Shuckard), *Pemphredon lugens* Dahlbom, *Pemphredon montana* Dahlbom and *Pemphredon rugifer* (Dahlbom) (Crabronidae) (Alfken 1915, Strumia 1996, Wickl 2001, Veenendaal 2011, our own obs.). Females probably oviposit in aphids before they have been captured and brought to the nest by the host (Veenendaal 2011).

##### Pseudomalus violaceus

Taxon classificationAnimaliaHymenopteraChrysididae

(Scopoli, 1763)

Fig. 30

Sphex violacea Scopoli, 1763: 298.
Chrysis micans Olivier, 1791: 677.
Chrysis fuscipennis Dahlbom, 1829: 15.
Chrysis coerulea Dahlbom, 1831: 33.
Pseudomalus violaceus : Kimsey and Bohart 1991: 270.

###### Diagnosis.

Length 5–8 mm. The species differs from other species of the genus by its completely violet-blue (female) or black-green to black-blue (male) body, and a wide and shallow apical notch on T3. The scapal basin is also higher and dorsally deeply angled. Exceptionally small and worn specimens can be confused with *Omalus puncticollis* (or *Omalus aeneus*), but the mesopleuron of *Pseudomalus violaceus* always strongly projects ventrally (as in Fig. 28) and the mesoscutum has large punctures, which are clumped postero-medially (as in Fig. 27).

###### Distribution.

Denmark, Estonia, Finland, Latvia, Lithuania, Norway, Sweden. Relatively rare. – Trans-Palearctic (?). Europe, Middle East, Siberia, Manchuria (Linsenmaier 1997). Eastern records could be related to *Pseudomalus bergi* Semenov, 1932 (Rosa et al. 2014) or other similar central Asiatic species, e.g. *Pseudomalus bogojavlenskii* Semenov, 1932 or *Pseudomalus saturatus* Semenov, 1932.

###### Biology.

Habitat: forest margins and clearings. Often found on leaves of sun-exposed deciduous trees and bushes. Flight period: June to August. Adults are attracted to honeydew of aphids (Gauss 1987). Host: *Pemphredon lugubris* (Fabricius), more rarely also *Passaloecus corniger* Shuckard and *Passaloecus eremita* Kohl (Crabronidae) (Nielsen 1900, Morgan 1984, Gathmann and Tscharntke 1999, our own obs.). Host records of other species (e.g. *Trypoxylon* Latreille) are doubtful, because of their deviant biology compared to other hosts. In Finland, the species has been reared from an old gall of *Saperda populnea* (Linnaeus) (Cerambycidae) on a *Populus* branch (M. Pentinsaari pers. obs.) and a rotten *Alnus* stump containing host nests.

##### Genus Philoctetes

Taxon classificationAnimaliaHymenopteraChrysididae

Abeille de Perrin, 1879

Figs 35 , 36

Philoctetes Abeille de Perrin, 1879: 27.

###### Note.

The taxonomic rank and delineation of *Philoctetes* has differed among several authors. We follow the definition given by Kimsey and Bohart (1991) and consider it as a distinct genus. *Philoctetes* is characterised by the following diagnostic features: mesoscutum with large punctures concentrated along notauli; central malar space without carina; mesopleuron rounded and weakly projecting ventrally; metascutellum usually conical; posterior margin of T3 usually deeply notched medially. The hosts consist of crabronid wasps of the subfamily Pemphredoninae. Approximately 40 species are recognised worldwide, and about 30 of these are Palearctic (Kimsey and Bohart 1991). A total of 22 species are known from Europe (Rosa and Soon 2012), but only *Philoctetes truncatus* is found in the Nordic and Baltic countries (Paukkunen et al. 2014).

**Figure 35.**
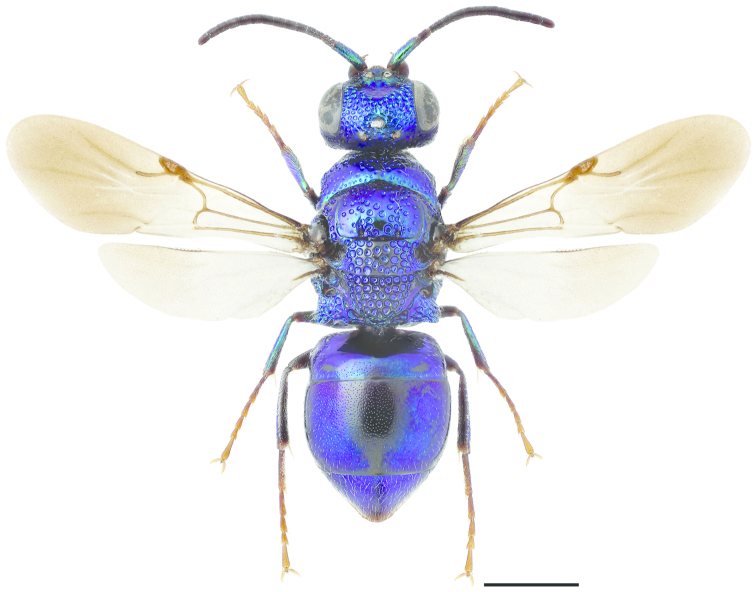
*Philoctetes truncatus* ♀. Scale 1 mm.

##### Philoctetes truncatus

Taxon classificationAnimaliaHymenopteraChrysididae

(Dahlbom, 1831)

Figs 35 , 36

Chrysis truncata Dahlbom, 1831: 35.
Elampus coeruleus of authors, not Dahlbom, 1854.
Philoctetes truncatus : Kimsey and Bohart 1991: 258.

###### Diagnosis.

**Figure 36.**
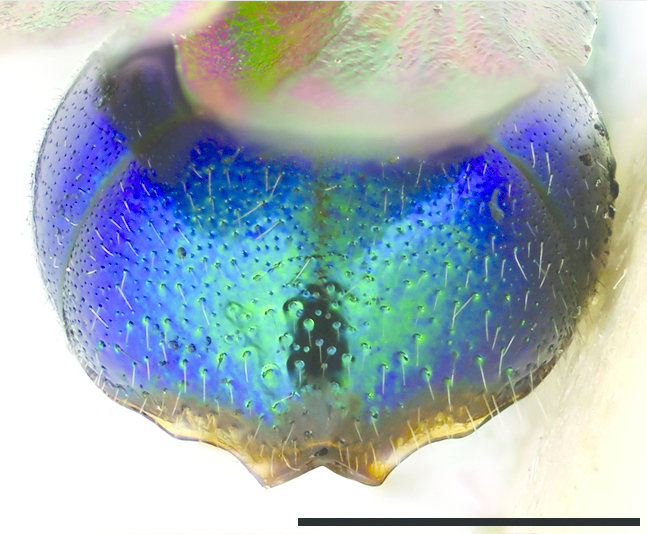
*Philoctetes truncatus* ♀ T3, postero-dorsal view. Scale 1 mm.

Length 3–5 mm. The species resembles *Omalus aeneus* and *Omalus puncticollis* by its habitus and colouration. In the female, the body is completely shiny deep blue, violet or green (Fig. 35), whereas it is mainly black with green or blue reflections in the male. Compared to *Omalus* species, the metascutellum is more sharply elevated and the punctures on the mesoscutum are larger. The apical notch of T3 is shallowly triangular and bordered by a thickened margin (Fig. 36). The lateral margins of T3 are semitransparent and strongly convex adjacent to the apical notch (Fig. 36).

###### Distribution.

Denmark, Estonia, Latvia, Sweden. Rare. New to Latvia (1 ♀, Jekabpils, Avotu iela, 7.–30.VI.2006, leg. P.N. Buhl). – Trans-Palearctic: from western Europe and northern Africa to Russian Far East (Kurzenko and Lelej 2012).

###### Biology.

Habitat: sparsely vegetated sandy areas, sandstone and loess banks (Heinrich 1964, our own obs.). Adults occasionally visit flowers of Apiaceae (Trautmann 1927, Linsenmaier 1997). Flight period: June to July. Host: *Diodontus tristis* (Vander Linden) (Crabronidae) (Hoop 1961, Linsenmaier 1997, Saure 1998, Jacobs and Kornmilch 2007, our own obs.).

##### Genus Elampus

Taxon classificationAnimaliaHymenopteraChrysididae

Spinola, 1806

Figs 37 , 38–46

Elampus Spinola, 1806: 10.
Ellampus Agassiz, 1846: 136.
Notozus Förster, 1853: 351.

###### Note.

This genus has been treated as a subgenus of *Omalus* by some authors (e.g. Linsenmaier 1959, 1997). It is well characterised by the shape of the metascutellum, which has a large tongue-like projection dorsally (Fig. 38). The posterior margin of T3 is usually extended into a horseshoe-shaped or falcate rim forming truncation (Figs 39–40, 44–46). The female has a row of dense and erect setae along the genal margin (Fig. 41). These setae are replaced by long irregularly placed bristles in the male. The hosts are ground-nesting crabronid wasps, such as *Mimesa* Shuckard and *Mimumesa* Malloch (Kimsey and Bohart 1991). The genus is distributed in the Palearctic Region (more than 40 species), North America (8 species), Africa (7 species) and South America (3 species) (Kimsey and Bohart 1991, Linsenmaier 1999, Madl and Rosa 2012). A total of 12 species have been found in Europe (Rosa and Soon 2012), and three of these occur in the Nordic and Baltic countries (Paukkunen et al. 2014).

**Figure 37.**
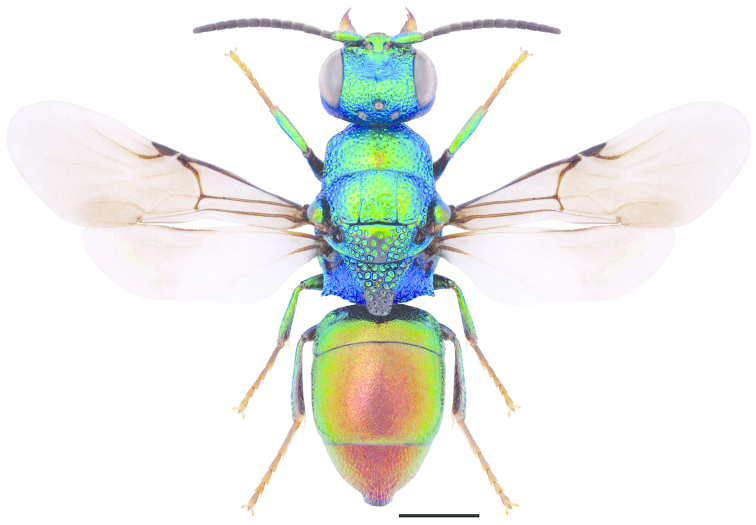
*Elampus panzeri* ♀. Scale 1 mm.

###### Key to *Elampus* species of the Nordic and Baltic countries

**Table:** 

1	Lateral margin of T3 with a narrow notch between apical truncation and semitransparent lateral protrusion (Fig. 39). Apical truncation of T3 horseshoe-shaped with nearly straight ventral margins (Fig. 44)	***Elampus panzeri* (Fabricius)**
–	Lateral margin of T3 slightly convex or almost straight between apical truncation and *semi*-transparent lateral protrusion (Fig. 40). Apical truncation with pointed or rounded ventral margins (Figs 45, 46)	**2**
2	Apical truncation falcate with pointed margins ventrally (Fig. 45). Punctation of T2 dense and regular. Scrobal carina reaches omaulus at its anterior corner (Fig. 42). First flagellomere of female approximately four times as long as broad	***Elampus constrictus* (Förster)**
–	Apical truncation horseshoe-shaped with rounded margins ventrally (Fig. 46). Punctation of T2 dense, usually with an impunctate central line anteriorly. Scrobal carina reaches omaulus below its anterior protrusion (Fig. 43). First flagellomere of female approximately three times as long as broad	***Elampus foveatus* (Mocsáry)**

##### Elampus constrictus

Taxon classificationAnimaliaHymenopteraChrysididae

(Förster, 1853)

Figs 42 , 45

Notozus constrictus Förster, 1853: 336.
Elampus productus of authors, not Dahlbom, 1854.
Ellampus spina of authors, not (Lepeletier, 1806).
Notozus panzeri of authors, not (Fabricius, 1804).
Elampus constrictus : Morgan 1984: 7.

###### Diagnosis.

Length 4–7 mm. The species differs from *Elampus panzeri* and *Elampus foveatus* by the structure of the apical truncation of T3, which has pointed margins ventrally and resembles a sickle or a boomerang in shape (Fig. 45). Sometimes the apical truncation is very narrow, similar to that of *Philoctetes truncatus*. The lateral margins of T3 are shallowly concave anterior to the apical truncation. As opposed to *Elampus panzeri* and *Elampus foveatus*, the scrobal carina reaches the angle of the omaulus (Fig. 42). Both sexes are usually bicoloured with a blue or greenish head and mesosoma, and a reddish metasoma. Entirely greenish or blue specimens are found occasionally.

###### Distribution.

Denmark, Estonia, Finland, Norway, Sweden. Relatively rare. – Trans-Palearctic: widely distributed in the Palearctic Region, from Europe to China (Rosa et al. 2014).

###### Biology.

Habitat: sparsely vegetated sandy areas, heaths. Adults occasionally visit flowers of Apiaceae and Rosaceae (Linsenmaier 1997, Rosa 2004). Flight period: May to July. Host: *Mimesa bicolor* (Jurine), *Mimesa equestris* (Fabricius) and *Mimesa lutaria* (Fabricius) (Crabronidae) (Benno 1950, Lomholdt 1975).

**Figures 38–46.**
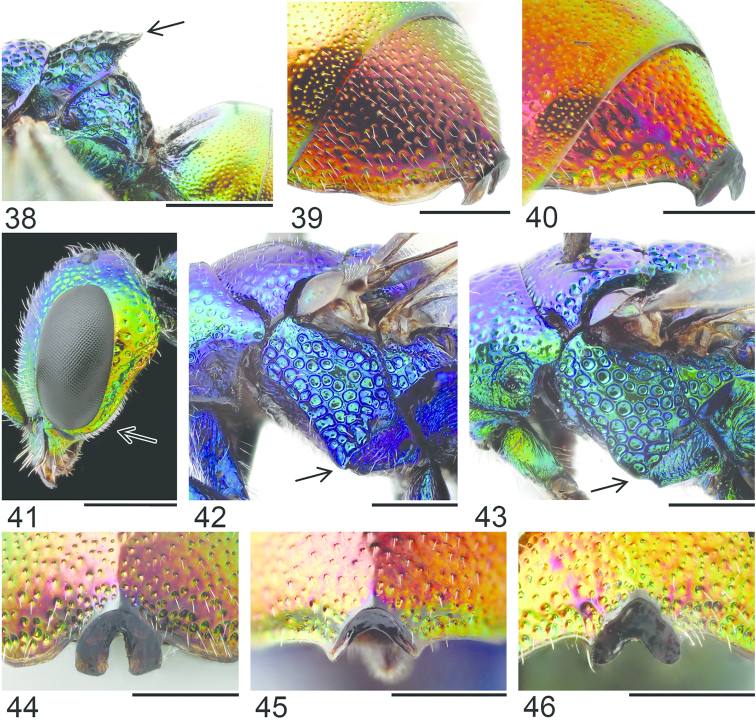
Metascutellum, propodeum and T1, lateral view (arrow indicating metascutellar projection): **38** *Elampus foveatus* ♀. T3, lateral view: **39** *Elampus panzeri* ♀ **40** *Elampus foveatus* ♀. Head, lateral view (arrow indicating genal setae): **41** *Elampus panzeri* ♀. Mesopleuron, lateral view (arrow indicating junction of omaulus and scrobal carina): **42** *Elampus constrictus* ♀ **43** *Elampus foveatus* ♀. T3, postero-dorsal view: **44** *Elampus panzeri* **45** *Elampus constrictus* ♀ **46** *Elampus foveatus* ♀. Scale 0.5 mm.

##### Elampus foveatus

Taxon classificationAnimaliaHymenopteraChrysididae

(Mocsáry, 1914)

Figs 38 , 40 , 43 , 46

Ellampus foveatus Mocsáry, 1914: 1.
Elampus foveatus : Kimsey and Bohart 1991: 168.

###### Diagnosis.

Length 5–8 mm. The species can be confused with *Elampus constrictus* and *Elampus panzeri*, but the apical truncation of T3 has rounded margins ventrally and resembles a thick, upside-down U in shape (Fig. 46). The lateral margins of T3 are similar to *Elampus constrictus* (Fig. 40). The punctation of T2 is somewhat more irregular than in *Elampus constrictus*, and usually an impunctate medial line is formed anteriorly. The scrobal carina ends below the angle of the omaulus (Fig. 43) as in *Elampus panzeri*. The head and mesosoma are blue or greenish, and the metasoma is red with green reflections in both sexes.

###### Distribution.

Estonia, Finland, Norway, Sweden. Rare. – Trans-Palearctic: from the Netherlands to Siberia (Usolye-Sibirskoye). The distribution is still poorly known, because many authors have confused *Elampus foveatus* with other closely related taxa.

###### Biology.

Habitat: sparsely vegetated sandy areas. In Germany, specimens have been found on *Sambucus* bushes (Niehuis and Gauss 1996). Flight period: May to July. Host: unknown.

##### Elampus panzeri

Taxon classificationAnimaliaHymenopteraChrysididae

(Fabricius, 1804)

Figs 37 , 39 , 41 , 44

Chrysis scutellaris Panzer, 1798: 11, not Fabricius, 1794.
Chrysis Panzeri Fabricius, 1804: 172, replacement name for *Chrysis scutellaris* Panzer, 1798.
Elampus Panzeri : Latreille 1809: 45.
Notozus constrictus of authors, not Förster, 1853.

###### Diagnosis.

Length 4–8 mm. The species resembles *Elampus constrictus* and *Elampus foveatus*, but the apical truncation of T3 has angular margins ventrally and resembles a horseshoe in shape (Fig. 44). The lateral margins of T3 also have narrow notches in front of the apical truncation (Fig. 39). The punctation of T2 is somewhat sparser than in *Elampus constrictus* and *Elampus foveatus*. The scrobal carina is similar to *Elampus foveatus* (Fig. 43). Both sexes are bicoloured with a green or blue head and mesosoma, and a red metasoma with green reflections (Fig. 37). Rarely the metasoma can be entirely greenish.

###### Distribution.

Denmark, Estonia, Finland, Latvia, Lithuania, Norway, Sweden. Common. – Trans-Palearctic: Europe, western Asia, Manchuria (Linsenmaier 1959, as *Elampus constrictus*).

###### Biology.

Habitat: sparsely vegetated sandy areas, heaths. Adults are occasionally found on flowers of Apiaceae (Heinrich 1964) and stems and inflorescences of grasses (Trautmann 1927, our own obs.). Flight period: late May to August. Adults are also attracted to honeydew of aphids. Host: *Mimesa equestris* (Fabricius) and *Mimesa lutaria* (Fabricius) (Crabronidae) (Morice 1903, Benno 1950, our own obs.).

###### Remarks.

The names *Elampus constrictus* and *Elampus panzeri* were erroneously swapped by Trautmann (1927) and later by Linsenmaier (1959, 1997) and other authors. See details from Móczár (1964).

##### Genus Holopyga

Taxon classificationAnimaliaHymenopteraChrysididae

Dahlbom, 1845

Figs 3 , 7 , 47 , 48–52

Holopyga Dahlbom, 1845: 4.

###### Note.

This genus consists mainly of broad-bodied wasps, with a body length of 4–9 mm. Morphological characters of the genus include the strongly curved medial vein of the forewing (Fig. 7), the setose medial cell of the forewing, the multidentate tarsal claw (Fig. 3), the carinate and angulate mesopleuron and the evenly rounded posterior margin of T3 (without any distinct notches or prominences). Some species are sexually dimorphic with contrasting colouration in the different sexes (e.g. *Holopyga fervida*). The biology of most species is poorly known. Apparently, the hosts consist of ground-nesting crabronid and sphecid wasps. Records stating megachilid solitary bees as hosts are questionable due to the lack of supporting data. *Holopyga* is a large genus with more than 90 recognised species worldwide. The vast majority of these, nearly 70 species, occur in the Palearctic Region (Kimsey and Bohart 1991, Arens 2004). A total of 43 species are known from Europe, and four have been found in the Nordic and Baltic countries (Rosa and Soon 2012, Paukkunen et al. 2014). We have divided the genus into species-groups according to Linsenmaier (1959).

**Figure 47.**
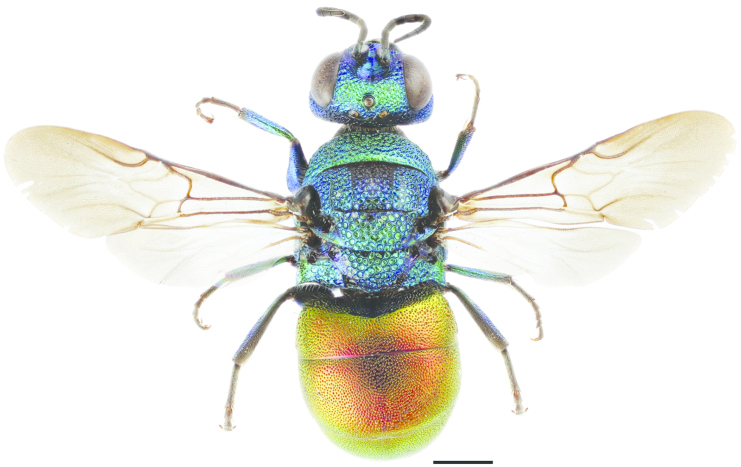
*Holopyga generosa* ♂. Scale 1 mm. (Photo: Pekka Malinen)

###### Key to *Holopyga* species of the Nordic and Baltic countries

**Table:** 

1	Head and mesosoma entirely green-bluish, metasoma dorsally red (Fig. 47)	***Holopyga generosa* (Förster)**
–	Mesosoma dorsally red, or if green-bluish or golden green, then metasoma of the same colour	**2**
2	Head entirely blue, pronotum, mesoscutum, mesoscutellum and metascutellum dorsally red, rest of mesosoma blue, metasoma dorsally red	***Holopyga inflammata* (Förster)**
–	Head dorsally red, or if green or blue, then whole body green-blue or golden green	**3**
3	Head and mesosoma partially dorsally red-purple, metasoma red-purple	***Holopyga fervida* (Fabricius)** (female)
–	Body concolorous green-blue or golden greenish	**4**
4	Punctation of T2 coarse and dense, interstices smaller than puncture diameter (Fig. 49). Medial flagellomeres at most 1.5 times as long as broad (Fig. 51)	***Holopyga fervida* (Fabricius)** (male)
–	Punctation of T2 fine and sparse, interstices larger than puncture diameter at least in the middle (Fig. 50). Medial flagellomeres about 1.5 times as long as broad in the female, and about two times as long as broad in the male (Fig. 52)	***Holopyga metallica* (Dahlbom)**

##### *Holopyga fervida* group

###### Holopyga fervida

Taxon classificationAnimaliaHymenopteraChrysididae

(Fabricius, 1781)

Figs 49 , 51

Chrysis fervida Fabricius, 1781: 456.
Holopyga fervida : Abeille de Perrin 1879: 27.

####### Diagnosis.

Length 4–7 mm. The female and the male are entirely differently coloured. The female is mainly shiny red-purple, but the legs, mesopleuron, metanotum, propodeum, lower part of head and lateral corners of pronotum are blue. The male is entirely green or blue-green, sometimes with golden reflections or a completely golden metasoma. The colouration of the male is similar to *Holopyga metallica*, but the punctation of the tergites is denser and coarser (Fig. 49) and the antennal segments are shorter (Fig. 51).

####### Distribution.

Denmark. Very rare. Only two records are known from the island of Lolland (1 ♀, Bremersvold, 20.VII.1904, and 1 ♀, Røgebølle, 5.VII.1912, both leg. L. Jørgensen). – West Palearctic: Europe, northern Africa, Turkey, Iran (Kimsey and Bohart 1991, Rosa et al. 2013).

####### Biology.

Habitat: sparsely vegetated sandy areas, loess and clay banks (Kusdas 1956, Hoop 1971, Kunz 1994). Adults visit flowers of Apiaceae, Asteraceae and Euphorbiaceae (Heinrich 1964, Hoop 1971, Linsenmaier 1997, Rosa 2004). Flight period: June to August. Host: unknown.

**Figures 48–52.**
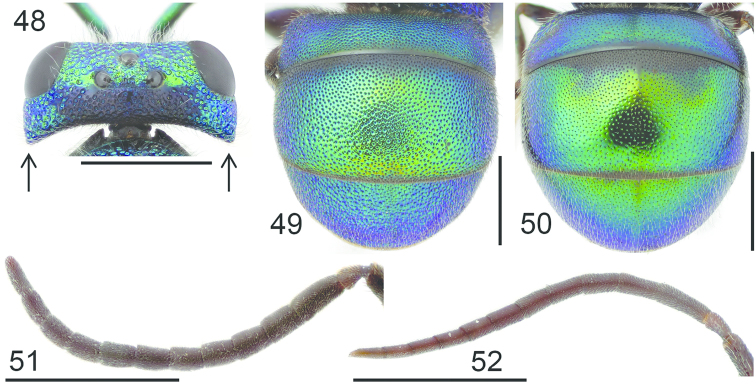
Head, dorsal view (arrows indicating temples): **48** *Holopyga generosa* ♀. Metasoma, dorsal view: **49** *Holopyga fervida* ♂ **50** *Holopyga metallica* ♂. Antenna: **51** *Holopyga fervida* ♂ **52** *Holopyga metallica* ♂. Scale 1 mm.

###### Holopyga metallica

Taxon classificationAnimaliaHymenopteraChrysididae

(Dahlbom, 1854)

Figs 50 , 52

Hedychrum metallicum Dahlbom, 1854: 68.
Holopyga curvata of authors, not (Förster, 1853).
Holopyga fervida of authors, not (Fabricius, 1781).
Holopyga metallica : Erlandsson 1971: 88.

####### Diagnosis.

Length 5–6 mm. The female is entirely blue or blue-green with golden green reflections on the pronotum, mesoscutum and metasoma. The male is golden green with blue on the metanotum and propodeum. Both sexes resemble the male of *Holopyga fervida* in colouration, but the punctation of the tergites is finer and sparser (Fig. 50), and the antennal segments are longer in the male (Fig. 52).

####### Distribution.

Finland. Rare. – West Palearctic: only known from Finland and Russian Fennoscandia (Paukkunen et al. 2014).

####### Biology.

Habitat: sparsely vegetated coastal dune areas. Most of the specimens have been collected by sweep nets from grasses and by yellow pan traps. Flight period: June to July. Host: *Dryudella stigma* (Panzer) (Crabronidae) (according to E. Valkeila’s notes).

##### *Holopyga lucida* group (former *Holopyga gloriosa* group)

###### Holopyga generosa

Taxon classificationAnimaliaHymenopteraChrysididae

(Förster, 1853)

Figs 3 , 7 , 47 , 48

Ellampus generosus Förster, 1853: 349.
Holopyga ovata Dahlbom, 1854: 51.
Holopyga amoenula of authors, not Dahlbom, 1845.
Holopyga gloriosa of authors, not (Fabricius, 1793), suppressed name (ICZN 1998).
Holopyga generosa : Linsenmaier 1987: 135.
Holopyga fastuosa ssp. generosa : Linsenmaier 1997: 57.

####### Diagnosis.

Length 7–9 mm. Both sexes are similarly bicoloured with a green or blue head and mesosoma, and a dorsally red metasoma (Fig. 47). The colouration resembles that of *Hedychrum gerstaeckeri* and the male of *Hedychrum nobile* and *Hedychrum niemelai*, but *Holopyga generosa* always has multidentate tarsal claws (Fig. 3), angular margins on the head (as in Fig. 48) and an evenly rounded margin of T3 (as in Figs 49, 50). In *Hedychrum*, the claws are bifid, the head margins are rounded and T3 usually has angular prominences laterally.

####### Distribution.

Denmark, Estonia, Finland, Latvia, Lithuania, Sweden. Common. – Trans-Palearctic: Europe, Asia Minor, northern Africa, China (Linsenmaier 1959).

####### Biology.

Habitat: sparsely vegetated sandy areas, dry meadows. Adults visit flowers of Apiaceae, Asteraceae, Euphorbiaceae, Onagraceae and Rosaceae (Molitor 1935, Linsenmaier 1997, Rosa 2004, our own obs.). Flight period: late May to late August. Host: *Astata boops* (Schranck) (Crabronidae) (Veenendaal 2012, our own obs.). Females lay their eggs in nymphs of Heteroptera before they have been captured and brought to the nest by the host (Veenendaal 2012).

###### Holopyga inflammata

Taxon classificationAnimaliaHymenopteraChrysididae

(Förster, 1853)

Ellampus inflammatus Förster, 1853: 348.
Holopyga gloriosa of authors, not (Fabricius, 1793), suppressed name (ICZN 1998).
Holopyga inflammata : Linsenmaier 1959: 34.

####### Diagnosis.

Length 5–7 mm. Both sexes have similar colouration: the head, propleuron, mesopleuron, propodeum and legs are blue or blue-violet, whereas the pronotum, mesoscutum, mesoscutellum and metascutellum are red. The colouration is relatively similar to the female of *Holopyga fervida*, but the head is completely blue (without red vertex), the metascutellum is red (not blue) and the mesoscutellum is uniformly punctured (not sparser anteriorly).

####### Distribution.

Finland, Lithuania. Very rare. In Finland, more than 30 specimens were collected in the south-eastern part of the country (Joutseno) in 1957–1960, but currently the species is classified as regionally extinct (Paukkunen 2010). In Lithuania, no records are known since 1970 (Orlovskytė et al. 2010). – West Palearctic: Europe, northern Africa, western Asia (Linsenmaier 1997, 1999).

####### Biology.

Habitat: sparsely vegetated sandy areas. Adults visit flowers of Apiaceae (Brechtel 1985, Rosa 2004). Flight period: early June to early August. Host: unknown.

###### Genus Hedychrum

Taxon classificationAnimaliaHymenopteraChrysididae

Latreille, 1802

Figs 4 , 53 , 54–65

Hedychrum Latreille, 1802: 317.

####### Note.

The genus consists of robust species with a body length ranging from 4 to 10 mm. Characteristic morphological features include the meso- and metatibial pits (Figs 56–58), the enlarged metafemur (Figs 54–55), the apically bifid tarsal claw (Fig. 4) and, in some species the apicomedial tubercle on S4 of the female (Figs 59–60). Many species show sexual dimorphism, in which the mesosoma is strikingly bicoloured in the female (Fig. 47), but uniformly green, blue or violet in the male. The hosts are crabronid wasps of the subfamily Philanthinae. About 150 species are known worldwide, the majority of which occur in the Palearctic Region and Africa (Kimsey and Bohart 1991). The European fauna consists of 17 species and several subspecies, some of which probably would deserve species rank (Rosa and Soon 2012). Five species have been found in the Nordic and Baltic countries (Paukkunen et al. 2014).

**Figure 53.**
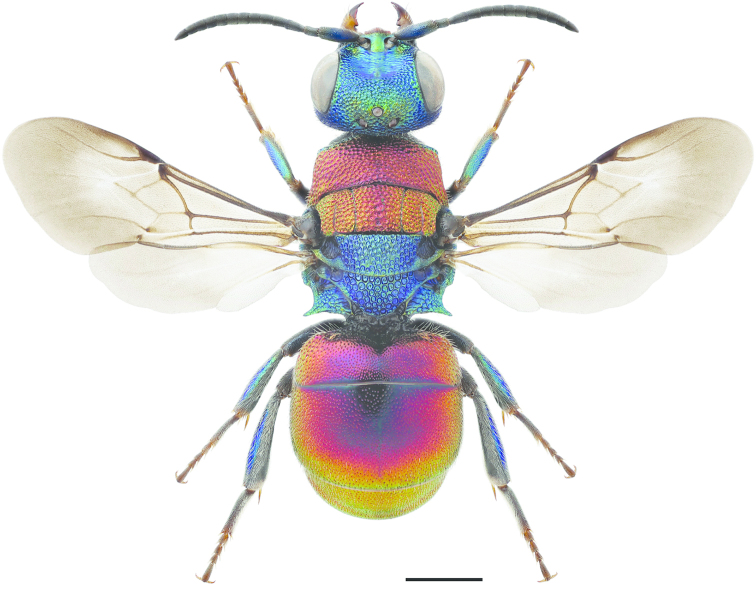
*Hedychrum niemelai* ♀. Scale 1 mm.

**Figures 54–65.**
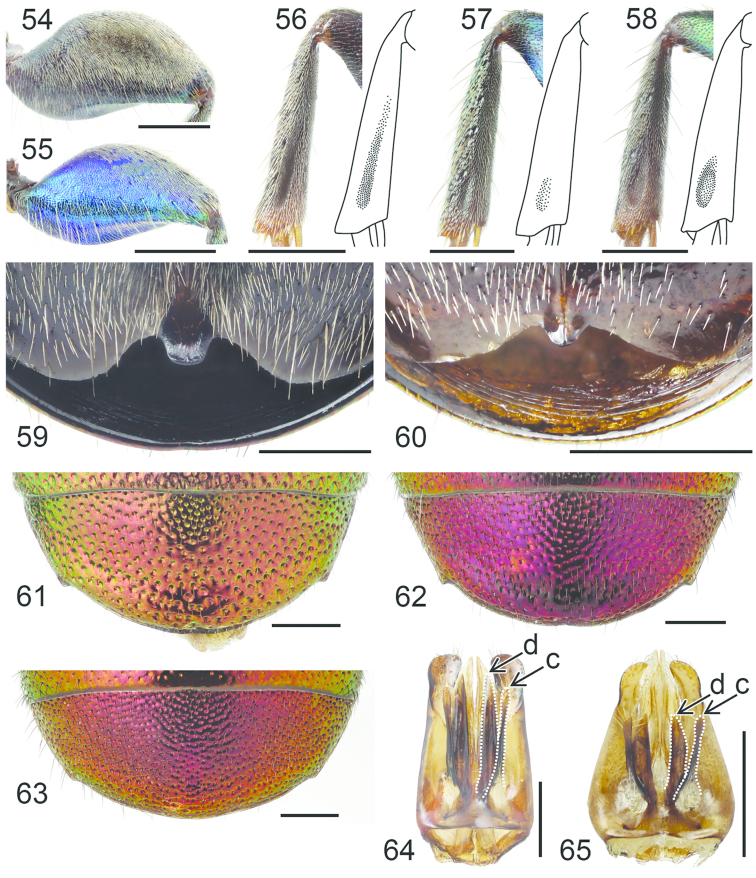
Left hindleg, ventral view: **54** *Hedychrum nobile* ♂ **55** *Hedychrum nobile* ♀. Left mesotibia: **56** *Hedychrum gerstaeckeri* ♂ **57** *Hedychrum nobile* ♂ **58** *Hedychrum niemelai* ♂. S2, ventral view: **59** *Hedychrum nobile* ♀ **60** *Hedychrum niemelai* ♀. T3, dorsal view: **61** *Hedychrum gerstaeckeri* ♂ **62** *Hedychrum nobile* ♂ **63** *Hedychrum niemelai* ♂. Genital capsule, ventral view: **64** *Hedychrum nobile* ♂ **65** *Hedychrum niemelai* ♂, d digitus, c cuspis. Scale 0.5 mm.

####### Key to *Hedychrum* species of the Nordic and Baltic countries

**Table:** 

1	Male. Anterior surface of metafemur non-metallic black or brown, covered entirely by short adpressed pubescence (Fig. 54). Head and mesosoma dorsally with long and erect pubescence, setae longer than diameter of mid-ocellus	**2**
–	Female. Anterior surface of metafemur metallic shiny, not entirely covered by short adpressed pubescence (Fig. 55). Head and mesosoma dorsally with short inclined pubescence, setae shorter than diameter of mid-ocellus	**6**
2	Head and mesosoma dorsally on pronotum, mesoscutum and mesoscutellum with coppery to greenish colour, contrasting with remaining part of the mesosoma, and light brown setae	***Hedychrum rutilans* Dahlbom**
–	Head and mesosoma completely green-bluish, with dark setae	**3**
3	Entire body green-blue	***Hedychrum chalybaeum* Dahlbom**
–	Head and mesosoma green-blue, metasoma dorsally metallic red or golden	**4**
4	Groove on inner surface of mesotibia at least half of tibial length (Fig. 56). Mandible pale brown medially. Punctation of T3 coarse (Fig. 61)	***Hedychrum gerstaeckeri* Chevrier**
–	Groove on inner surface of mesotibia shorter or indistinct (Figs 57, 58). Mandible usually not pale brown medially. Punctation of T3 finer (Figs 62, 63)	**5**
5	Groove on inner surface of mesotibia shallow and narrow, indistinctly delimited (Fig. 57). Punctation of T3 relatively sparse (Fig. 62). Digitus longer than cuspis (Fig. 64)	***Hedychrum nobile* (Scopoli)**
–	Groove on inner surface of mesotibia deep and oval, distinctly delimited (Fig. 58). (May be indistinct in small specimens.) Punctation of T3 dense (Fig. 63). Digitus not longer than cuspis (Fig. 65)	***Hedychrum niemelai* Linsenmaier**
6	S3 with apicomedial tubercle (Figs 59, 60)	**7**
–	S3 without apicomedial tubercle	**9**
7	Head dorsally, pronotum, mesoscutum and mesoscutellum anteriorly bright red	***Hedychrum chalybaeum* Dahlbom**
–	Head and mesoscutellum blue-green, pronotum and mesoscutum reddish or golden	**8**
8	Tubercle of S3 larger, apically slightly rounded, not divided (Fig. 59). Punctation of T3 sparser. Body usually larger, 6–10 mm	***Hedychrum nobile* (Scopoli)**
–	Tubercle of S3 smaller, apically divided in the middle (Fig. 60). Punctation of T3 denser. Body usually smaller, 5–8 mm	***Hedychrum niemelai* Linsenmaier**
9	Head and mesosoma dorsally with coppery red colour. Head with light brown pubescence	***Hedychrum rutilans* Dahlbom**
–	Head and mesosoma entirely blue-green or blue-violet. Head with dark brown pubescence	***Hedychrum gerstaeckeri* Chevrier**

###### Hedychrum gerstaeckeri

Taxon classificationAnimaliaHymenopteraChrysididae

Chevrier, 1869

Figs 56 , 61

Hedychrum Gerstaeckeri Chevrier, 1869: 47.

####### Diagnosis.

Length 4–8 mm. The female differs from the females of other *Hedychrum* species by having a completely blue, violet-blue or green-blue mesosoma. As in *Hedychrum rutilans*, the female does not have an apicomedial tubercle on S3. Both sexes also have medially pale brown or yellowish mandibles. The male is similar to the female in colouration and can be confused with the males of *Hedychrum niemelai* and *Hedychrum nobile*. However, the punctation of T3 is coarser in *Hedychrum gerstaeckeri* (Fig. 61) and the mesotibial groove is longer and deeper (Fig. 56).

####### Distribution.

Denmark, Estonia, Finland, Latvia, Lithuania. Relatively common. – Trans-Palearctic: from western Europe to Japan, China and Taiwan (Rosa et al. 2014).

####### Biology.

Habitat: sparsely vegetated sandy areas, dry meadows. Adults often visit flowers of Apiaceae, Asteraceae and Euphorbiaceae (Trautmann 1927, Kusdas 1956, Kunz 1994, Linsenmaier 1997, Westrich 1979, Rosa 2004, our own obs.). Flight period: mid-June to late August. Host: *Cerceris rybyensis* (Linnaeus) and *Cerceris ruficornis* (Fabricius) (Crabronidae) (Berland and Bernard 1938, Grandi 1961, Petit 1975, Westrich 1979, Brechtel 1985, Gayubo et al. 1987, Saure 1998).

###### Hedychrum rutilans

Taxon classificationAnimaliaHymenopteraChrysididae

Dahlbom, 1854

Hedychrum rutilans Dahlbom, 1854: 76.
Hedychrum intermedium of authors, not Dahlbom, 1845.

####### Diagnosis.

Length 4–10 mm. The species is usually easy to differentiate from other *Hedychrum* species by the coppery red colour on the head dorsum, pronotum, mesoscutum and mesoscutellum. Also the pubescence is paler brown than in other species. The ventral part of the head, metanotum, propodeum, mesopleuron and legs are contrastingly blue or blue-green. Sometimes the coppery red colour of the head and/or mesosoma is partially replaced by golden green or blue colour, especially in the male. The mesotibia of the male has a shallow depression on its inner surface, reaching half of the tibial length. The female does not have an apicomedial tubercle on S3.

####### Distribution.

Denmark, Estonia, Finland, Latvia, Lithuania. Relatively common. – Trans-Palearctic: Europe, northern Africa, Turkey, southwestern Russia, Siberia (Linsenmaier 1959, 1997, Kimsey and Bohart 1991).

####### Biology.

Habitat: sparsely vegetated sandy areas, dry meadows. Adults often visit flowers of Apiaceae and Asteraceae (Kusdas 1956, Kunz 1994, Rosa 2004, our own obs.). Flight period: early July to late August. Host: *Philanthus triangulum* (Fabricius) (Crabronidae) (Ferton 1910, Trautmann 1927, Morgan 1984, Veenendaal 1987). The female does not always enter the host nest for ovipositing, but may oviposit on the prey (*Apis mellifera* Linnaeus) while it is being transported to the nest by the host (Veenendaal 1987, Baumgarten 1995).

###### Hedychrum nobile

Taxon classificationAnimaliaHymenopteraChrysididae

(Scopoli, 1763)

Figs 4 , 54 , 55 , 57 , 59 , 62 , 64

Sphex nobilis Scopoli, 1763: 297.
Chrysis lucidula Fabricius, 1775: 358.
Chrysis regia Fabricius, 1793: 243.
Hedychrum nobile : Mocsáry 1889: 172.

####### Diagnosis.

Length 6–10 mm. The male and female are differently coloured. In the male, the head and mesosoma are completely green-blue and the metasoma is golden red (rarely greenish golden). In the female, the pronotum and mesoscutum are bright red (as in Fig. 53) or golden yellow, whereas the rest of the body has similar colouration as in the male. The pubescence is dark brown in both sexes. The species is easily confused with *Hedychrum niemelai*, but the mesotibial groove of the male is shallower and narrower, often indistinct (Fig. 57), and the female has a broader, apically undivided, tubercle on S3 (Fig. 59). Punctation of T3 is also sparser in both sexes, especially in the male (Fig. 62).

####### Distribution.

Denmark, Estonia, Finland, Latvia, Lithuania, Norway, Sweden. Very common. – Trans-Palearctic: from Europe to Siberia (Linsenmaier 1959).

####### Biology.

Habitat: sparsely vegetated sandy areas, dunes. Adults are often found on flowers of Apiaceae, Asteraceae, Euphorbiaceae, Onagraceae and Rosaceae (Kusdas 1956, Brechtel 1985, Rosa 2004, our own obs.). Flight period: June to August. Host: *Cerceris arenaria* (Linnaeus) (Crabronidae) (Alfken 1915, Lomholdt 1975, Petit 1975, Schmid-Egger et al. 1995, Saure 1998, our own obs.), possibly also *Cerceris quadrifasciata* (Panzer) and *Cerceris rybyensis* (Linnaeus) (Alfken 1915, Lomholdt 1975).

###### Hedychrum niemelai

Taxon classificationAnimaliaHymenopteraChrysididae

Linsenmaier, 1959

Figs 53 , 58 , 60 , 63 , 65

Hedychrum aureicolle ssp. *niemeläi* Linsenmaier, 1959: 38.
Hedychrum niemelai : Morgan 1984: 8.

####### Diagnosis.

Length 5–8 mm. The colouration is similar to *Hedychrum nobile*, but the pronotum and mesoscutum of the female are usually bright red (Fig. 53) and rarely yellowish. The mesotibial depression of the male is deeper than in *Hedychrum nobile* and oval or longitudinal in shape (Fig. 58). The tubercle on the posterior margin of S3 of the female is apically divided and smaller (Fig. 60) than in *Hedychrum nobile*. Additionally, the punctation of T3 is denser, especially in the male (Fig. 63).

####### Distribution.

Denmark, Estonia, Finland, Latvia, Lithuania, Norway, Sweden. Common. – Trans-Palearctic: from Europe to China (Heilongjiang) (Rosa et al. 2014).

####### Biology.

Habitat: sparsely vegetated sandy areas. Adults are often found on flowers of Apiaceae, Asteraceae and Onagraceae (Rosa 2004, our own obs.). Flight period: from early June to late August. Host: *Cerceris quadrifasciata* (Panzer) and *Cerceris quinquefasciata* (Rossi) (Crabronidae) (Schmid-Egger et al. 1995, Saure 1998, our own obs.). Possibly also *Cerceris arenaria* (Linnaeus), *Cerceris ruficornis* (Fabricius) and *Cerceris rybyensis* (Linnaeus) (Lomholdt 1975, Morgan 1984).

###### Hedychrum chalybaeum

Taxon classificationAnimaliaHymenopteraChrysididae

Dahlbom, 1854

Hedychrum chalybaeum Dahlbom, 1854: 64.

####### Diagnosis.

Length 4–6 mm. The male is easy to differentiate from other *Hedychrum* species by its entirely green-blue body. Therefore it superficially resembles *Holopyga metallica* and the male of *Holopyga fervida*. The female is completely differently coloured: the vertex, pronotum, mesoscutum, mesoscutellum and dorsum of the metasoma are bright red, whereas the ventral and lateral parts of the head and mesosoma, including the legs, are blue or greenish. The pubescence is dark brown and the apicomedial tubercle on S3 of the female is very small.

####### Distribution.

Latvia, Lithuania. Very rare. The species has been recorded in one locality in Latvia (Tumšs 1976) and in three localities in Lithuania (Wengris 1962). – Trans-Palearctic: from western Europe to Russian Far East, Mongolia and China (Rosa et al. 2014).

####### Biology.

Habitat: sparsely vegetated sand and loess areas (Kunz 1994). Adults visit flowers of Apiaceae and Asteraceae (Heinrich 1964). Flight period: July to August. Host: *Cerceris interrupta* (Panzer) (Crabronidae) (Schmid-Egger 2000). Host records implicating *Bembecinus tridens* (Fabricius) (Crabronidae: Bembicinae) are probably erroneous, as supporting evidence is lacking.

###### Genus Hedychridium

Taxon classificationAnimaliaHymenopteraChrysididae

Abeille de Perrin, 1878

Figs 5 , 8 , 66 , 67–74

Hedychridium Abeille de Perrin, 1878: 3.
Euchrum Semenov, 1954: 103.

####### Note.

This genus comprises a heterogeneous group of small colourful species ranging from 2 to 7 mm in length. Characteristic morphological features include the single perpendicular tooth of the tarsal claw and the transverse pronotal carina (Kimsey and Bohart 1991). The posterior margin of T3 is evenly rounded, without any angular projections. The biology of most species is poorly known, but according to published records, the larvae develop as nest parasites of ground-nesting crabronid wasps and solitary bees. *Hedychridium* is the second largest genus of Chrysididae and includes more than 300 recognised species worldwide. The highest diversity is found in arid parts of the Holarctic Region and southern Africa. A total of 86 species are known from Europe (Rosa and Soon 2012). Of these, seven occur in the Nordic and Baltic countries (Paukkunen et al. 2014). The genus is here divided into three species-groups according to Linsenmaier (1968, 1997).

**Figure 66.**
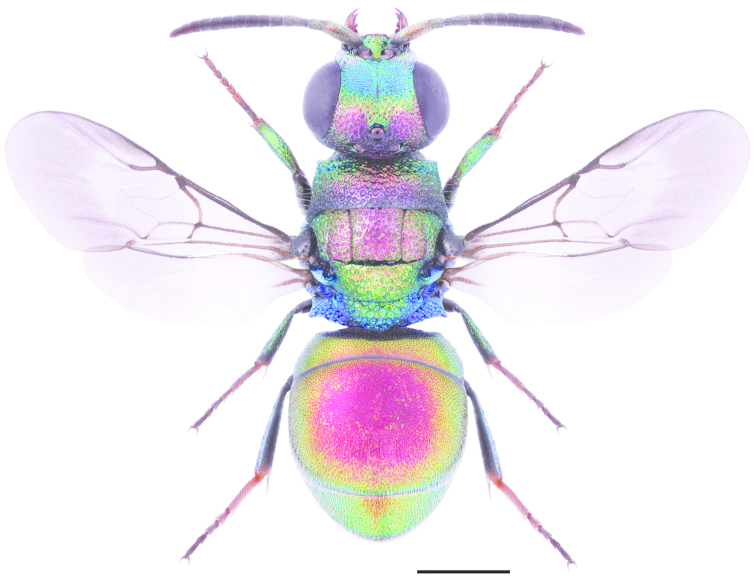
*Hedychridium ardens* ♀. Scale 1 mm.

**Figures 67–74.**
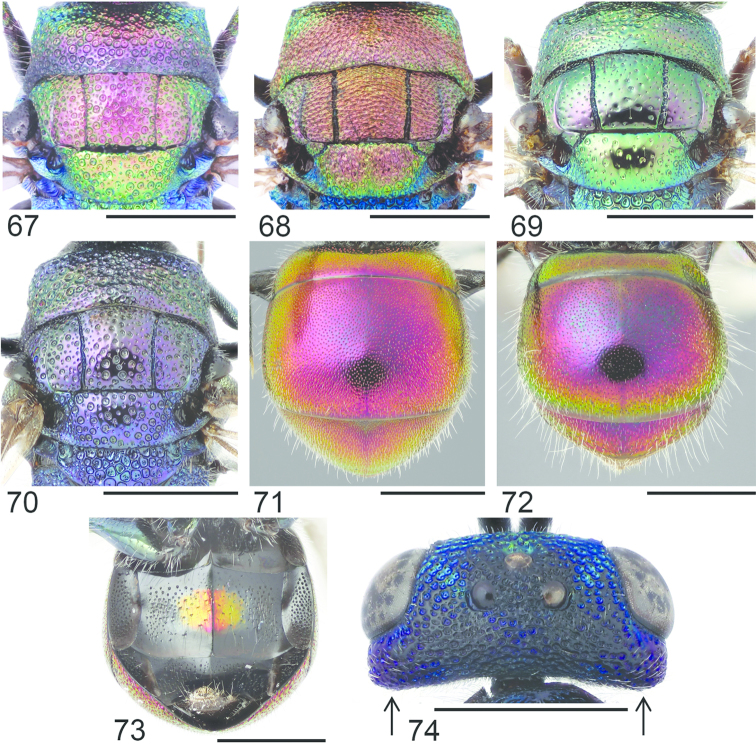
Pronotum, mesoscutum and mesoscutellum, dorsal view: **67** *Hedychridium ardens* ♀, **68** *Hedychridium coriaceum* ♀ **69** *Hedychridium cupreum* ♀ **70** *Hedychridium purpurascens* ♀. Metasoma, dorsal view: **71** *Hedychridium cupreum* ♀ **72** *Hedychridium purpurascens* ♀. Metasoma, ventral view: **73** *Hedychridium purpurascens* ♀. Head, dorsal view (arrows indicating temples): **74** *Hedychridium roseum* ♀. Scale 1 mm.

####### Key to *Hedychridium* species of the Nordic and Baltic countries

**Table:** 

1	Body entirely green or blue-green. Very small species, body length 2–4 mm	***Hedychridium zelleri* (Dahlbom)**
–	Body partially red, golden red or orange. Mostly larger species	**2**
2	Metasoma non-metallic red or orange, sometimes with weak purple reflections	**3**
–	Metasoma with strong metallic shine	**4**
3	Head and pronotum dorsally with coppery red colour. T3 posteriorly with coarse punctures in the male	***Hedychridium caputaureum* Trautmann & Trautmann**
–	Head and pronotum entirely blue-green or dark blue. T3 posteriorly with fine punctures in the male	***Hedychridium roseum* (Rossi)**
4	Punctation of mesoscutum and mesoscutellum sparse, punctures separated with broad smooth interstices (Figs 69, 70). Metasoma red-purple with blue-green reflections (Figs 71, 72)	**5**
–	Punctation of mesoscutum and mesoscutellum denser, punctures not separated with broad smooth interstices (Figs 67, 68). Metasoma bright red or golden red with greenish reflections (Fig. 66)	**6**
5	S2 medially with greenish-golden metallic spot (Fig. 73). Pubescence of body relatively short. Setae on metasoma shorter than the third antennal segment (Fig. 72). Punctation of mesoscutum and mesoscutellum relatively dense (Fig. 70). Scapal basin only medially with fine horizontal ridging	***Hedychridium purpurascens* (Dahlbom)**
–	S2 without metallic spot (occasionally with slight metallic sheen). Body with long, erect, whitish pubescence. Setae on metasoma as long as or longer than the third antennal segment (Fig. 71). Punctation of mesoscutum and mesoscutellum sparse (Fig. 69). Scapal basin covered broadly with fine cross-ridging	***Hedychridium cupreum* (Dahlbom)**
6	Pronotum and mesoscutum dull with dense coriaceous punctation (Fig. 68). Mesoscutellum with dense punctation and distinct rugae (Fig. 68). Length of malar space less than basal width of mandible	***Hedychridium coriaceum* (Dahlbom)**
–	Pronotum and mesoscutum shiny with sparser punctation (Fig. 67). Mesoscutellum with shiny interstices between punctures, without distinct rugae (Fig. 67). Length of malar space equal to basal width of mandible	***Hedychridium ardens* (Coquebert)**

##### *Hedychridium monochroum* group

###### Hedychridium zelleri

Taxon classificationAnimaliaHymenopteraChrysididae

(Dahlbom, 1845)

Hedychrum Dahlbom, 1845: 2.
Hedychridium zelleri : du Buysson (in André) 1892: 183.

####### Diagnosis.

Length 2–4 mm. The species differs from other species of *Hedychridium* by its smaller size and almost completely green or bluish body, which occasionally has weak golden reflections dorsally on the mesosoma. The tarsi are pale brown. Exceptionally small and greenish males of *Hedychridium ardens* can resemble *Hedychridium zelleri*, but they have sparser and coarser punctation dorsally on the mesosoma.

####### Distribution.

Finland. Rare. – West Palearctic: northern and central Europe (Linsenmaier 1959).

####### Biology.

Habitat: sparsely vegetated sandy areas, usually near seashore. Adults are occasionally found on flowers. Flight period: late June to early August. Host: species of *Miscophus* Jurine (Crabronidae) (Müller 1918, Saure 1998, our own obs.). In central Europe, possibly also *Diodontus tristis* (Vander Linden) and *Diodontus minutus* (Fabricius) (Jacobs and Kornmilch 2007).

##### *Hedychridium ardens* group

###### Hedychridium ardens

Taxon classificationAnimaliaHymenopteraChrysididae

(Coquebert, 1801)

Figs 8 , 66 , 67

Chrysis ardens Coquebert, 1801: 59.
Hedychrum minutum Lepeletier, 1806: 122.
Chrysis integra Dahlbom, 1829: 17, not Fabricius, 1787.
Hedychridium ardens : Frey-Gessner 1887: 40.

####### Diagnosis.

Length 3–5 mm. Both sexes have coppery red colour dorsally on the head, pronotum, mesoscutum, mesoscutellum and metasoma, whereas the frons, anterior corners of pronotum, metanotum, tibiae and apex of the metasoma are mainly greenish (Fig. 66). The propodeum and the mesopleuron are usually blue. The punctation of the mesoscutum (Fig. 67) is denser than in *Hedychridium cupreum* and *Hedychridium purpurascens*, but not as dense as in *Hedychridium coriaceum*. Small males are sometimes greenish all over with only weak coppery reflections dorsally, and can be confused with *Hedychridium zelleri*. The punctation of the mesoscutum is however sparser and coarser than in *Hedychridium zelleri*.

####### Distribution.

Denmark, Estonia, Finland, Latvia, Lithuania, Norway, Sweden. Common. – Trans-Palearctic: Europe, Mongolia, Russian Far East (Kimsey and Bohart 1991, Linsenmaier 1997, Kurzenko and Lelej 2007).

####### Biology.

Habitat: sparsely vegetated sandy areas, dunes, dry meadows. Adults visit flowers of Apiaceae, Asteraceae, Crassulaceae, Euphorbiaceae and Rosaceae (Molitor 1935, our own obs.). Flight period: late May to late August. Host: *Diodontus tristis* (Vander Linden), *Oxybelus bipunctatus* Olivier, *Tachysphex nitidus* (Spinola), *Tachysphex obscuripennis* (Schenck) and *Tachysphex pompiliformis* (Panzer) (Crabronidae) (Trautmann 1927, Berland and Bernard 1938, Benno 1950, Else 1973, Kofler 1975, Morgan 1984, van der Smissen 2001).

###### Hedychridium coriaceum

Taxon classificationAnimaliaHymenopteraChrysididae

(Dahlbom, 1854)

Fig. 68

Hedychrum coriaceum Dahlbom, 1854: 88.
Hedychridium coriaceum : du Buysson (in André) 1892: 195.

####### Diagnosis.

Length 3–5 mm. The species is characterised by the very dense and fine punctation of the pronotum and mesoscutum, whereby the surface appears completely dull (Fig. 68). The mesoscutellum also has dense punctation and rugae between the punctures (Fig. 68). In *Hedychridium ardens*, *Hedychridium cupreum* and *Hedychridium purpurascens* the punctation is sparser and the surface shinier. The colour of the vertex, pronotum, mesoscutum and mesoscutellum is brownish red (Fig. 68). The frons, anterior corners of pronotum, metanotum, propodeum, mesopleuron and tibiae are mainly green or blue. The metasoma is coppery red or sometimes greenish.

####### Distribution.

Denmark, Estonia, Finland, Latvia, Lithuania, Sweden. Relatively rare. – Trans-Palearctic: from Europe and northern Africa to China (Linsenmaier 1959, Rosa et al. 2014). (In northern Africa represented by ssp. *jendoubense* Linsenmaier, 1987).

####### Biology.

Habitat: sparsely vegetated sandy areas. Adults visit flowers of Asteraceae, Euphorbiaceae and Rosaceae (Heinrich 1964, Hoop 1971, Linsenmaier 1997, Rosa 2004, our own obs.). Flight period: early June to late August. Host: *Lindenius albilabris* (Fabricius) (Crabronidae) (Arnold 1908, 1910, Mortimer 1913, Morgan 1984, Tischendorf 1998) and possibly also *Oxybelus uniglumis* (Linnaeus) (Alfken 1915).

###### Hedychridium cupreum

Taxon classificationAnimaliaHymenopteraChrysididae

(Dahlbom, 1845)

Figs 69 , 71

Hedychrum cupreum Dahlbom, 1845: 3.
Hedychrum integrum Dahlbom, 1854: 86, not (Dahlbom, 1829).
Hedychridium cupreum : Abeille de Perrin 1879: 39.

####### Diagnosis.

Length 4–5 mm. The species differs from other species of the genus by having very sparse punctation and smooth interstices between punctures on the mesoscutum and mesoscutellum (Fig. 69). The head and mesosoma are dorsally mainly coppery red or greenish (Fig. 69), whereas the metasoma is dorsally red-purple with blue-green reflections. The frons, anterior corners of pronotum, metanotum, propodeum, mesopleuron and tibiae are mainly green or blue. Compared to *Hedychridium purpurascens*, the metasomal pubescence is longer (Fig. 71) and the scapal basin has broader cross-ridging. S2 does not have a clearly delimited metallic spot medially.

####### Distribution.

Denmark, Estonia, Finland, Latvia, Lithuania, Norway, Sweden. Relatively common. – Trans-Palearctic: from western Europe to Japan, Mongolia and China (Linsenmaier 1959, Kurzenko and Lelej 2007, Rosa et al. 2014).

####### Biology.

Habitat: sparsely vegetated sandy areas. Adults occasionally visit flowers of Asteraceae and Caryophyllaceae (our own obs.). Flight period: from early June to late August. Host: primarily *Dryudella pinguis* (Dahlbom) (Else 1973, Schmid-Egger et al. 1995, Saure et al. 1998), but possibly also *Dryudella stigma* (Panzer), *Harpactus lunatus* (Dahlbom) and *Harpactus tumidus* (Panzer) (Crabronidae) (Trautmann and Trautmann 1919, Lefeber 1976, Jacobs and Kornmilch 2007).

###### Hedychridium purpurascens

Taxon classificationAnimaliaHymenopteraChrysididae

(Dahlbom, 1854)

Figs 70 , 72 , 73

Hedychrum purpurascens Dahlbom, 1854: 85.
Hedychridium purpurascens : du Buysson (in André) 1892: 208.

####### Diagnosis.

Length 5–6 mm. The species closely resembles *Hedychridium cupreum*, but the colouration of the head and mesosoma are dorsally darker violet (Fig. 70), sometimes nearly black. The punctation of the mesoscutum and mesoscutellum is denser (Fig. 70), the pubescence of the metasoma shorter (Fig. 72) and the fine cross-ridging of the scapal basin is restricted to a smaller area medially. S2 has a round metallic spot medially (Fig. 73).

####### Distribution.

Estonia. Very rare. Nine specimens were collected in 2013 from Kauksi, northern shore of Lake Peipus. No other records are known from the Nordic and Baltic countries. – West Palearctic: central Europe (Linsenmaier 1959).

####### Biology.

Habitat: sparsely vegetated sandy areas. Adults often sit on roots of trees and bask in the sun (Trautmann 1927). Flight period: July to August. Host: unknown.

##### *Hedychridium roseum* group

###### Hedychridium caputaureum

Taxon classificationAnimaliaHymenopteraChrysididae

Trautmann & Trautmann, 1919

Hedychridium roseum var. caputaureum Trautmann & Trautmann, 1919: 35.
Hedychridium chloropygum ssp. spatium Linsenmaier, 1959: 59.
Hedychridium caputaureum : Niehuis 2001: 121.
Hedychridium chloropygum of authors, not du Buysson, 1888.

####### Diagnosis.

Length 5–7 mm. Together with *Hedychridium roseum* this species is easily differentiated from other species of the genus by its non-metallic red or orange metasoma. The head and mesosoma are mainly green or blue, but as opposed to *Hedychridium roseum*, the vertex, pronotum and lateral fields of the mesoscutum have coppery red colour or reflections. This coppery colour is sometimes only weakly visible. The metasoma often has weak purple reflections posteriorly. Especially in the males, the punctation of T3 and T2 is usually coarser compared to *Hedychridium roseum*.

####### Distribution.

Estonia, Finland, Latvia, Lithuania, Sweden. Rare. – West Palearctic: Europe and western Asia (Linsenmaier 1997).

####### Biology.

Habitat: sparsely vegetated sandy areas, dry meadows. Adults visit flowers of Apiaceae (Kusdas and Turner 1955). Flight period: mid-June to late August. Host: *Astata minor* (Kohl) (Crabronidae) (Linsenmaier 1968, Saure 1998, our own obs.).

####### Remarks.

*Hedychridium caputaureum* was recently considered to be a central and northern European subspecies of *Hedychridium chloropygum* (Arens 2010). However, as these taxa are both well characterised and have partially overlapping distribution areas, we treat them as separate species following Paukkunen et al. (2014).

###### Hedychridium roseum

Taxon classificationAnimaliaHymenopteraChrysididae

(Rossi, 1790)

Figs 5 , 74

Chrysis carnea var. rosea Rossi, 1790: 75.
Chrysis rufa Panzer, 1800: 16.
Chrysis Dahlbom, 1829: 13.
Hedychridium roseum : Abeille de Perrin 1879: 35.

####### Diagnosis.

Length 5–8 mm. The species differs from *Hedychridium caputaureum* by having the vertex (Fig. 74) and mesosoma completely blue or greenish, sometimes nearly black, without any trace of coppery red colour. Some males, however, have golden reflections on the pronotum and mesoscutum. The punctation of the tergites is finer and denser throughout, not becoming coarser posteriorly on T2 and T3 as in most males of *Hedychridium caputaureum*. The metasoma is similarly non-metallic red or orange as in *Hedychridium caputaureum*, and may have weak metallic violet reflections posteriorly.

####### Distribution.

Denmark, Estonia, Finland, Latvia, Lithuania, Norway, Sweden. Common. – Trans-Palearctic: from western Europe to Siberia, China and Russian Far East (Linsenmaier 1959, Kurzenko and Lelej 2007, Rosa et al. 2014).

####### Biology.

Habitat: sparsely vegetated sandy areas, dry meadows. Adults visit flowers of Apiaceae, Asteraceae, Crassulaceae and Euphorbiaceae (Trautmann 1927, Kusdas 1956, Heinrich 1964, Brechtel 1985, Linsenmaier 1997, Rosa 2004). Flight period: late June to late August. Host: primarily *Astata boops* (Schranck) (Crabronidae) (Reder 2010, our own obs.), but possibly also *Dryudella stigma* (Panzer), *Tachysphex pompiliformis* (Panzer) and *Harpactus tumidus* (Panzer) (Crabronidae) (Shuckard 1837, Müller 1918, Forsius 1925, Doronin 1996, Saure 1998).

#### Tribe Chrysidini

Members of this tribe are characterised by the simple untoothed tarsal claw, the transverse subapical pit row on T3, and the transverse preoccipital welt or carina (Kimsey and Bohart 1991). With more than 1.500 species and 30 genera, Chrysidini is the largest tribe of Chrysididae. A total of 11 genera are known from Europe (Rosa and Soon 2012), five of which are found in the Nordic and Baltic countries (Paukkunen et al. 2014).

##### Genus Pseudospinolia

Taxon classificationAnimaliaHymenopteraChrysididae

Linsenmaier, 1951

Figs 9 , 75

Pseudospinolia Linsenmaier, 1951: 31.

###### Note.

This taxon has been treated as a subgenus of *Euchroeus* Latreille or *Spinolia* Dahlbom by some authors (Linsenmaier 1959, Morgan 1984, Arens 2014). We follow the classification of Kimsey and Bohart (1991) and regard it as a distinct genus. Characteristic morphological features include the broadly open marginal cell (Fig. 9), the edentate T3, the deep and subdivided depression laterally on the pronotum, and the elongate transverse frontal carina connecting the compound eyes. Members of the genus parasitise solitary vespids of the subfamily Eumeninae (e.g. *Odynerus* Latreille). Approximately 15 species are recognised worldwide, most of which occur in the Palearctic Region, particularly in the Middle East and the Mediterranean region (Kimsey and Bohart 1991). The European fauna consists of eight species (Rosa and Soon 2012), and one, *Pseudospinolia neglecta*, is found in the Nordic and Baltic countries (Paukkunen et al. 2014).

**Figure 75.**
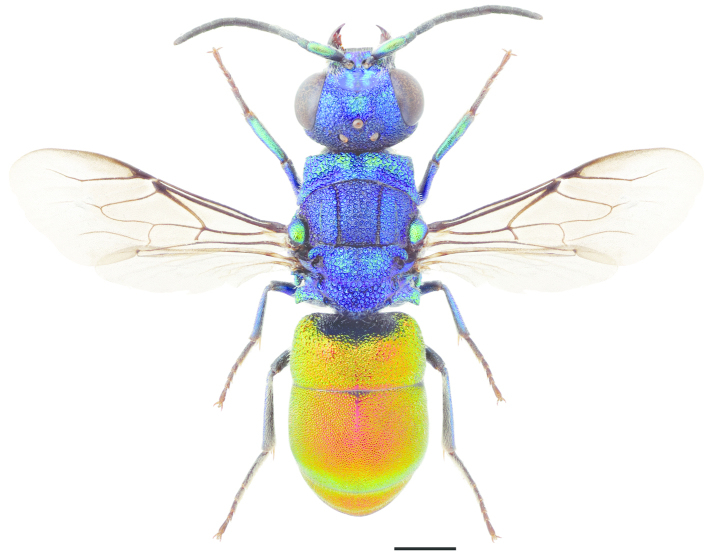
*Pseudospinolia neglecta* ♀. Scale 1 mm.

##### Pseudospinolia neglecta

Taxon classificationAnimaliaHymenopteraChrysididae

(Shuckard, 1837)

Figs 9 , 75

Chrysis austriaca Dahlbom, 1829: 14, not Fabricius, 1804.
Chrysis neglecta Shuckard, 1837: 169.
Chrysis integrella Dahlbom, 1854: 133.
Pseudospinolia neglecta : Kimsey and Bohart 1991: 548.

###### Diagnosis.

Length 5–9 mm. Both sexes are bicoloured with a green or blue head and mesosoma, and a golden red metasoma (Fig. 75). The vertex and the anterior margin of the pronotum often have golden reflections. The metasoma is very finely and densely punctured on the tergites, causing the surface to appear dull (Fig. 75). The posterior margin of T3 is edentate. The species can be confused with similarly coloured species of *Chrysura*, but the radial sector vein of the forewing does not reach the wing margin (Fig. 10).

###### Distribution.

Denmark, Estonia, Finland, Latvia, Lithuania, Norway, Sweden. Relatively rare. – Trans-Palearctic/Holarctic? Europe, Asia, Russian Far East, China, USA, Canada (Kimsey and Bohart 1991, Kurzenko and Lelej 2007, Rosa et al. 2014). Possibly accidentally introduced to North America (Bohart and Kimsey 1982).

###### Biology.

Habitat: sparsely vegetated areas with clay or sandy soil, gardens with clay structures, such as barn walls. Adults occasionally visit flowers of Asteraceae, Crassulaceae and Rosaceae (Kusdas 1956, Heinrich 1964, Ressl 1966, Linsenmaier 1997, Rosa 2004, 2006). Flight period: late May to mid-August. Host: *Odynerus spinipes* (Linnaeus) and *Odynerus reniformis* (Gmelin) (Vespidae) (Smith 1862, Adlerz 1910, Trautmann 1927, Linsenmaier 1959, Banaszak 1980, Morgan 1984), possibly also *Ancistrocerus parietum* (Linnaeus) and *Gymnomerus laevipes* (Shuckard) (Dahlbom 1854, Berland and Bernard 1938). Host records mentioning bees of the genera *Osmia* Panzer and *Heriades* Spinola (Megachilidae) are doubtful, as noted by Kunz (1994).

##### Genus Spinolia

Taxon classificationAnimaliaHymenopteraChrysididae

Dahlbom, 1854

Fig. 76

Spinolia Dahlbom, 1854: 363.
Achrysis Semenov, 1892: 486.

###### Note.

Members of this genus are characterised by the broadly open marginal cell, the U-shaped projection on the lower mesopleuron, the dentate segments of the ovipositor, and the frons with two rounded, flattened and usually striate areas particularly in the male (Kimsey and Bohart 1991). The medial cell of the forewing is asetose. The larvae are nest parasites of solitary vespids of the subfamily Eumeninae (e.g. *Pterocheilus* Klug and *Hemipterochilus* Ferton). The genus consists of 15 Palearctic species, most of which are found in northern Africa, Middle East and central Asia. In Europe, seven species are known (Rosa and Soon 2012), of which one, *Spinolia unicolor*, is recorded from the Nordic and Baltic countries (Paukkunen et al. 2014).

**Figure 76.**
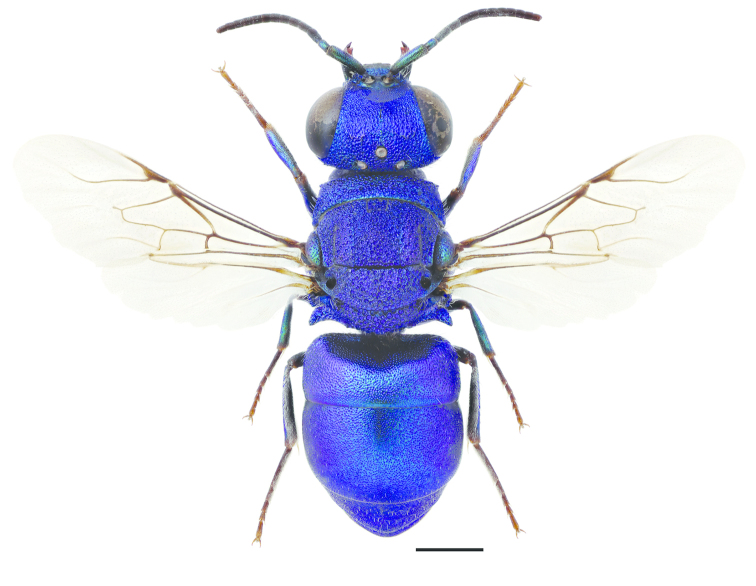
*Spinolia unicolor* ♀. Scale 1 mm.

##### Spinolia unicolor

Taxon classificationAnimaliaHymenopteraChrysididae

(Dahlbom, 1831)

Figs 76

Chrysis unicolor Dahlbom, 1831: 32.
Spinolia unicolor : du Buysson (in André) 1893: 244.

###### Diagnosis.

Length 4–6 mm. The species is characterised by the entirely blue, greenish or violet-blue body, and the forewing radial sector vein, which ends remote from the wing margin (Fig. 76). The T3 is posteriorly edentate and has a small tooth anteriorly.

###### Distribution.

Denmark, Latvia, Sweden. Very rare. – Trans-Palearctic: from western Europe to Mongolia (Linsenmaier 1959).

###### Biology.

Habitat: xerothermic sparsely vegetated sandy areas, often close to the seashore. Adults occasionally visit flowers of Asteraceae, Lamiaceae and Rosaceae (Trautmann 1927, Benno 1950, Linsenmaier 1959). Flight period: late June to mid-August. Host: *Pterocheilus phaleratus* (Panzer) (Vespidae: Eumeninae) (Erlandsson 1968, Sörensson and Cederberg 2010).

##### Genus Chrysis

Taxon classificationAnimaliaHymenopteraChrysididae

Linnaeus, 1761

Figs 6 , 10 , 77–92 , 93–101 , 102–109 , 110–125 , 126–137 , 138–144 , 145–149 , 150–164 , 165–178 , 179 , 180–186 , 187–195

Chrysis Linnaeus, 1761: 414.
Chrysogona Förster, 1853: 327.
Tetrachrysis Lichtenstein, 1876: 27.
Hexachrysis Lichtenstein, 1876: 27.

###### Note.

With more than a thousand currently recognised species, *Chrysis* is the largest and most heterogeneous genus of Chrysididae. It is best defined by a combination of several variable and non-unique characters, such as the closed or nearly closed forewing marginal cell, the usually four- or six-toothed posterior margin of T3, and the usually distinct transverse frontal carina on the frons. Members of the genus parasitise a wide range of solitary wasps and bees in the families Vespidae, Sphecidae, Crabronidae, Megachilidae and Apidae. They are found worldwide, but the vast majority of species is found in the Holarctic and Afrotropical Regions. The European fauna consists of nearly 190 species and numerous subspecies (Rosa and Soon 2012). Up to now, 35 species have been found in the Nordic and Baltic countries (Paukkunen et al. 2014). The genus was first formally divided into species-groups by Linsenmaier (1959). Our classification of the species-groups follows Kimsey and Bohart (1991).

**Figures 77–92.**
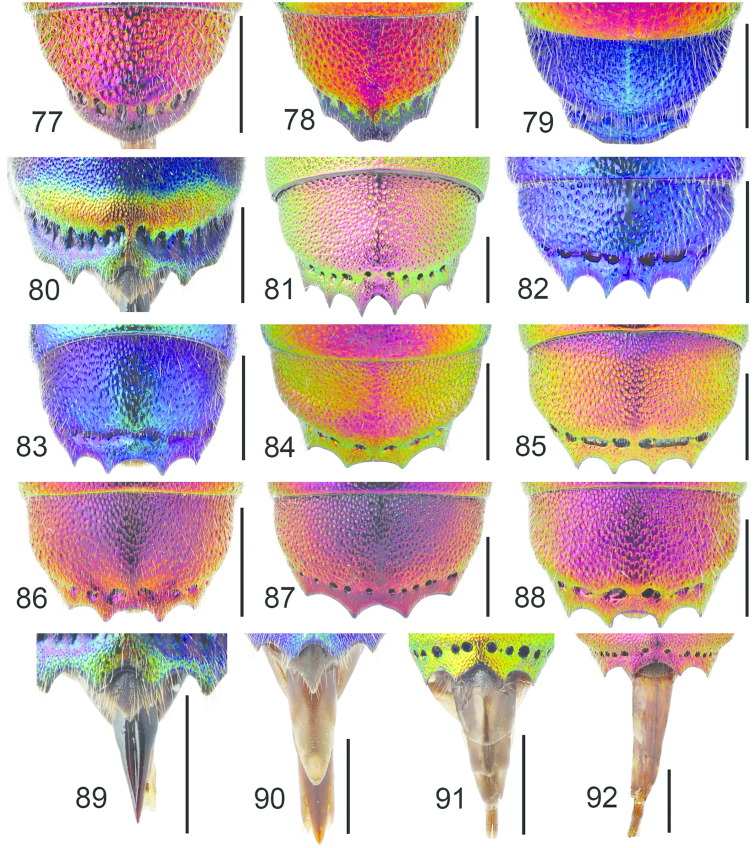
T3, dorsal view: **77** *Chrysis succincta* ♀ **78** *Chrysis illigeri* ♀ **79** *Chrysis viridula* ♀ **80** *Chrysis equestris* ♀ **81** *Chrysis sexdentata* ♀ **82** *Chrysis indigotea* ♂ **83** *Chrysis iris* ♂ **84** *Chrysis impressa* ♀ **85** *Chrysis impressa* ♂ **86** *Chrysis angustula* ♂ **87** *Chrysis subcoriacea* ♂ **88** *Chrysis longula* ♂. Ovipositor, dorsal view: **89** *Chrysis equestris* ♀ **90** *Chrysis zetterstedti* ♀ **91** *Chrysis solida* ♀ **92** *Chrysis impressa* ♀. Scale 1 mm.

**Figures 93–101.**
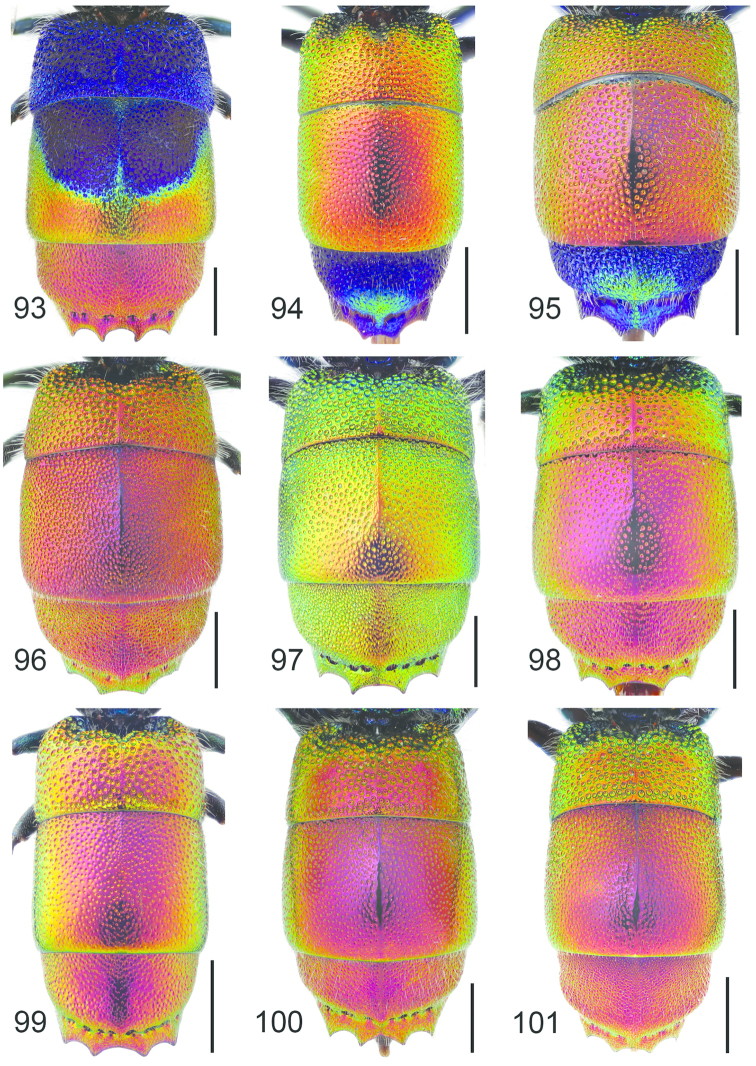
Metasoma, dorsal view: **93** *Chrysis fulgida* ♂ **94** *Chrysis rutilans* ♀ **95** *Chrysis splendidula* ♀ **96** *Chrysis ruddii* ♀ **97** *Chrysis vanlithi* ♀ **98** *Chrysis clarinicollis* ♀ **99** *Chrysis angustula* ♀ **100** *Chrysis mediata* ♀ **101** *Chrysis solida* ♀. Scale 1 mm.

**Figures 102–109.**
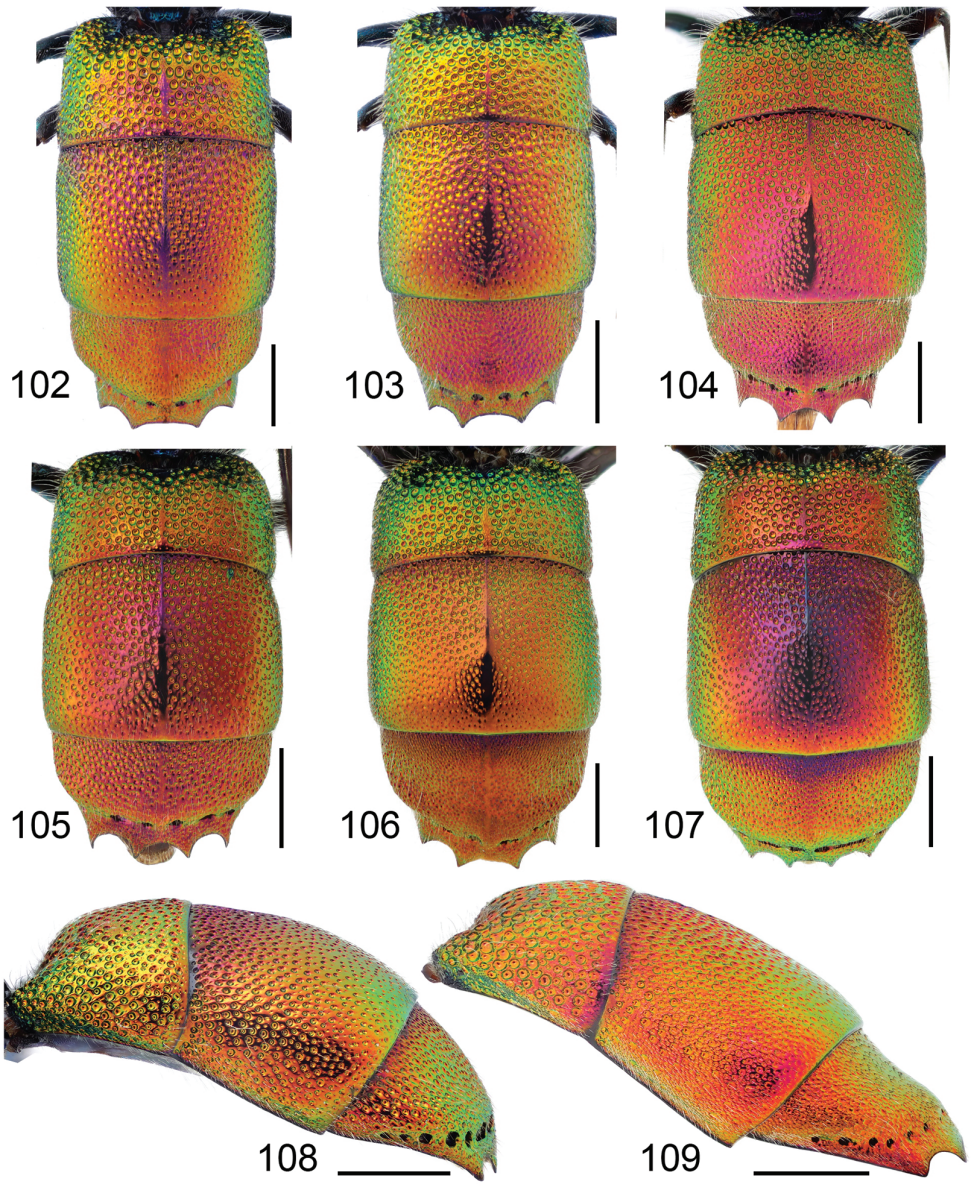
Metasoma, dorsal view: **102** *Chrysis longula* ♀ **103** *Chrysis corusca* ♀ **104** *Chrysis ignita* ♀ **105** *Chrysis ignita* ♂ **106** *Chrysis borealis* sp. n. ♀ **107** *Chrysis impressa* ♂. Metasoma, lateral view: **108** *Chrysis impressa* ♂ **109** *Chrysis subcoriacea* ♀. Scale 1 mm.

**Figures 110–125.**
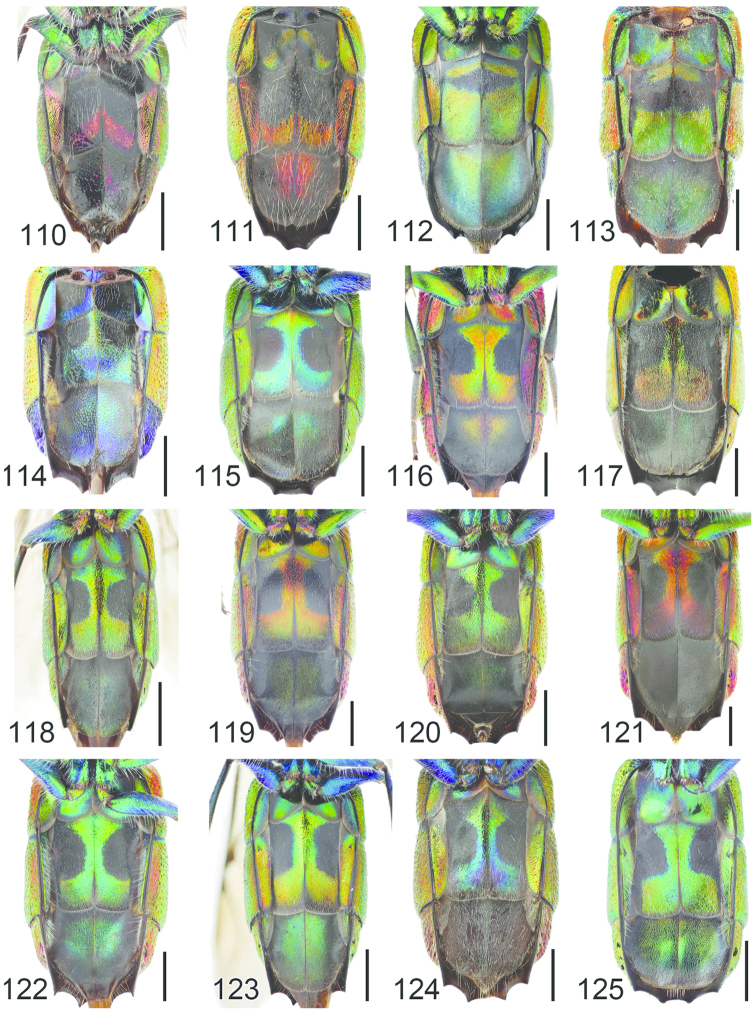
Metasoma, ventral view: **110** *Chrysis bicolor* ♀, **111** *Chrysis illigeri* ♀ **112** *Chrysis equestris* ♀ **113** *Chrysis zetterstedti* ♀ **114** *Chrysis splendidula* ♀ **115** *Chrysis vanlithi* ♀ **116** *Chrysis impressa* ♀ **117** *Chrysis subcoriacea* ♀ **118** *Chrysis leptomandibularis* ♀ **119** *Chrysis angustula* ♀ **120** *Chrysis solida* ♀ **121** *Chrysis longula* ♀ **122** *Chrysis ignita* ♀ **123** *Chrysis corusca* ♀ **124** *Chrysis borealis* sp. n. ♀ **125** *Chrysis solida* ♂. Scale 1 mm.

**Figures 126–137.**
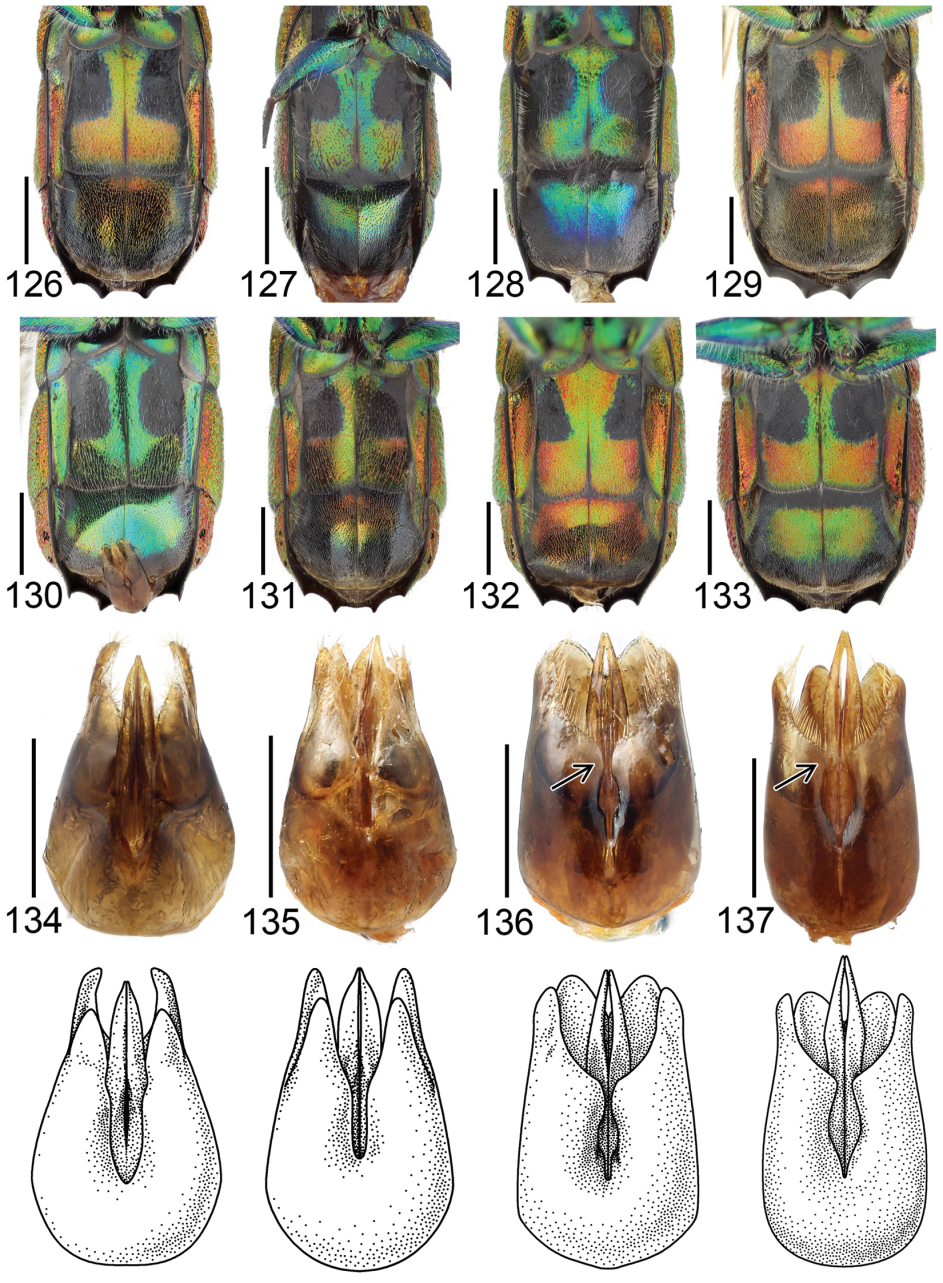
Metasoma, ventral view: **126** *Chrysis angustula* ♂ **127** *Chrysis leptomandibularis* ♂ **128** *Chrysis corusca* ♂ **129** *Chrysis subcoriacea* ♂ **130** *Chrysis ignita* ♂ **131** *Chrysis schencki* ♂ **132** *Chrysis impressa* ♂ **133** *Chrysis borealis* sp. n. ♂. Genital capsule, dorsal view: **134** *Chrysis equestris* ♂ **135** *Chrysis zetterstedti* ♂ **136** *Chrysis borealis* sp. n. ♂ **137** *Chrysis solida* ♂. Scale 1 mm (Figs **126–133**) and 0.5 mm (Figs **134–137**).

**Figures 138–144.**
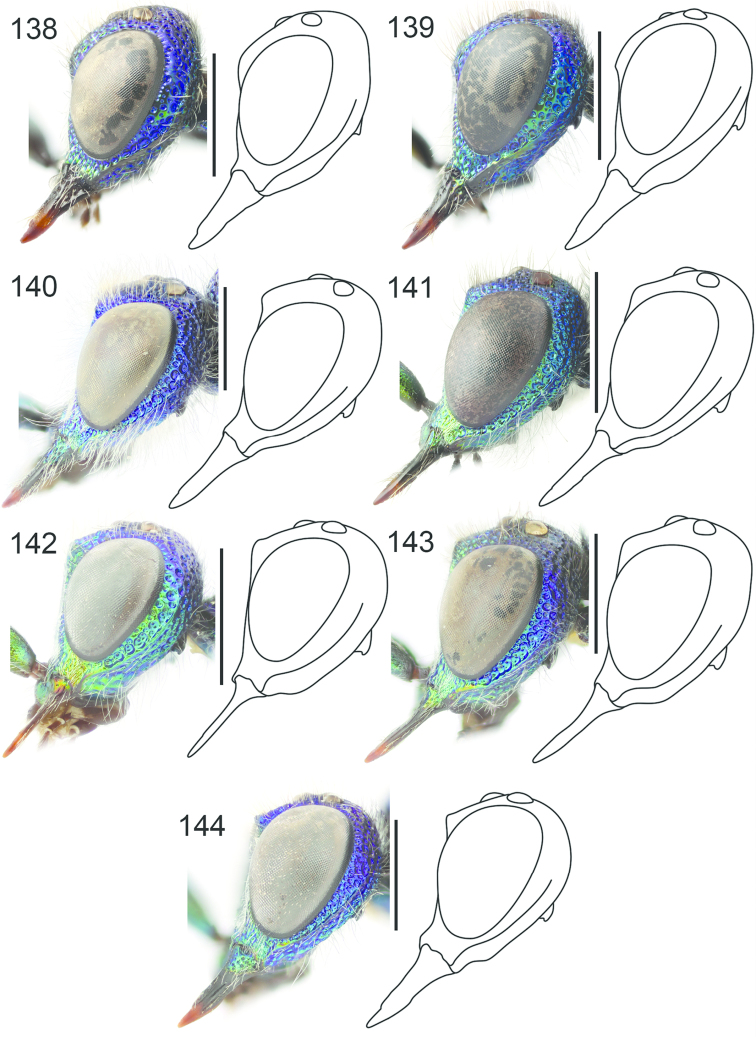
Head, lateral view: **138** *Chrysis bicolor* ♀ **139** *Chrysis illigeri* ♀ **140** *Chrysis vanlithi* ♀ **141** *Chrysis impressa* ♀ **142** *Chrysis leptomandibularis* ♀ **143** *Chrysis schencki* ♀ **144** *Chrysis corusca* ♀. Scale 1 mm.

**Figures 145–149.**
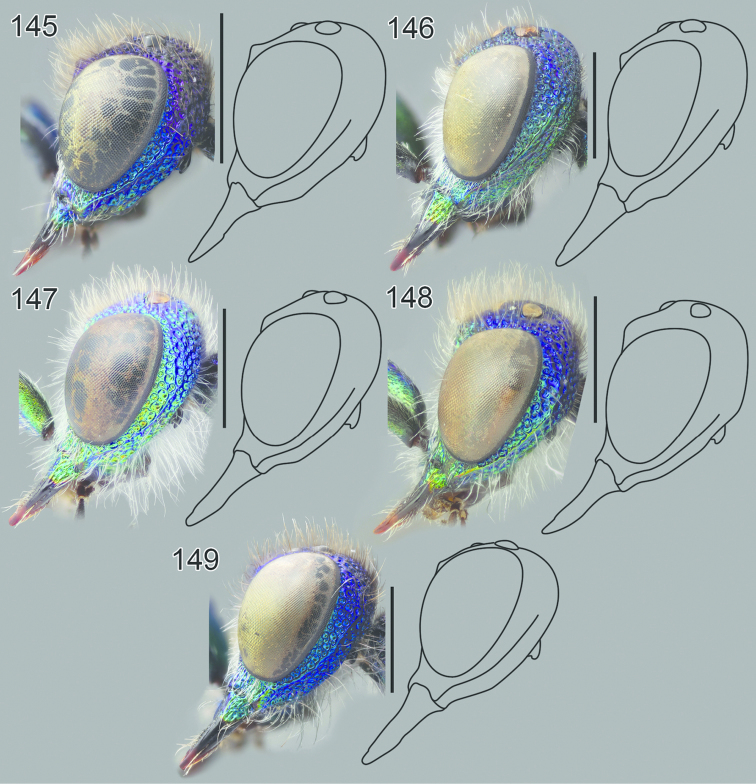
Head, lateral view: **145** *Chrysis leptomandibularis* ♂ **146** *Chrysis subcoriacea* ♂ **147** *Chrysis ignita* ♂ **148** *Chrysis schencki* ♂ **149** *Chrysis impressa* ♂. Scale 1 mm.

**Figures 150–164.**
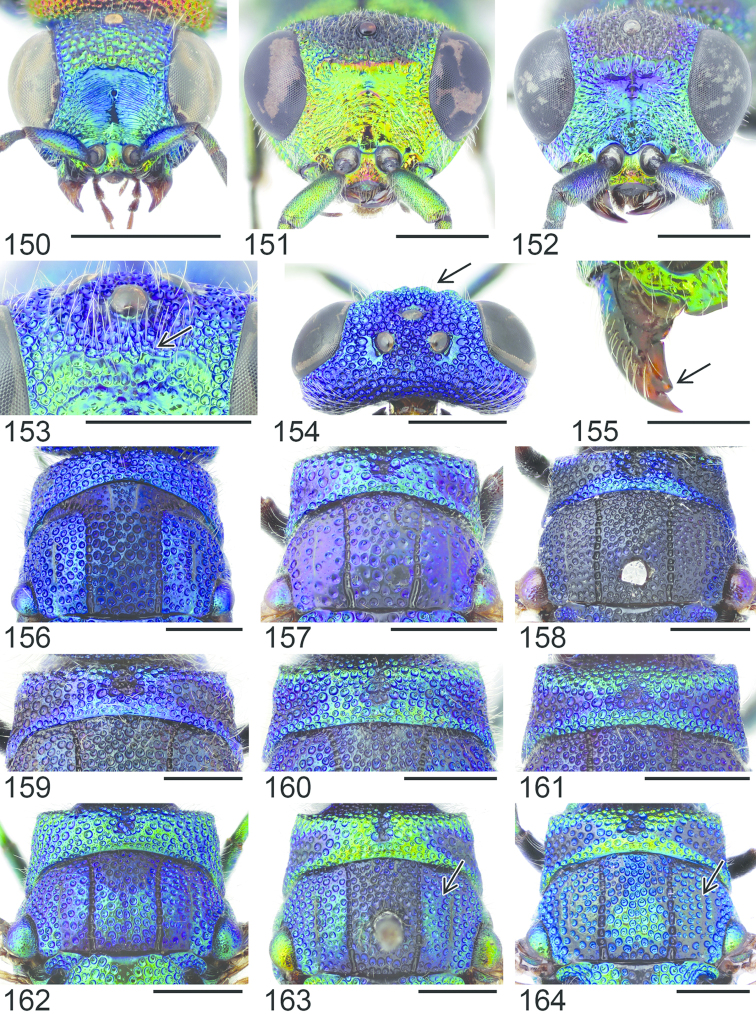
Head, frontal view (arrow indicating frontal carina): **150** *Chrysis leachii* ♀ **151** *Chrysis equestris* ♀ **152** *Chrysis zetterstedti* ♀ **153** *Chrysis terminata* ♀. Head, dorsal view (arrow indicating frontal carina): **154** *Chrysis terminata* ♀. Mandible (arrow indicating subapical tooth): **155** *Chrysis brevitarsis* ♀. Pronotum and mesoscutum, dorsal view: **156** *Chrysis indigotea* ♀, **157** *Chrysis brevitarsis* ♀ **158** *Chrysis borealis* sp. n. ♀. Pronotum, dorsal view: **159** *Chrysis vanlithi* ♀ **160** *Chrysis subcoriacea* ♂ **161** *Chrysis longula* ♂. Pronotum and mesoscutum, dorsal view (arrow indicating lateral field of mesoscutum): **162** *Chrysis clarinicollis* ♀ **163** *Chrysis ignita* ♀ **164** *Chrysis impressa* ♀. Scale 1 mm.

**Key to *Chrysis* species of the Nordic and Baltic countries**

**Table:** 

1	Posterior margin of T3 medially pointed or rounded without teeth (Fig. 77)	**2**
–	Posterior margin of T3 with distinct teeth or angular prominences (Figs 78–88)	**4**
2	Posterior margin of T3 almost evenly rounded, medially not pointed. Mesoscutum medially dark blue or blackish and laterally green or blue with golden reflections	***Chrysis gracillima* Förster**
–	Posterior margin of T3 medially somewhat pointed (Fig. 77). Mesoscutum completely red or golden greenish, medially not darker than laterally	**3**
3	Scapal basin largely smooth (female) or finely punctured (male) without fine cross-ridging. Mesoscutellum blue. Larger species, 4–8 mm	***Chrysis succincta* Linnaeus**
–	Scapal basin medially with fine cross-ridging in both sexes (Fig. 150). Mesoscutellum golden red or greenish-golden. Smaller species, 3–6 mm	***Chrysis leachii* Shuckard**
4	Medial teeth on the posterior margin of T3 extending distinctly further posteriorly than lateral teeth, and usually located in close proximity (Fig. 78)	**5**
–	Medial teeth on the posterior margin of T3 not extending distinctly further posteriorly than lateral teeth, and/or not located in close proximity (Fig. 79–88)	**7**
5	Mesoscutum blue or blackish. Second metatarsomere at least 3.5 times as long as broad (Fig. 171)	***Chrysis westerlundi* Trautmann**
–	Mesoscutum golden red or golden green. Second metatarsomere at most 2.5 times as long as broad	**6**
6	Malar space shorter, at most 0.75 times basal width of mandible (Fig. 138). Punctation of T2 dense. Metascutellum raised medially. Black spots of S2 strongly oblique posteriorly in female (Fig. 110)	***Chrysis bicolor* Lepeletier**
–	Malar space longer, in profile equal to basal width of mandible (Fig. 139). Punctation of T2 sparser. Metascutellum flat medially. Black spots of S2 not strongly oblique posteriorly in female (Fig. 111)	***Chrysis illigeri* Wesmael**
7	Posterior margin of T3 with six teeth (Figs 80–81)	**8**
–	Posterior margin of T3 with four teeth, these sometimes only shallow projections (Figs 79, 82–88)	**10**
8	Metasoma dorsally purple red. Lateral teeth of T3 sharp (Fig. 81)	***Chrysis sexdentata* Christ**
–	Metasoma multicoloured, T1 and T2 dark blue or black with golden green or golden red bands posteriorly. Lateral teeth of T3 rounded (Fig. 80)	**9**
9	Black spots of S2 fused and short, not or only slightly extending to lateral margins of sternite (Fig. 112). Ovipositor relatively broad and strongly chitinised (Fig. 89). T5 of female with transverse striae and longitudinal medial groove (Fig. 89). Head distinctly wider than high (Fig. 151). Male genitalia with broad notch between parameres, gonostyle very short and cuspis apically curved (Fig. 134). Propodeal tooth slightly convex or straight ventrally	***Chrysis equestris* Dahlbom**
–	Black spots of S2 fused and broad, widely extending to the lateral margins of the sternite (Fig. 113). Ovipositor narrow and weakly chitinised (Fig. 90). T5 of female without transverse striae and medial groove (Fig. 90). Head only slightly wider than high (Fig. 152). Male genitalia with narrow notch between parameres, gonostyle apically elongated (as long as cuspis) and cuspis apically straight (Fig. 135). Propodeal tooth weakly lobate ventrally	***Chrysis zetterstedti* Dahlbom**
10	Metasoma completely blue or blue-green, without red colour	**11**
–	Metasoma not completely blue or blue-green, always with red or golden colour	**12**
11	Mesoscutum medially not darker than laterally. Black spots of S2 large and long, almost extending to middle of sternite. T3 of female with distinct transverse bulge anterior to pit row. Male with short triangular apical teeth separated by wide intervals (Fig. 83). Ovipositor narrow (as in Fig. 92)	***Chrysis iris* Christ**
–	Mesoscutum distinctly darker medially than laterally (Fig. 156). Black spots of S2 smaller, distinctly separated in the middle. T3 of female without transverse bulge anterior to pit row. Male with spiniform apical teeth separated by narrow intervals (Fig. 82). Ovipositor broad (as in Fig. 91)	***Chrysis indigotea* Dufour & Perris**
12	T1 blue, T2 entirely golden red (female) or with large dark blue dorsal spot (male) (Fig. 93)	***Chrysis fulgida* Linnaeus**
–	Both T1 and T2 golden red or reddish	**13**
13	T3 completely green, blue or violet, in contrast with colour of T1 and T2	**14**
–	T3 golden red or reddish, at most with blue or violet apical rim	**17**
14	Mesosoma dorsally bright red	**15**
–	Mesosoma dorsally blue-green or blue-violet	**16**
15	Setae medially on metatibia longer than width of tibia (Fig. 166). Head completely blue-green or blue-violet. Mesoscutum completely red in male	***Chrysis viridula* Linnaeus**
–	Setae medially on metatibia shorter than width of tibia (Fig. 165). Head dorsally red in female. Mesoscutum medially green or blue in male	***Chrysis pulcherrima* Lepeletier**
16	Body slender, metasoma narrower (Fig. 94). T2 without or with very weak longitudinal keel medially (Fig. 94), posteriorly without lifted margin. Punctation of T2 relatively fine and sparse, interstices as large as or larger than puncture diameter (Fig. 94). Head in frontal view as wide as high. Black spots of S2 three quarters length of sternite	***Chrysis rutilans* Olivier**
–	Body robust, metasoma broader (Fig. 95). T2 with a distinct smooth longitudinal keel medially (Fig. 95), posteriorly with slightly raised margin. Punctation of T2 relatively coarse and dense, interstices smaller than puncture diameter (Fig. 95). Head in frontal view slightly wider than high. Black spots of S2 half of length of sternite (Fig. 114)	***Chrysis splendidula* Rossi**
17	Apical rim of T3 blue or violet, remaining tergite red	**18**
–	Apical rim of T3 golden red, of same colour as remaining tergite	**19**
18	Meso- and metascutellum golden red	***Chrysis scutellaris* Fabricius**
–	Mesosoma entirely blue-violet	***Chrysis graelsii* Guérin-Méneville**
19	Spurs of metatibia approximately equal in length (Fig. 167). Female metatarsus shorter than metatibia, second tarsomere twice as long as broad in lateral view (Fig. 169). Mandible thicker (in male, medial width of mandible about two thirds of its basal width; in female, medial width of mandible more than half of its basal width), with or without subapical tooth. Flagellomeres distinctly nodular in female (Fig. 172)	**20**
–	Spurs of metatibia distinctly unequal in length (Fig. 168). Female metatarsus longer than metatibia, second tarsomere at least three times longer than broad in lateral view (Fig. 170). Mandible thinner (in male, medial width of mandible less than two thirds of its basal width; in female, medial width of mandible not more than half of its basal width), always without subapical tooth. Flagellomeres not distinctly nodular in female	**21**
20	Mandible with subapical tooth (Fig. 155). Female mesoscutum laterally with scattered punctation (Fig. 157)	***Chrysis brevitarsis* Thomson**
–	Mandible without subapical tooth. Female mesoscutum laterally with dense punctation	***Chrysis pseudobrevitarsis* Linsenmaier**
21	Pronotum short, length less than one fourth of its width (Fig. 159). Malar space long, approximately as long as broad in female (Fig. 140), and somewhat shorter in male. F1 of antenna without metallic sheen. Frons with dense adpressed white pubescence	**22**
–	Pronotum longer, length at least one fourth of its width (Figs 158, 160–164). Malar space short, shorter than broad in both female (Figs 141–144) and male (Figs 145–149). F1 with metallic sheen (often very weak). Frons usually with sparser and more erect pubescence	**23**
22	Punctation of tergites very fine and dense throughout, punctures of uniform size, surface dull (Fig. 96). Sternites and legs ventrally coppery red	***Chrysis ruddii* Shuckard**
–	Punctation of tergites coarser and sparser, punctures of variable size, surface shiny (Fig. 97). Sternites and legs ventrally always greenish (Fig. 115)	***Chrysis vanlithi* Linsenmaier**
23	Frontal carina with four tooth-like tubercles medially (Figs 153, 154). Punctation of tergites coarse throughout (as in Figs 104, 105). Head and mesosoma dorsally with white pubescence. Apical teeth of T3 relatively long and sharp (as in Figs 104, 105)	***Chrysis terminata* Dahlbom**
–	Frontal carina without four tooth-like tubercles. Punctation of tergites, colour of pubescence and shape of apical teeth variable	**24**
24	Pronotum and mesoscutellum uniformly greenish (Fig. 162). T1 anteriorly and laterally blue-green or green, dorsally golden red (Fig. 98). Shape of metasoma broad and compact (Fig. 98). Apical rim short and intervals between apical teeth shallow (Fig. 98)	***Chrysis clarinicollis* Linsenmaier**
–	Pronotum blue or violet framed by lighter colour, mesoscutellum medially darker than laterally (Figs 158, 160, 161, 163, 164). T1 laterally golden red or only slightly greenish, dorsally golden red. Shape of metasoma, apical rim and apical teeth variable (Figs 99–107)	**25**
25	Female. With ovipositor. Posterior margin of S4 opaque and angled (Figs 116–124). T3 dorsally straight or concave in lateral view (Fig. 109)	**26**
–	Male. Without ovipositor. Posterior margin of S4 semitransparent and almost straight (Figs 125–133). T3 dorsally convex in lateral view (Fig. 108)	**36**
26	T2 and T3 laterally completely dull, with dense coriaceous microsculpture between punctures (Fig. 109). Elongate and usually large species, ovipositor thin (as in Fig. 92), S2 red with long black spots (Fig. 117)	***Chrysis subcoriacea* Linsenmaier**
–	T2 and T3 laterally with shiny surface between punctures (as in Fig. 108). Body shape, breadth of ovipositor and colouration of S2 variable	**27**
27	Mandible extremely thin (in lateral view, medially as broad as apical segment of labial palp) (Fig. 142), dorsally smooth and impunctate. Body slender and elongate, metasoma with almost parallel sides (as in Fig. 99). S2 greenish with short, rounded black spots (Fig. 118). Mesoscutum laterally with wide, strongly shining interstices between punctures	***Chrysis leptomandibularis* Niehuis**
–	Mandible thicker (in lateral view, medially broader than apical segment of labial palp), dorsally always punctate. Shape of body, colouration of S2 and punctation of mesoscutum variable	**28**
28	Body slender and elongate, metasoma with almost parallel sides (Fig. 99). Punctation of T2 anteriorly finer than on T1, posteriorly very sparse (Fig. 99). T3 long, deeply depressed medially and strongly shining. Apical teeth short and blunt, with a wide and shallow central interval (Fig. 99). S2 with rectangular black spots and golden red colour (Fig. 119). Ovipositor narrow (as in Fig. 92)	***Chrysis angustula* Schenck**
–	Body not as slender and elongate (Figs 100–104, 106). Punctation of T2 variable (Figs 100–104, 106). T3 shorter, more shallowly depressed medially and not as strongly shining. Apical teeth longer and/or sharper, shape of central interval variable (Figs 100–104, 106). Black spots of S2 not as distinctly rectangular, colouration variable (Figs 116, 120–124). Ovipositor narrow (Fig. 92) or broad (Fig. 91)	**29**
29	Ovipositor broad (T5 broader than long) (Fig. 91), often only its apex exerted (Fig. 100). S2 blue-green with relatively short and rounded black spots (Fig. 120). Mandible thick (in lateral view, medial width of mandible about half of its basal width) (as in Fig. 144). Apical rim of T3 long and central interval between apical teeth often angular	**30**
–	Ovipositor narrow (T5 longer than broad) (Fig. 92). Colouration of S2 (Figs 116, 121–124) and thickness of mandible variable (Figs 141, 143, 144). Apical rim usually shorter and central interval more widely arcuate	**31**
30	Metasoma broader, with slightly convex sides (Fig. 100). Punctation of T2 anteriorly sparser and finer, with shining interstices (Fig. 100). T3 with slightly sparser punctation, its surface thereby shinier (Fig. 100). Head narrower, in frontal view only slightly broader than high. Colour of mesosoma lighter and more greenish. Usually larger species. Hosts soil-nesting species of *Odynerus*	***Chrysis mediata* Linsenmaier**
–	Metasoma more elongate, with more parallel sides (Fig. 101). Punctation of T2 anteriorly denser and coarser, without shining interstices (Fig. 101). T3 with dense punctation, its surface dull (Fig. 101). Head broader, in frontal view distinctly broader than high. Colour of mesosoma darker and more bluish. Usually smaller species. Hosts cavity-nesting species of *Ancistrocerus* and *Euodynerus*	***Chrysis solida* Haupt**
31	Metasoma elongate with almost parallel sides (Figs 102, 103). S2 with long and narrow black spots (Figs 121, 123). Mandible thicker (medial width of mandible about half of its basal width) (Fig. 144)	**32**
–	Metasoma with more convex sides (Figs 104, 106). S2 with shorter black spots (Figs 116, 122, 124). Mandible thinner (medial width of mandible less than half of its basal width) (Figs 141, 143)	**33**
32	T2 anteriorly with dense, deep and coarse punctation, punctures becoming much sparser and finer posteriorly (Fig. 102). T3 shiny, without microsculpture (Fig. 102). S2 red (Fig. 121).Mandible longer and thinner. Metascutellum medially flat. Usually larger species	***Chrysis longula* Abeille de Perrin**
–	T2 anteriorly with somewhat sparser and finer punctation, punctures becoming slightly sparser and finer posteriorly (Fig. 103). T3 shiny or with weak microsculpture (Fig. 103). S2 greenish (Fig. 123). Mandible shorter and thicker. Metascutellum medially more convex. Usually smaller species	***Chrysis corusca* Valkeila**
33	Apical teeth of T3 sharply produced and apical rim with almost parallel lateral margins (Fig. 104). Punctation of T2 and T3 coarse throughout (Fig. 104). Pubescence of vertex whitish. S2 green or blue with rectangular black spots (Fig. 122). Mesoscutum shiny blue or greenish, punctures of same colour as interstices (Fig. 163). Medial furrow of pronotum narrow (Fig. 163)	***Chrysis ignita* (Linnaeus)**
–	Apical teeth of T3 not as sharply produced and apical rim with more angled lateral margins (Figs 84, 106). Punctation of T2 and T3 finer (Figs 84, 106). Pubescence of vertex whitish or brownish. Colouration of S2 (Figs 116, 124) and mesoscutum (Figs 158, 164) variable. Medial furrow of pronotum broader (Figs 158, 164)	**34**
34	Mandible very thin (medial width of mandible not more than one third of its basal width), basally strongly narrowing in lateral view (Fig. 143). Scapal basin with sparse and well defined punctation. Vertex with light brown pubescence. T3 relatively dull with distinct microsculpture between punctures. Mesoscutum dark blue to almost black. Metasoma usually more elongate	***Chrysis schencki* Linsenmaier**
–	Mandible thicker (medial width of mandible more than one third of its basal width), gradually narrowing towards the apex (Fig. 141). Scapal basin with denser and more coriaceous punctation. Vertex with brown or whitish pubescence. T3 usually shinier. Colour of mesoscutum variable (Figs 164, 158, 179). Metasoma usually broader and more compact	**35**
35	Mesoscutum laterally with green or blue punctures and black interstices (Fig. 164). Punctures relatively large (Fig. 164). F1 1.3–1.5 times as long as F2. S2 posteriorly usually red with relatively rounded black spots (Fig. 116). Pubescence of vertex brownish	***Chrysis impressa* Schenck**
–	Mesoscutum entirely blue, violet or black, punctures and interstices generally of same colour (Figs 158, 179). Punctures smaller (Figs 158, 179). F1 1.5–1.7 times as long as F2. S2 dark green or blue with almost rectangular black spots (Fig. 124). Pubescence of vertex whitish or light brown	***Chrysis borealis* sp. n.**
36	(Males.) Mandible thin (medial width of mandible about one third of its basal width) (Fig. 145), dorsally smooth, without or with only barely visible punctures. Body small and slender, metasoma elongate with almost parallel sides (habitus similar to *Chrysis angustula*). S2 greenish with short rounded black spots (Fig. 127). Posterior margin of propodeal tooth virtually straight and perpendicular to body axis	***Chrysis leptomandibularis* Niehuis**
–	Mandible thicker (medial width of mandible more than one third of its basal width), its dorsal surface always with small punctures. Body size and shape variable. Colouration of S2 variable (Figs 125, 126, 128–133). Posterior margin of propodeal tooth straight, convex or concave	**37**
37	Colour of S2 golden or reddish, with long rectangular black spots (Fig. 126). Body slender, metasoma with approximately parallel sides (habitus similar to *Chrysis leptomandibularis*). Punctation of T2 anteriorly usually finer than on T1, becoming sparser posteriorly, surface thereby strongly shining. T3 shiny, transition of lateral margin into lateral apical teeth straight, apical rim and teeth short (Fig. 86)	***Chrysis angustula* Schenck**
–	Colour of S2 variable, black spots usually not rectangular (Figs 125, 128–133). Body usually more robust, metasoma with more convex sides. Punctation of T2 anteriorly slightly finer or as coarse as on T1, surface posteriorly not as shining. T3 shiny or dull, transition of lateral margin into lateral apical teeth straight or concave, apical rim and teeth longer (Figs 85, 87–88)	**38**
38	Inner margin of paramere angled (Fig. 137). Punctation of T2 anteriorly usually finer than on T1. T3 with very fine and regular punctation. S2 usually greenish with relatively short black spots (Fig. 125). Mandible relatively thick (in lateral view, medial width of mandible more than half of its basal width)	**39**
–	Inner margin of paramere rounded (Fig. 136). Punctation of T2 anteriorly usually not finer than on T1. T3 with coarser and/or more irregular punctation. S2 green, golden or red with often larger black spots (Figs 128–133). Mandible thick or thin	**40**
39	The following two species are not always separable. Metasoma with slightly convex sides (approximately as in Fig. 100). Head narrower, in frontal view only slightly broader than high. Colour of mesosoma predominantly lighter, often greenish. Usually larger species. Hosts soil-nesting species of *Odynerus*	***Chrysis mediata* Linsenmaier**
–	Metasoma with more parallel sides (approximately as in Fig. 101). Head narrower, in frontal view distinctly broader than high. Colour of mesosoma predominantly darker, violet, blue or blue-green. Usually smaller species. Hosts cavity-nesting species of *Ancistrocerus* and *Euodynerus*	***Chrysis solida* Haupt**
40	Mandible thick (medial width of mandible more than half of its basal width), its margins basally straight in lateral view (Fig. 146). T3 relatively shiny, transgression of lateral margin into lateral apical teeth straight (Fig. 87) or slightly concave (Fig. 88). F1 longer than F2 (Figs 173–174). Metasoma with more parallel sides	**41**
–	Mandible thinner (medial width of mandible not more than half of its basal width), its margins basally more or less concave in lateral view (Figs 147–149). T3 relatively dull, transgression of lateral margin into lateral apical teeth slightly (Fig. 85) or strongly concave (Fig. 105). F1 longer than or as long as F2 (Figs 175–178). Metasoma with more convex sides	**43**
41	S2 mainly green, its pubescence dense and long (Fig. 128). F1 distally without or with a very shallow, inconspicuous impression (Fig. 173). Propodeal tooth usually laterally straight or convex	***Chrysis corusca* Valkeila**
–	S2 mainly red or golden, its pubescence variable (Fig. 129). F1 distally with a shallow impression (Fig. 174). Propodeal tooth usually laterally concave	**42**
42	Pronotum without sharply contrasting border between the darker middle part and the lighter margins (Fig. 160). Punctation of T2 anteriorly coarse or relatively fine. Transgression of lateral margin of T3 into lateral apical teeth straight (Fig. 87). Central interval of apical teeth more angulate, and pits of apical rim smaller (Fig. 87)	***Chrysis subcoriacea* Linsenmaier**
–	Pronotum usually with sharply contrasting border between the darker middle part and the lighter margins (Fig. 161). Punctation of T2 anteriorly coarse. Transgression of lateral margin of T3 into lateral apical teeth slightly concave (Fig. 88). Central interval of apical teeth more arcuate, and pits of apical rim larger (Fig. 88)	***Chrysis longula* Abeille de Perrin**
43	F1 approximately as long as or slightly (not more than 1.2×) longer than F2 (Fig. 175). T2 coarsely punctured throughout (Fig. 105). Punctation of T3 coarse and dense (Fig. 105). Apical teeth of T3 long and sharp (Fig. 105). Pubescense of T3 relatively long. Main colour of sternites green (Fig. 130). Black spots of S2 relatively large and rectangular (Fig. 130). Pubescence of vertex often whitish (Fig. 147)	***Chrysis ignita* (Linnaeus)**
–	F1 1.2–1.5 times as long as F2 (Figs 176–178). T2 more finely punctured, punctures becoming slightly sparser posteriorly (Fig. 107). Punctation of T3 finer (Figs 85, 107). Apical teeth of T3 not as sharp (Figs 85, 107). Pubescense of T3 relatively short. Main colour of sternites variable (Figs 131–133). Black spots of S2 not as rectangular (Figs 131–133). Pubescence of vertex usually brownish (Figs 148, 149)	**44**
44	The following three species are not always separable. Mandible thinner, its margins basally concave in lateral view (Fig. 148). F1 1.2–1.4 times as long as F3 (Fig. 176). Sternites usually golden green or golden red (Fig. 131). Size of black spots of S2 variable (Fig. 131)	***Chrysis schencki* Linsenmaier**
–	Mandible thicker, its margins basally almost straight in lateral view (Fig. 149). F1 1.2–1.5 times as long as F2 (Figs 177–178)	**45**
45	F1 1.2–1.4 times as long as F2 (Fig. 177). Size of black spots of S2 variable (Fig. 132). Punctation of mesoscutum coarser, punctures often with lighter colour compared to interstices	***Chrysis impressa* Schenck**
–	F1 1.3–1.5 times as long as F2 (Fig. 178). Black spots of S2 large (Fig. 133). Punctation of mesoscutum finer, punctures not differing in colour from interstices	***Chrysis borealis* sp. n.**

##### *Chrysis varidens-gracillima* group

###### Chrysis gracillima

Taxon classificationAnimaliaHymenopteraChrysididae

Förster, 1853

Chrysis gracillima Förster, 1853: 328.
Chrysis saussurei Chevrier, 1862: 36.

####### Diagnosis.

Length 4–7 mm. The species is easy to recognise by the edentate posterior margin of T3 and the narrow, elongate body shape. Species of *Chrysura*, which are similarly coloured and also lack apical teeth, do not have the scapal basin or the dark apical rim, and are larger in size. The head and mesosoma are mainly blue or greenish with golden reflections, and the mesoscutum is medially contrastingly darker than laterally. The metasoma is completely golden red dorsally, but the apical rim is dark blue or blackish. The apical rim is wide, medially slightly undulating and laterally with angled margins. F2, F3 and F4 are ventrally slightly bulging in the male. The shape of the body is very slender and elongate in both sexes.

####### Distribution.

Estonia. Very rare. One female was collected on 14.VII.2015 in Reinu, southwestern Estonia (58.032°N, 24.747°E, leg. V. Soon). No other records are known from the Nordic and Baltic countries, but one female has been collected from Russia, close to the eastern border of Latvia (Pskov Oblast, Krasikovo, 23 km south of Sebezh, VII.1999, leg. A. Reschikov). – West Palearctic: Europe, northern Africa and Middle East (Linsenmaier 1997).

####### Biology.

Habitat: various biotopes with sun-exposed dead wood (Kunz 1994, Linsenmaier 1996, Rosa 2006). Adults often fly on wooden poles (Rosa 2006) and occasionally visit flowers of Apiaceae and Euphorbiaceae (Linsenmaier 1997). Flight period: June to August in Germany (Kunz 1994). Host: species of *Microdynerus* Thomson (Vespidae) (Friese 1883, Enslin 1929, Benno 1950, Wickl 2001) and possibly also *Trypoxylon clavicerum* Lepeletier & Serville (Crabronidae) (Morgan 1984). The only *Microdynerus* species found in Estonia is *Microdynerus parvulus* (Herrich-Schäffer), which could be the host of *Chrysis gracillima* at the northern limit of its distribution area.

##### *Chrysis succincta* group

###### Chrysis bicolor

Taxon classificationAnimaliaHymenopteraChrysididae

Lepeletier, 1806

Figs 110 , 138

Chrysis bicolor Lepeletier, 1806: 127.
Chrysis succincta var. virideocincta Trautmann, 1927: 160.

####### Diagnosis.

Length 5–8 mm. The species resembles *Chrysis illigeri*, but the malar space is shorter (Fig. 138), the punctation of T2 is denser and the metascutellum is more raised in profile. Also the black spots of S2 are posteriorly more oblique in the female (Fig. 110). The head and mesosoma are mainly blue or greenish, but the mesoscutum and anterior margin of the pronotum are red or golden red in the female and golden green to greenish in the male. The mesoscutum is usually darkened medially in the male. The metasoma is mainly red in the female and golden greenish in the male with a greenish, bluish or black apical rim. T2 often has a black patch dorsally in the female.

####### Distribution.

Denmark, Estonia, Finland, Latvia, Lithuania, Sweden. Relatively rare. – Trans-Palearctic: Europe, northern Africa, Russian Far East (Kimsey and Bohart 1991, Kurzenko and Lelej 2007).

####### Biology.

Habitat: sparsely vegetated sandy areas. Adults occasionally visit flowers of Apiaceae, Asteraceae, Euphorbiaceae and Rosaceae (Linsenmaier 1997, Rosa 2004, 2006, our own obs.). Flight period: early June to late August. Host: *Tachysphex obscuripennis* (Schenck) and *Tachysphex pompiliformis* (Panzer) (Crabronidae) (Morgan 1984, Saure 1998, Wickl 2001). In central and southern Europe also *Dinetus pictus* (Fabricius) (Crabronidae) (Gauss 1967).

###### Chrysis westerlundi

Taxon classificationAnimaliaHymenopteraChrysididae

Trautmann, 1927

Fig. 171

Chrysis succincta var. westerlundi Trautmann, 1927: 159.
Chrysis succincta var. *nordströmi* Trautmann, 1927: 159.
Chrysis westerlundi : Linsenmaier 1959: 113.

####### Diagnosis.

Length 7–9 mm. The species differs from other North European species of the *Chrysis succincta* group by its characteristic colouration: the head and mesosoma are completely dark blue or almost black dorsally in the female and green blue in the male. The metasoma is dorsally red in both sexes and anteriorly greenish in the male. The metatarsus is long, the second tarsomere is at least 3.5 times as long as broad (Fig. 171). Superficially, the species can resemble similarly coloured species of the *Chrysis ignita* group, but the two central apical teeth are close to each other and extend further posteriorly than the lateral teeth (as in Fig. 78). Also the black spots of S2 are large and not separated by a metallic central line.

####### Distribution.

Finland. Very rare, only eight specimens (6 females and 2 males) are known. – West Palearctic: the species has been found only from Finland and Russian Fennoscandia (Paukkunen et al. 2014).

####### Biology.

Habitat: sparsely vegetated sandy areas. One specimen was caught with a Malaise trap in a bog. Flight period: mid-June to early August. Host: unknown.

####### Remarks.

The species might be conspecific with the Far Eastern species *Chrysis cavaleriei* du Buysson, 1908 and/or the North American species *Chrysis provancheri* Schulz, 1906 (= *aurichalcea* Provancher, 1881) (Paukkunen et al. 2014).

**Figures 165–178.**
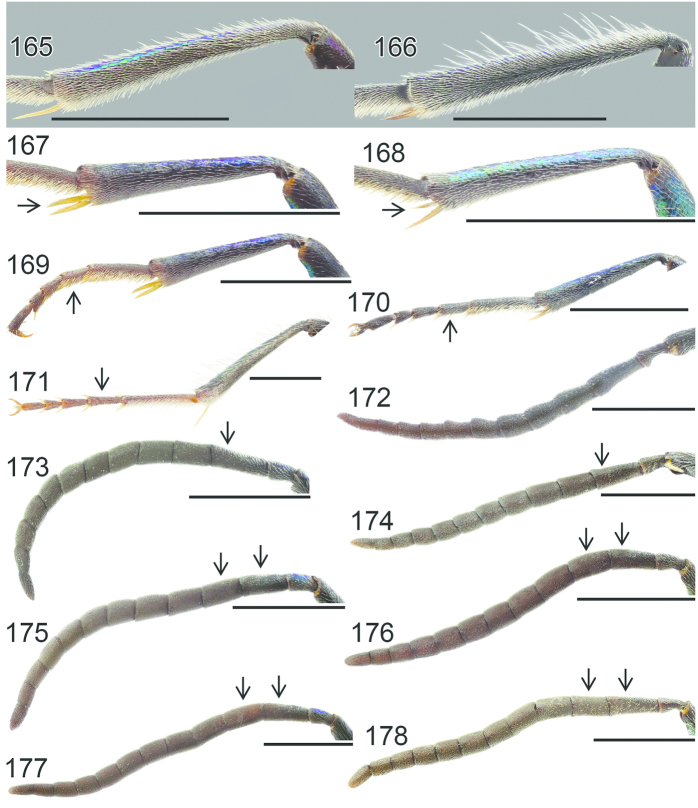
Metatibia: **165** *Chrysis pulcherrima* ♀ **166** *Chrysis viridula* ♀. Mesotibia (arrow indicating spurs): **167** *Chrysis pseudobrevitarsis* ♀ **168** *Chrysis impressa* ♀. Hindleg (arrow indicating second tarsomere): **169** *Chrysis pseudobrevitarsis* ♀ **170** *Chrysis impressa* ♀ **171** *Chrysis westerlundi* ♀. Antenna (arrow indicating F1 or F1 and F2): **172** *Chrysis brevitarsis* ♀ **173** *Chrysis corusca* ♂ **174** *Chrysis subcoriacea* ♂ **175** *Chrysis ignita* ♂ **176** *Chrysis schencki* ♂ **177** *Chrysis impressa* ♂ **178** *Chrysis borealis* sp. n. ♂. Scale 1 mm.

###### Chrysis illigeri

Taxon classificationAnimaliaHymenopteraChrysididae

Wesmael, 1839

Figs 78 , 111 , 139

Chrysis illigeri Wesmael, 1839: 176.
Chrysis succincta var. chrysoprasina Trautmann, 1927: 159, not Förster, 1853.
Chrysis succincta f. *helléni* Balthasar, 1953: 285, replacement name for *chrysoprasina* Trautmann, 1927.
Chrysis helleni Linsenmaier, 1959: 113, not Balthasar, 1953.

####### Diagnosis.

Length 5–8 mm. The species resembles *Chrysis bicolor* in colouration, but the anterior margin of the pronotum, the mesoscutum and the metasoma are usually mainly red in the male (not greenish), and the posterior margins of the black spots of S2 are not as oblique in the female (Fig. 111). Compared to *Chrysis bicolor*, the malar space is longer (Fig. 139), the punctation of T2 is sparser and the metascutellum is not as elevated in profile.

####### Distribution.

Denmark, Estonia, Finland, Latvia, Lithuania, Norway, Sweden. Common. – West Palearctic: Europe (Linsenmaier 1997).

####### Biology.

Habitat: sparsely vegetated sandy areas. Adults occasionally visit flowers of Apiaceae, Asteraceae, Euphorbiaceae and Rosaceae (Linsenmaier 1997, Rosa 2004, our own obs.). Flight period: late May to late August. Host: *Tachysphex pompiliformis* (Panzer) (Crabronidae) (Westrich 1983, Morgan 1984, Saure 1998).

###### Chrysis succincta

Taxon classificationAnimaliaHymenopteraChrysididae

Linnaeus, 1767

Fig. 77

Chrysis succincta Linnaeus, 1767: 947.

####### Diagnosis.

Length 4–8 mm. The species can be differentiated from other Nordic and Baltic species of the *Chrysis succincta* group by having a rounded or slightly pointed, edentate posterior margin of T3 (Fig. 77) and a blue mesoscutellum. In *Chrysis leachii*, the posterior margin of T3 is also edentate, but the mesoscutellum is mostly red. The scapal basin is largely shiny, without cross-ridging in the female, and mostly densely microsculptured in the male, with only a narrow central dull longitudinal line. The head, most of the mesosoma, base of T1 and the apical rim are green or blue, whereas the anterior margin of the pronotum and the mesoscutum are red in the female and golden green in the male. The metasoma is dorsally mainly red, or often greenish in the male.

####### Distribution.

Denmark, Latvia, Lithuania. Rare. – Trans-Palearctic: Europe, northern Africa, Russian Far East (Linsenmaier 1997, Kurzenko and Lelej 2007). (In northern Africa represented by ssp. *succinctula* Linsenmaier, 1959.)

####### Biology.

Habitat: sparsely vegetated sandy areas. In central Europe typical habitats include e.g. embankments, wastelands and margins of pine forests (Trautmann 1930, Ressl 1973, Molitor 1935). Adults occasionally visit flowers of Apiaceae, Asteraceae and Euphorbiaceae (Molitor 1935, Linsenmaier 1997). Flight period: mid-June to mid-August. Host: unknown.

###### Chrysis leachii

Taxon classificationAnimaliaHymenopteraChrysididae

Shuckard, 1837

Fig. 150

Chrysis Leachii Shuckard, 1837: 168.

####### Diagnosis.

Length 3–6 mm. The species is similar to *Chrysis succincta* in having an edentate posterior margin of T3 (as in Fig. 77), but the margin is medially more sharply pointed, especially in the female. The center of the scapal basin is finely cross-ridged (Fig. 150) or it is dull and densely microsculptured. The colouration resembles that of *Chrysis succincta*, but the mesoscutellum is red (not blue) and the tergites are often posteriorly narrowly green or blue. The males are usually more greenish than females.

####### Distribution.

Denmark. Very rare. Only one male and one female are known, recorded from the island of Funen (Helnæs, 19.VIII.1918, leg. L. Jørgensen). – West Palearctic: Europe, northern Africa, Asia Minor (Linsenmaier 1997, 1999).

####### Biology.

Habitat: steep sand and loess slopes and stone walls (Ressl 1966, Kusdas 1956, Gerth et al. 2010). Adults occasionally visit flowers of Apiaceae, Caryophyllaceae and Euphorbiaceae (Graffe 1895, Linsenmaier 1997, Rosa 2004). Flight period: June to August. Host: probably *Diodontus minutus* (Fabricius) and/or *Miscophus bicolor* Jurine (Crabronidae) (Trautmann and Trautmann 1919, Linsenmaier 1959, Heinrich 1964, Gerth et al. 2010). We consider records of other host species (e.g. *Tachysphex nitidus* (Spinola)) as uncertain due to lack of supporting information.

##### *Chrysis comparata* group

###### Chrysis scutellaris

Taxon classificationAnimaliaHymenopteraChrysididae

Fabricius, 1794

Chrysis scutellaris Fabricius, 1794: 458.
Chrysis segmentata Dahlbom, 1829: 9.

####### Diagnosis.

Length 6–9 mm. The species is easily recognised due to its unique colouration among North European chrysidids. The head and the mesosoma are mainly blue or green, but the meso- and metascutellum are red or golden. Usually also the frons, the anterior margin of the pronotum and the lateral sections of the mesoscutum have golden or coppery reflections. The metasoma is dorsally red, but the apical rim of T3 is contrastingly blue. The apical teeth are shallow and indistinct, whereby the apical rim may appear nearly rounded.

####### Distribution.

Denmark, Lithuania, Sweden. Rare. Only a few records from Denmark and Sweden (Scania) and one record from Lithuania are known (Orlovskytė et al. 2010, Sörensson et al. 2012). – West Palearctic: Europe and northern Africa (Linsenmaier 1997, 1999).

####### Biology.

Habitat: xerothermic sparsely vegetated sandy areas, often close to seashore. Adults often bask on sun-exposed pieces of wood or logs on the sand, and spend nights inside hollow plant stems (Sörensson et al. 2012). Occasionally they are found on flowers of Apiaceae, Asteraceae, Crassulaceae and Euphorbiaceae (Trautmann 1927, Heinrich 1964, Linsenmaier 1997, Rosa 2004). Flight period: late June to early August. Host: probably *Megachile leachella* Curtis (Megachilidae) (Sörensson et al. 2012). In central Europe, also *Pseudanthidium lituratum* (Panzer) (Megachilidae) (Schmid-Egger et al. 1995).

####### Remarks.

Mitochondrial DNA sequences available at the Barcode of Life Data System (Ratnasingham and Hebert 2007) indicate that Swedish specimens of *Chrysis scutellaris* differ genetically remarkably from central European specimens. The status of European populations should be studied more in the future.

##### *Chrysis splendidula* group

###### Chrysis splendidula

Taxon classificationAnimaliaHymenopteraChrysididae

Rossi, 1790

Figs 95 , 114

Chrysis splendidula Rossi, 1790: 78.

####### Diagnosis.

Length 5–8 mm. The colouration is similar to *Chrysis rutilans*, as the head, mesosoma and T3 are green, blue, or partially black, whereas T1 and T2 are golden red. Compared to *Chrysis rutilans*, the body is more robust and the punctation of T2 is coarser (Fig. 95). T2 has a distinct polished longitudinal keel medially and its posterior margin is narrowly raised (Fig. 95). The black spots of S2 are shorter (Fig. 114) and the head is broader than in *Chrysis rutilans*.

####### Distribution.

Latvia. Very rare. Only three records are known from eastern and central Latvia. – Trans-Palearctic: Europe, central Asia, Japan and Korea (Linsenmaier 1997, Kurzenko and Lelej 2007).

####### Biology.

Habitat: sparsely vegetated sandy areas, such as sand pits (Hoop 1971). Adults occasionally visit flowers of Apiaceae, Asteraceae and Euphorbiaceae (Linsenmaier 1997, Rosa 2004, 2006). Flight period: July and early August. Host: *Eumenes coarctatus* (Linnaeus) (Vespidae) (Martynova and Fateryga 2015). Possibly also *Eumenes mediterraneus* Kriechbaumer and *Eumenes pomiformis* (Fabricius) (du Buysson 1895, Ferton 1910, Martynova and Fateryga 2015) Other host species reported for *Chrysis splendidula*, such as *Gymnomerus laevipes* (Shuckard), *Symmorphus allobrogus* (Saussure) (Vespidae), *Trypoxylon figulus* (Linnaeus) (Crabronidae) and *Osmia andrenoides* Spinola (Megachilidae), concern actually *Chrysis rutilans* or are unreliable (Martynova and Fateryga 2015).

###### Chrysis rutilans

Taxon classificationAnimaliaHymenopteraChrysididae

Olivier, 1791

Fig. 94

Chrysis rutilans Olivier, 1791: 676.
Chrysis splendidula of authors, not Rossi, 1790.
Chrysis insperata ? Chevrier, 1870: 265.

####### Diagnosis.

Length 5–9 mm. The species resembles *Chrysis splendidula* in colouration, but the body is more slender and the punctation of T2 is finer (Fig. 94). Also, T2 does not have a distinct smooth longitudinal keel or a raised posterior margin (Fig. 94). The black spots of S2 are longer and the head is narrower than in *Chrysis splendidula*.

####### Distribution.

Estonia, Finland, Latvia, Lithuania, Sweden. Relatively rare. – Trans-Palearctic: from Europe and northern Africa to China and Japan (Linsenmaier 1997, Rosa et al. 2014). In eastern Asia represented by ssp. *extranea* Linsenmaier, 1959 (Rosa et al. 2014).

####### Biology.

Habitat: sparsely vegetated sandy areas, forest margins. Flight period: early June to late August. Adults are occasionally found on flowers of Apiaceae and Euphorbiaceae (Heinrich 1964, Linsenmaier 1997, Rosa 2006). Host: *Gymnomerus laevipes* (Shuckard), *Ancistrocerus* Wesmael and *Katamenes flavigularis* (Blüthgen) (Vespidae) (Martynova and Fateryga 2015), possibly also species of *Microdynerus* Thomson (Tischendorf 1998) and *Stenodynerus* Saussure (our own obs.) (Vespidae), based on their similar habitat preferences and body proportions with *Chrysis rutilans*. Older published host records of *Chrysis rutilans* may concern *Chrysis splendidula* and vice versa.

####### Remarks.

The status of the closely related species *Chrysis insperata* is still uncertain. It is difficult to separate from *Chrysis rutilans*, and Kunz (1994) and several subsequent authors consider *Chrysis insperata* as a *nomen dubium*. However, some authors (e.g. Linsenmaier 1959, 1997, Rosa 2006, Strumia 1995, 2005) considered both *Chrysis insperata* and *Chrysis rutilans* as valid species. Mitochondrial DNA sequences available at the Barcode of Life Data System (Ratnasingham and Hebert 2007) suggest that probably only one species is present in the Nordic countries.

##### *Chrysis viridula* group

###### Chrysis pulcherrima

Taxon classificationAnimaliaHymenopteraChrysididae

Lepeletier, 1806

Fig. 165

Chrysis pulcherrima Lepeletier, 1806: 127.

####### Diagnosis.

Length 6–8 mm. The species resembles *Chrysis viridula* by its colouration, but the head is dorsally partially red (not blue or green) in the female and occasionally also in the male. The mesosoma is dorsally red, as in *Chrysis viridula*, but the mesoscutum is medially green or blue in the male. The setae medially on the metatibia are shorter than the width of the tibia (Fig. 165) (not longer, as in *Chrysis viridula*).

####### Distribution.

Denmark. Very rare. Only one female specimen is known from the island of Lolland (Strandby, 2.VIII.1919, leg. L. Jørgensen). – West Palearctic: southern Europe (Linsenmaier 1959).

####### Biology.

Habitat: sparsely vegetated sandy areas. Flowers of Apiaceae are occasionally visited by adults (Mingo 1994, Rosa 2004). Flight period: June to August. Host: unknown. According to Linsenmaier (1959), one female (of ssp. *similitudina* Linsenmaier, 1959) was found in a colony of *Cerceris rubida* (Jurine) (Crabronidae).

###### Chrysis viridula

Taxon classificationAnimaliaHymenopteraChrysididae

Linnaeus, 1761

Figs 79 , 166

Chrysis viridula Linnaeus, 1761: 415.
Chrysis bidentata Linnaeus, 1767: 947.

####### Diagnosis.

Length 6–9 mm. The species is easy to recognise due to its distinctive colouration: the head, lateral and ventral parts of the mesosoma (including legs) and T3 are green, blue or violet, whereas the dorsal parts of the mesosoma, most of T1 and the entire T2 are red. The setae medially on the metatibia are longer than the tibial width (shorter in *Chrysis pulcherrima*). The apical teeth of T3 are often shallow and indistinct (Fig. 79).

####### Distribution.

Denmark, Estonia, Finland, Latvia, Lithuania, Norway, Sweden. Common. – Trans-Palearctic: from western Europe to Russian Far East, Korea, China and Japan (Linsenmaier 1997, Kurzenko and Lelej 2007, Rosa et al. 2014).

####### Biology.

Habitat: sparsely vegetated areas with clay or sandy soil, gardens. Adults occasionally visit flowers of Apiaceae (Linsenmaier 1997, Rosa 2004, our own obs.). Flight period: early June to late August. Host: *Odynerus spinipes* (Linnaeus), *Odynerus reniformis* (Gmelin) and *Odynerus melanocephalus* (Gmelin) (Vespidae) (Perez in Abeille de Perrin 1878, Adlerz 1910, Morgan 1984, our own obs.). The female oviposits in the host nest only when the host larva has completed its growth (Rosa 2006).

##### *Chrysis graelsii* group

###### Chrysis graelsii

Taxon classificationAnimaliaHymenopteraChrysididae

Guérin-Méneville, 1842

Chrysis Graelsii Guérin-Méneville, 1842: 148.
Chrysis Förster, 1853: 309.

####### Diagnosis.

Length 7–9 mm. The colouration is unique among North European chrysidids. The head and mesosoma are blue or greenish, and the mesoscutum is medially often dark blue or nearly black. The metasoma is dorsally red, but the apical rim of T3 is contrastingly blue.

####### Distribution.

Estonia, Finland, Latvia, Lithuania. Relatively rare. – West Palearctic: Europe and Asia Minor (Linsenmaier 1959, 1968).

####### Biology.

Habitat: forest margins, clearings and gardens with sun-exposed dead wood. Adults are occasionally found on flowers of Apiaceae and Euphorbiaceae (Poppius 1901, Heinrich 1964, Kunz 1994, Linsenmaier 1997). Flight period: late May to mid-August. Host: *Euodynerus notatus* (Jurine) (Vespidae) (Herrmann 1996, Pärn et al. 2014, our own obs.), probably also *Euodynerus quadrifasciatus* (Fabricius) (Saure 1998) and in Crimea *Euodynerus disconotatus* (Lichtenstein) (Martynova and Fateryga 2015).

##### *Chrysis ignita* group

###### Chrysis indigotea

Taxon classificationAnimaliaHymenopteraChrysididae

Dufour & Perris, 1840

Figs 82 , 156

Chrysis indigotea Dufour & Perris, 1840: 38.

####### Diagnosis.

Length 6–9 mm. The body is entirely dark blue or violet with greenish reflections. Usually the male is more greenish or lighter blue than the female. The species can be confused with *Chrysis iris*, but the mesoscutum is medially distinctly darker than laterally (Fig. 156), the punctation of the mesoscutum is denser and the ovipositor is broader (as in Fig. 91). Also the black spots of S2 are smaller and the apical teeth of T3 are more sharply produced (Fig. 82).

####### Distribution.

Sweden. Very rare. Only one specimen is known from Östergötland, collected probably in the late 1840s or early 1850s (leg. A.G. Dahlbom). – West Palearctic: central and southern Europe, northern Africa, Asia Minor (Linsenmaier 1959).

####### Biology.

Habitat: forest margins, clearings and gardens with sun-exposed dead wood (Trautmann 1927, 1930, Heinrich 1964, Brechtel 1986, Rosa 2006). Adults occasionally visit flowers of Apiaceae (Trautmann 1927, Linsenmaier 1997, Rosa 2006). Flight period: June to August. Host: possibly *Gymnomerus laevipes* (Shuckard) (Vespidae) and/or *Ectemnius rubicola* (Dufour and Perris) (Crabronidae) (Dufour and Perris 1840).

###### Chrysis fulgida

Taxon classificationAnimaliaHymenopteraChrysididae

Linnaeus, 1761

Fig. 93

Chrysis fulgida Linnaeus, 1761: 415.
Chrysis undata ? Dahlbom, 1831: 29.

####### Diagnosis.

Length 7–12 mm. The species differs from other North European chrysidids by its unique colouration. The head, mesosoma and T1 are dark blue or violet blue, whereas T2 and T3 are bright red (or rarely greenish) in the female. T1 often has golden reflections laterally. The male resembles the female in colouration, but T2 has a large dark blue or nearly black patch antero-dorsally with greenish margins (Fig. 93).

####### Distribution.

Denmark, Estonia, Finland, Latvia, Lithuania, Norway, Sweden. Common. – Trans-Palearctic: from western Europe to central Asia, Russian Far East and China (Linsenmaier 1997, Kurzenko and Lelej 2007, Rosa et al. 2014).

####### Biology.

Habitat: forest margins, clearings and gardens with sun-exposed dead wood. Adults are usually found flying near walls of wooden buildings, dead tree trunks (e.g. *Populus*, *Salix*, *Betula*, *Quercus*), log piles and poles. Occasionally they also visit flowers of Apiaceae (Rosa 2004, our own obs.). Flight period: mid-May to late August. Host: *Symmorphus allobrogus* (Saussure), *Symmorphus bifasciatus* (Linnaeus), *Symmorphus crassicornis* (Panzer) and *Symmorphus murarius* (Linnaeus) (Trautmann and Trautmann 1919, Wagner 1938, Pärn et al. 2014, Hopfenmüller 2015, our own obs.). Possibly also *Ancistrocerus parietum* (Linnaeus) (Vespidae) (Lamprecht 1881).

###### Chrysis iris

Taxon classificationAnimaliaHymenopteraChrysididae

Christ, 1791

Fig. 83

Chrysis iris Christ, 1791: 405.
Chrysis nitidula of authors, not Fabricius, 1775.
Chrysis soluta Dahlbom, 1854: 217.
Chrysis purpurata of authors, not Fabricius, 1787.

####### Diagnosis.

Length 7–13 mm. The body is mostly blue or blue-green, resembling *Chrysis indigotea* in colouration. The female often has green-golden reflections on the mesoscutum, mesoscutellum and anteriorly on the pronotum. The tergites are posteriorly lighter blue than anteriorly. Compared to *Chrysis indigotea*, the punctation of the mesoscutum is sparser and the interstices larger, the mesoscutum is medially not distinctly darker than laterally, and the ovipositor is narrower (as in Fig. 92). The black spots of S2 are also larger and the apical teeth of T3 shorter and blunter.

####### Distribution.

Denmark, Estonia, Finland, Latvia, Lithuania, Sweden. Relatively rare. – West Palearctic: Europe (Linsenmaier 1997).

####### Biology.

Habitat: forest margins, clearings and gardens with sun-exposed dead wood. Adults are usually found on walls of old log buildings (barns, sheds etc.), log piles, poles and dead tree trunks (e.g. *Populus*, *Salix*, *Betula*). Flight period: late May to late August. Host: *Symmorphus allobrogus* (Saussure), *Symmorphus crassicornis* (Panzer) and *Symmorphus murarius* (Linnaeus) (Vespidae) (Abeille de Perrin 1878, du Buysson 1895, Pärn et al. 2014, our own obs.).

###### Chrysis ruddii

Taxon classificationAnimaliaHymenopteraChrysididae

Shuckard, 1837

Fig. 96

Chrysis Ruddii Shuckard, 1837: 163.
Chrysis auripes Wesmael, 1839: 175.

####### Diagnosis.

Length 7–10 mm. As in most other species of the *Chrysis ignita* group, the head and mesosoma are mainly blue or green and the metasoma is dorsally golden red. However, the mesoscutum, mesoscutellum and propodeum, and often also tegulae and mesopleuron, have extensive golden or coppery reflections in the female. The sternites and legs are ventrally coppery red in both sexes. The punctation of the tergites is very fine and dense throughout, punctures being of uniform size (Fig. 96). In the male, the punctation is often somewhat sparser, and therefore it is more easily confused with other similarly coloured species of the *Chrysis ignita* group (e.g. *Chrysis subcoriacea*). The combination of a short pronotum (length less than one fourth of its width), non-metallic F1 and coppery red sternites should be used to distinguish *Chrysis ruddii* males reliably from other species of the group.

####### Distribution.

Denmark, Estonia, Finland, Latvia, Lithuania, Norway, Sweden. Relatively common. – West Palearctic: Europe, Asia Minor (Linsenmaier 1997).

####### Biology.

Habitat: dry meadows, rocky outcrops, cliffs, clay banks, forest margins. Adults occasionally visit flowers of Apiaceae and Euphorbiaceae (Heinrich 1964, Linsenmaier 1997, Rosa 2004, 2006). Flight period: mid-May to early August. Host: primarily *Ancistrocerus oviventris* (Wesmael) (Berland and Bernard 1938, Banaszak 1980, Morgan 1984, Kunz 1994, our own obs.), but possibly also *Ancistrocerus parietum* (Linnaeus), *Ancistrocerus scoticus* (Curtis), species of *Eumenes* Latreille, *Odynerus spinipes* (Linnaeus) and *Odynerus reniformis* (Gmelin) (Vespidae) (Forsius in Trautmann 1927, Berland and Bernard 1938, Banaszak 1980, Kunz 1994, Martynova and Fateryga 2015, our own obs.). Records stating solitary bees (e.g. *Hoplitis adunca* (Panzer) and *Hoplitis anthocopoides* (Schenck)) as hosts are doubtful, as bees differ significantly in their biology from the vespid hosts.

###### Chrysis corusca

Taxon classificationAnimaliaHymenopteraChrysididae

Valkeila, 1971

Figs 103 , 123 , 128 , 144 , 173

Chrysis corusca Valkeila, 1971: 84.

####### Diagnosis.

Length 7–9 mm. Both sexes are easily confused with e.g. *Chrysis angustula*, *Chrysis schencki*, *Chrysis impressa* and *Chrysis longula*, and a combination of several characters should always be used in identification. The vertex and mesosoma are mostly dark blue or blue-violet (rarely greenish), and often have green reflections on the pronotum and mesoscutellum. The punctures of the mesoscutum are of the same colour as the interstices. The metasoma is dorsally golden red or violet-red (Fig. 103) and S2 is greenish/bluish with narrow black spots (Figs 123, 128). The shape of the body is elongate and nearly parallel-sided (Fig. 103). Punctation of T2 is relatively regular and coarse (Fig. 103) and the mandible is thick (Fig. 144). The apical rim of T3 has a characteristic, widely arcuate interval between the central apical teeth.

####### Distribution.

Estonia, Finland, Latvia, Lithuania, Norway, Sweden. Relatively rare. – West Palearctic: central and northern Europe, Iran (van der Smissen 2010, Rosa et al. 2013).

####### Biology.

Habitat: forest margins, clearings and gardens with sun-exposed dead wood. Rarely also sandy banks and clay structures, such as old barn walls. Flight period: early June to early August. Host: *Symmorphus gracilis* (Brullé) (Vespidae) (Pärn et al. 2014).

###### Chrysis clarinicollis

Taxon classificationAnimaliaHymenopteraChrysididae

Linsenmaier, 1951

Figs 98 , 162

Chrysis ignita var. clarinicollis Linsenmaier, 1951: 77.
Chrysis clarinicollis : Schmid-Egger et al. 1995: 267.

####### Diagnosis.

Length 6–10 mm. The species is characterised by a uniformly green or blue-green pronotum and mesoscutellum, and a contrastingly darker blue or violet mesoscutum (Fig. 162). The tergites are mainly golden red, but T1 is anteriorly and laterally blue-green or green (Fig. 98). Shape of the metasoma is broad and compact (Fig. 98). Punctation of T2 is somewhat finer than on T1 and dense throughout (Fig. 98). The sternites and legs are ventrally greenish. The apical rim of T3 is narrow and the intervals of the apical teeth very shallow (Fig. 98). The mandible and ovipositor are thin (as in Figs 92 and 141). Males in particular can be confused with other species of the *Chrysis ignita* group as their colouration is more variable than in females. The colouration should always be used in combination with other characters (e.g. shape of metasoma and apical teeth) in species identification.

####### Distribution.

Estonia. Very rare. Only four records are known from Estonia, all from the western part of the country (Ahu, Manilaid, Tuudi, Väike-Pakri; 2001–2009). – West Palearctic: southern and central Europe, northern Africa (Linsenmaier 1997).

####### Biology.

Habitat: forest margins, clearings and gardens with sun-exposed dead wood. Usually observed on dead tree trunks and on walls of abandoned houses (Rosa 2006), but also flying near the ground and over rocks (Linsenmaier 1997). Flight period: June to August. The earliest Estonian specimens were collected in the beginning of June and the latest in the end of July. Host: possibly *Ancistrocerus oviventris* (Wesmael) and/or *Ancistrocerus scoticus* (Curtis) (Vespidae) (Petit 1987).

###### Chrysis vanlithi

Taxon classificationAnimaliaHymenopteraChrysididae

Linsenmaier, 1959

Figs 97 , 115 , 140 , 159

Chrysis rutiliventris ssp. vanlithi Linsenmaier, 1959: 153.
Chrysis rutiliventris of authors, not Abeille de Perrin, 1879.
Chrysis vanlithi : Soon et al. 2014: 305.

####### Diagnosis.

Length 7–10 mm. The species is easily confused with other similarly coloured species of the *Chrysis ignita* group (e.g. *Chrysis borealis* sp. n. and *Chrysis schencki*), and the males in particular can be difficult to identify. The combination of several characters (e.g. shape of pronotum and malar space and colouration) should always be used in species determination. The head and mesosoma are dorsally dark blue or nearly black with light blue or greenish reflections mainly on the pronotum (Fig. 159). The tergites are golden red (Fig. 97) and the sternites and legs ventrally greenish (Fig. 115). The pronotum is short (length not more than one fourth of width) (Fig. 159). The mandible is relatively thin (Fig. 140) and the malar space long, approximately as long as broad in the female (Fig. 140). F1 is black without a metallic sheen, and the ovipositor is narrow (as in Fig. 92).

####### Distribution.

Denmark, Norway, Sweden. Rare. Only one confirmed record is known from Denmark, four from southern Sweden (Scania, Bohuslän and Stockholm archipelago) and ten from southern Norway. – West Palearctic: from central and northern Europe to southwestern Asia (Linsenmaier 1997).

####### Biology.

Habitat: all Nordic specimens have been found in coastal localities. Adults are usually found flying near rocks and log walls (Linsenmaier 1997, our own obs.). Flight period: June to July (most Nordic observations are from June). Host: possibly *Ancistrocerus gazella* (Panzer) (our own obs.) or *Ancistrocerus scoticus* (Curtis) (Vespidae) (van der Smissen 2001).

###### Chrysis subcoriacea

Taxon classificationAnimaliaHymenopteraChrysididae

Linsenmaier, 1959

Figs 87 , 109 , 117 , 129 , 146 , 160 , 174

Chrysis longula ssp. subcoriacea Linsenmaier, 1959: 160.
Chrysis subcoriacea : Valkeila 1962: 64.

####### Diagnosis.

Length 9–13 mm. Females are usually easy to recognise by the laterally coriaceous and dull T2 and T3 (Fig. 109). Males, however, are often confused with *Chrysis impressa* and *Chrysis longula*. Compared to *Chrysis impressa*, the sternites are usually brighter red and the black spots of S2 larger (Fig. 129), the mandible thicker (Fig. 146) and the transition of the lateral margin of T3 into the lateral apical teeth is straighter (not concave) (Fig. 87). Compared to *Chrysis longula* (Fig. 161), the middle part of the pronotum is not as sharply darker than the margins (Fig. 160), the lateral margins of T3 are straighter, the punctation of T2 is usually finer anteriorly, and the central interval of the apical teeth is more angled (Fig. 87). In both sexes, the head and mesosoma are dorsally dark blue or nearly black with light blue or greenish reflections mainly on the pronotum. The tergites and sternites are red. The body is elongate and often rather large.

####### Distribution.

Denmark, Estonia, Finland, Latvia, Norway, Sweden. Relatively rare. – Trans-Palearctic: from Europe to central Asia and Japan (Linsenmaier 1997).

####### Biology.

Habitat: forest margins, clearings and gardens with sun-exposed dead wood. Adults often fly near dead tree trunks (*Betula*, *Populus*, *Salix*) and close to walls of wooden buildings (log barns, sheds etc.), and they are also attracted to honeydew of aphids. Flight period: late May to late August. Host: possibly *Ancistrocerus trifasciatus* (Müller) (Vespidae) (our own obs.).

###### Chrysis angustula

Taxon classificationAnimaliaHymenopteraChrysididae

Schenck, 1856

Figs 86 , 99 , 119 , 126

Chrysis angustula Schenck, 1856: 28.
Chrysis gracilis ? Schenck, 1856: 30.
Chrysis brevidens Tournier, 1879: 96.

####### Diagnosis.

Length 6–9 mm. The species can be confused with several other similarly coloured species of the *Chrysis ignita* group (e.g. *Chrysis leptomandibularis*, *Chrysis schencki* and *Chrysis corusca*). Compared to *Chrysis leptomandibularis* and *Chrysis schencki*, the mandible is thicker, the punctation of T2 is finer, the black spots of S2 are more rectangular and the posterior margin of the propodeal tooth is directed more downward. Compared to *Chrysis corusca*, the mandible is thinner, the punctation of the tergites is finer and the colour of the sternites is more reddish (not green). The head and mesosoma are mainly dark blue, and usually have extensive green or golden reflections on the frons, pronotum, mesopleuron, mesoscutum and mesoscutellum, especially in the female. The metasomal tergites are golden red (Fig. 99) and the sternites golden or reddish (Figs 119, 126). The black spots of S2 are characteristically rectangular in shape (Figs 119, 126). The body is elongate and slender, with parallel sides (resembling *Chrysis leptomandibularis*) (Fig. 99). The punctation of T2 is fine, and its surface is strongly shining posteriorly (Fig. 99). T3 is relatively long, and strongly shining, especially in the female (Fig. 99). The apical teeth are short, and the central interval is wide and shallow (Figs 86, 99). The ovipositor is narrow (as in Fig. 92) and the mandible relatively thick in both sexes.

####### Distribution.

Denmark, Estonia, Finland, Latvia, Lithuania, Norway, Sweden. Very common. – Trans-Palearctic. Europe, southwestern Asia, Siberia, China (Linsenmaier 1997, Rosa et al. 2014).

####### Biology.

Habitat: forest margins, clearings and gardens with sun-exposed dead wood. Adults are often found on walls of wooden buildings, poles, log piles and dead tree trunks. Occasionally they also visit flowers of Apiaceae and Asteraceae (Banaszak and Kochanowski 1994, our own obs.) and are attracted to honeydew of aphids. Flight period: late May to September. Host: primarily *Symmorphus bifasciatus* (Linnaeus) (van Lith 1958, Niehuis 2000, Pärn et al. 2014, our own obs.), but occasionally also *Ancistrocerus trifasciatus* (Müller), *Symmorphus allobrogus* (Saussure), *Symmorphus connexus* (Curtis) and *Symmorphus debilitatus* (Saussure) (Vespidae) (van Lith 1958, Niehuis 2000, Pärn et al. 2014, our own obs.).

###### Chrysis longula

Taxon classificationAnimaliaHymenopteraChrysididae

Abeille de Perrin, 1879

Figs 6 , 88 , 102 , 121 , 161

Chrysis ignita var. longula Abeille de Perrin, 1879: 74.
Chrysis longula var. sublongula Linsenmaier, 1951: 76.
Chrysis longula ssp. aeneopaca Linsenmaier, 1959: 160.
Chrysis longula : Linsenmaier 1959: 159.

####### Diagnosis.

Length 10–13 mm. The body is elongate with parallel sides (Fig. 102) and usually large compared to other species of the *Chrysis ignita* group. The head and mesosoma are dorsally blue or black, and the female has extensive golden green reflections on the pronotum, mesopleuron and mesoscutellum. The punctures of the mesoscutum are usually lighter coloured than the interstices (as in *Chrysis impressa*). The tergites and sternites are golden red (Figs 102, 121) and the black spots of S2 are long and narrow (Fig. 121). The punctation of T2 is anteriorly very coarse, and the surface of T3 is shiny in the female (Fig. 102). The mandible is long and relatively thick (in the male as in Fig. 146). Small specimens can be confused with *Chrysis angustula* and *Chrysis corusca*, but the punctation of T2 is coarser anteriorly and the black spots of S2 are narrower. The sternites are mostly red, not greenish as in *Chrysis corusca*. The males are often difficult to distinguish from *Chrysis impressa* and *Chrysis subcoriacea*. Compared to *Chrysis impressa*, the sternites are usually brighter red and the black spots of S2 are more elongate. The punctation of T2 is coarser anteriorly and the metasoma is more elongate in shape. Compared to *Chrysis subcoriacea*, the pronotum is more abruptly bicoloured (Fig. 161), the punctation of T2 is usually coarser anteriorly, the lateral margin of T3 is more concave and the central interval of the apical teeth is more arcuate (Fig. 88).

####### Distribution.

Denmark, Estonia, Finland, Latvia, Lithuania, Norway, Sweden. Relatively rare. – Trans-Palearctic: from western Europe to central Asia, Siberia and China (Linsenmaier 1959, 1997, Rosa et al. 2014).

####### Biology.

Habitat: forest margins, clearings and gardens with sun-exposed dead wood. Adults can be found on walls of old log buildings (barns, sheds etc.), log piles, poles and dead tree trunks (e.g. *Betula*, *Populus*, *Salix*). Flight period: early June to late August. Host: *Ancistrocerus antilope* (Panzer), *Symmorphus crassicornis* (Panzer) and *Symmorphus murarius* (Linnaeus) (Linsenmaier 1959, Heinrich 1964, Morgan 1984, Brechtel 1985, Petit 1987, Martynova and Fateryga 2015), possibly also *Ancistrocerus parietinus* (Linnaeus) and species of *Euodynerus* Dalla Torre (Vespidae) (Blüthgen 1961, Morgan 1984, Martynova and Fateryga 2015).

####### Remarks.

Eastern Palearctic populations of Chrysis longula belong to ssp. aeneopaca Linsenmaier, 1959 which differs from the nominotypical subspecies by having fine punctation and brownish colour anteriorly on tergites. Specimens of ssp. *aeneopaca* can sometimes be confused with *Chrysis subcoriacea*. One specimen similar to ssp. *aeneopaca* has been found in Finland (Kuopio), but the occurrence of the taxon in Fennoscandia is questionable (Paukkunen et al. 2014).

###### Chrysis brevitarsis

Taxon classificationAnimaliaHymenopteraChrysididae

Thomson, 1870

Figs 155 , 157 , 172

Chrysis brevitarsis Thomson, 1870: 107.

####### Diagnosis.

Length 7–10 mm. The species is characterised by its subapically toothed mandible (Fig. 155), which is unique among the Nordic and Baltic species of the *Chrysis ignita* group. The head and mesosoma are mainly dark blue with greenish reflections, and the metasoma is dorsally golden red or dark red. As in *Chrysis pseudobrevitarsis*, the spurs of the mesotibia are approximately equal in length (Fig. 167) and the mandible is very thick (medial width more than half of its basal width in the female and more than two thirds of the basal width in the male). In the female, the metatarsus is not longer than the metatibia (Fig. 169) and the antenna is strongly nodular (Fig. 172). The punctation of T2 and the mesoscutum (Fig. 157) is sparser compared to *Chrysis pseudobrevitarsis*.

####### Distribution.

Estonia, Finland, Lithuania, Sweden. Rare. – West Palearctic: northern and central Europe (Linsenmaier 1997).

####### Biology.

Habitat: forest margins, clearings and gardens with sun-exposed dead wood (usually *Betula*, *Populus* and/or *Alnus*). Flight period: June to July. Host: *Discoelius dufourii* Lepeletier and *Discoelius zonalis* (Panzer) (Vespidae) (Blüthgen 1961, Kunz 1994, our own obs.), possibly also *Ancistrocerus antilope* (Panzer) (Vespidae) (Martynova and Fateryga 2015).

###### Chrysis pseudobrevitarsis

Taxon classificationAnimaliaHymenopteraChrysididae

Linsenmaier, 1951

Figs 167 , 169

Chrysis ignita var. pseudobrevitarsis Linsenmaier, 1951: 79.
Chrysis pseudobrevitarsis : Linsenmaier 1959: 158.

####### Diagnosis.

Length 6–10 mm. The colouration and habitus are similar to *Chrysis brevitarsis*, but the mandible does not have a subapical tooth, the punctation of the mesoscutum is laterally denser and the punctation of T2 is usually coarser. The short metatarsus (Fig. 169) is characteristic for the females of both species. Males can be confused with e.g. *Chrysis longula* and *Chrysis impressa*, but the spurs of the mesotibia are approximately equal in length (Fig. 169), the shape of the body is more compact, and the inner margin of the paramere is angled (as in Fig. 137), not rounded.

####### Distribution.

Denmark, Estonia, Finland, Latvia, Lithuania, Norway, Sweden. Relatively rare. – Trans-Palearctic: from western Europe to Mongolia (Linsenmaier 1997).

####### Biology.

Habitat: forest margins, clearings and gardens with sun-exposed dead wood. Adults occasionally visit flowers of Apiaceae (Petit 1987). Flight period: late May to late August. Host: primarily *Euodynerus notatus* (Jurine) (Pärn et al. 2014, our own obs.), but probably also *Euodynerus quadrifasciatus* (Fabricius) and *Ancistrocerus antilope* (Panzer) (Vespidae) (Heinrich 1964, Morgan 1984, Martynova and Fateryga 2015).

###### Chrysis mediata

Taxon classificationAnimaliaHymenopteraChrysididae

Linsenmaier, 1951

Fig. 100

Chrysis ignita var. mediata Linsenmaier, 1951: 76.
Chrysis mediata : Linsenmaier 1959: 154.

####### Diagnosis.

Length 6–10 mm. *Chrysis mediata* and *Chrysis solida* are closely related sibling species, which differ from other similarly coloured species of the *Chrysis ignita* group by the combination of the following characters: 1) the ovipositor is broad (Figs 91, 100), 2) the inner margin of the paramere is angled (Fig. 137) and 3) the spurs of the mesotibia are of unequal length (as in Fig. 168). Both species are also characterised by the greenish sternites, the rounded or slightly rectangular black spots of S2 (Figs 120, 125), the relatively thick mandible, and the fine and dense punctation of T2, especially in the female (Figs 100, 101). *Chrysis mediata* is often very difficult to differentiate from *Chrysis solida*, but generally the body is larger and broader (Fig. 100), the head is narrower in frontal view (only slightly broader than high), the surface of T3 is shinier and the colour of the head and mesosoma is predominantly lighter blue. The colour of the tergites is golden red or dark red (Fig. 100), as in *Chrysis solida*. The hosts of *Chrysis mediata* are soil-nesting (terricolous) species of the genus *Odynerus* Latreille, whereas in *Chrysis solida* the hosts are cavity-nesting (xylicolous) species, mainly *Ancistrocerus* Wesmael.

####### Distribution.

Estonia, Latvia, Lithuania, Sweden. Relatively rare. – West Palearctic? Reliable distributional data from the eastern Palaearctic are not available. Records from Japan have been found to be erroneous (Linsenmaier 1997).

####### Biology.

Habitat: sun-exposed clay and loess walls and banks, sand pits, road verges and meadows. Adults have been found on flowers of Euphorbiaceae (Rosa 2004). Flight period: early June to late July. Host: primarily *Odynerus spinipes* (Linnaeus), but also *Odynerus reniformis* (Gmelin) (Vespidae) (van Lith 1958, Linsenmaier 1959, Banaszak 1980, Morgan 1984).

###### Chrysis solida

Taxon classificationAnimaliaHymenopteraChrysididae

Haupt, 1957

Figs 91 , 101 , 120 , 125 , 137

Chrysis ignita ssp. solida Haupt, 1957: 115.
Chrysis mediata ssp. fenniensis Linsenmaier, 1959: 154.
Chrysis mediata of authors, not Linsenmaier, 1951.
Chrysis scintillans Valkeila, 1971: 85.
Chrysis solida : Niehuis 2001: 120.

####### Diagnosis.

Length 5–9 mm. The species is closely related to *Chrysis mediata*, but the body is usually smaller and with more parallel sides (Fig. 101), the head is broader in frontal view (distinctly broader than its height), the punctation of T2 is often somewhat denser anteriorly (Fig. 101), the surface of T3 is not as shiny (Figs 101) and the colouration is predominantly darker. The species can be confused also with e.g. *Chrysis schencki*, *Chrysis angustula* and *Chrysis corusca*. However, the ovipositor is broader (Fig. 91) and the inner margin of paramere is angled (Fig. 137) (not rounded). Also the green-blue colouration of S2 and the rounded shape of the black spots (Fig. 120, 125) are characteristic for *Chrysis solida*.

####### Distribution.

Denmark, Estonia, Finland, Latvia, Lithuania, Norway, Sweden. Common. – Trans-Palearctic: from western Europe to Japan (Linsenmaier 1997).

####### Biology.

Habitat: forest margins, clearings and gardens with sun-exposed dead wood. Adults fly near walls of buildings (log barns, sheds etc.), dead tree trunks (e.g. *Betula*, *Populus*, *Quercus*, *Salix*), log piles and poles. They rarely visit flowers of Apiaceae (our own obs.). Flight period: late May to early September. Host: primarily *Ancistrocerus trifasciatus* (Müller), but occasionally also *Euodynerus notatus* (Jurine) and possibly *Symmorphus debilitatus* (Saussure) (Vespidae) (Pärn et al. 2014).

####### Remarks.

*Chrysis solida* and *Chrysis mediata* are very similar morphologically and genetically despite clear differences in their biology and host selection (Soon et al. 2014). Reliable species identification is not always possible without information on the host or habitat.

###### Chrysis leptomandibularis

Taxon classificationAnimaliaHymenopteraChrysididae

Niehuis, 2000

Figs 118 , 127 , 142 , 145

Chrysis leptomandibularis Niehuis, 2000: 192.

####### Diagnosis.

Length 5–8 mm. The size and shape of the body are similarly slender and elongate as in *Chrysis angustula*. However, the flagellomeres are shorter, S2 is greenish (not reddish) with shorter black spots (Figs 118, 127) and the mesoscutum has wider, strongly shining interstices between the punctures in the female. The mandible is extremely thin in the female (medial width less than one third of its basal width) (Fig. 142) and somewhat thicker in the male (medial width about one third of its basal width) (Fig. 145). Compared to *Chrysis schencki*, the mandible is thinner, the punctation of the mesoscutum is sparser and the body is more slender.

####### Distribution.

Estonia, Finland, Latvia, Lithuania, Norway. Rare. Only one old record is known from Finland. New to Norway (1 ♀, Østfold, Aremark, Teigen, 59.254°N, 11.644°E, 9.VIII.2015, leg. F. Ødegaard). – West Palearctic: central and northern Europe (Niehuis 2000).

####### Biology.

Habitat: forest margins, clearings and gardens with sun-exposed dead wood. Adults have occasionally been found on flowers of Apiaceae and Rosaceae (Rosa 2006, our own obs.). Flight period: June to August. Host: probably *Symmorphus debilitatus* (Saussure) (Vespidae) (Niehuis 2000, Pärn et al. 2014).

###### Chrysis schencki

Taxon classificationAnimaliaHymenopteraChrysididae

Linsenmaier, 1968

Figs 131 , 143 , 148 , 176

Chrysis ignita ssp. schenckiana Linsenmaier, 1959: 156, not Mocsáry, 1912.
Chrysis ignita ssp. schencki Linsenmaier, 1968: 99, replacement name for *schenckiana* Linsenmaier, 1959.
Chrysis schencki : Erlandsson 1971: 88.

####### Diagnosis.

Length 6–10 mm. The head and mesosoma are dorsally dark blue, violet or nearly black, and the punctures of the mesoscutum are usually of the same colour as the interstices. The tergites are golden red and the sternites variably golden or greenish (Fig. 131). The punctation of T3 is often characteristically dense and homogeneous. The body shape is more elongate than in *Chrysis ignita*, *Chrysis impressa* and *Chrysis borealis* sp. n., but not as slender as in *Chrysis angustula* and *Chrysis leptomandibularis*. Females are usually best distinguished from other species of the *Chrysis ignita* group by their thin and basally concave mandible (Fig. 143), though males are more difficult to identify. Compared to *Chrysis impressa* and *Chrysis borealis* sp. n., the mandible of male is slightly thinner and basally more concave (Fig. 148), the body is more slender, and the relative length of F1 to F2 is somewhat smaller (Fig. 176). Identification of the males is not always possible with certainty by morphological characters alone.

####### Distribution.

Estonia, Finland, Latvia, Lithuania, Norway, Sweden. Common. – Trans-Palearctic: from western Europe to central Asia, Siberia and Japan (Linsenmaier 1997).

####### Biology.

Habitat: forest margins, clearings and gardens with sun-exposed dead wood. Adults fly near sun-exposed dead tree trunks (e.g. *Betula*, *Populus*, *Quercus*), and near walls of wooden buildings (e.g. log barns, sheds), poles and log piles. Flight period: late May to September. Host: *Ancistrocerus trifasciatus* (Müller) (Vespidae) (Pärn et al. 2014, our own obs.). Possibly also *Ancistrocerus gazella* (Panzer) and *Ancistrocerus nigricornis* (Curtis) (Schneider 1991).

####### Remarks.

Recent mitochondrial DNA studies have shown that *Chrysis schencki* consists of two distinct and sympatric genetic lineages in northern Europe (Soon et al. 2014). It is possible that they represent two different species, but more morphological and molecular studies are needed.

###### Chrysis ignita

Taxon classificationAnimaliaHymenopteraChrysididae

(Linnaeus, 1758)

Figs 104 , 105 , 122 , 130 , 147 , 163 , 175

Sphex ignita Linnaeus, 1758: 571.
Chrysis ignita : Linnaeus 1761: 414.
Chrysis ignita form B sensu Linsenmaier 1959: 156.

####### Diagnosis.

Length 5–10 mm. *Chrysis ignita* resembles closely *Chrysis terminata* in colouration, structure and habitus, but the frontal carina is shallowly M-shaped or more or less arcuate (not forming four tooth-like tubercles). The head and mesosoma are dorsally shiny blue or violet with green reflections on the pronotum and mesoscutellum (Fig. 163). The punctures of the mesoscutum are of the same colour as the interstices (Fig. 163) (not lighter as in *Chrysis impressa*). The tergites are golden red (Figs 104, 105), the sternites green or blue (Figs 122, 130) and the black spots of S2 are subrectangular in shape (Figs 120, 130). The punctation of T2 and T3 is coarse and regular (Figs 104, 105) and the apical teeth are sharply produced (Figs 104, 105). The medial furrow of the pronotum is narrow (Fig. 163) and the pubescence of the vertex is white (Fig. 147) or sometimes brown in the male.

####### Distribution.

Denmark, Estonia, Finland, Latvia, Lithuania, Norway, Sweden. Relatively rare. – West Palearctic: from western Europe to central Asia and China (Linsenmaier 1997, Rosa et al. 2014).

####### Biology.

Habitat: gardens, parks and forest margins. Adults are usually collected from walls of old buildings (both wooden and stone), dead tree trunks, poles and log piles. They rarely visit flowers of Apiaceae (Rosa 2004). Flight period: late May to early September. Host: probably *Ancistrocerus parietum* (Linnaeus) (Vespidae) (our own obs.). Numerous host records have been published for *Chrysis ignita*, but most of these are unreliable, due to inconsistent taxonomic treatment of the species.

####### Remarks.

A few studied specimens from Norway, Finland and Lithuania differ significantly from other North European *Chrysis ignita* specimens based on their mitochondrial DNA sequences. According to Soon et al. (2014), they could represent a cryptic species (“*Chrysis* sp.1”). No distinct morphological differences have been found between the two North European genetic forms.

###### Chrysis impressa

Taxon classificationAnimaliaHymenopteraChrysididae

Schenck, 1856

Figs 10 , 84 , 85 , 92 , 107 , 108 , 116 , 132 , 149 , 164 , 168 , 170 , 177

Chrysis impressa Schenck, 1856: 29.
Chrysis ignita var. aurifera Linsenmaier, 1951: 76.

####### Diagnosis.

Length 6–11 mm. The species is easily confused with other similarly coloured species of the *Chrysis ignita* group and a combination of different diagnostic characters should be used in species determination. The head and the mesosoma are dorsally dark blue or black, and in the female the pronotum, mesopleuron and mesoscutellum have extensive golden green reflections (Fig. 164). The mesoscutum of the female is characteristically black, dark grey or olive coloured with contrastingly green or blue punctures (similar to *Chrysis longula*) (Fig. 164). The mesoscutum of the male is often entirely dark blue or blue-violet. The tergites are golden red and relatively finely punctured (Figs 107, 108). The sternites are at least partially red-golden (Figs 116, 132) and the black spots of S2 are usually roundish in the female (Figs 116). The setae on the dorsal surface of the head are brownish in both sexes (Fig. 149). The mandible is relatively thick (medial width about half or nearly half of basal width) and basally with only slightly concave margins (Figs 141, 149). F1 is long and narrow, about 1.4 times as long as F2 in the female and at least 1.2 times as long as F2 in the male (Fig. 177).

####### Distribution.

Denmark, Estonia, Finland, Latvia, Lithuania, Norway, Sweden. Very common. – West Palearctic: from western Europe to central Asia (Linsenmaier 1997).

####### Biology.

Habitat: forest margins, clearings and gardens with sun-exposed dead wood. Adults are mainly observed flying and running on walls of wooden buildings (e.g. log barns), dead tree trunks (e.g. *Betula*, *Populus*), poles and log piles. Flight period: early June to late August. Host: Mainly *Ancistrocerus claripennis* Thomson and *Ancistrocerus parietinus* (Linnaeus) (Pärn et al. 2014, Martynova and Fateryga 2015, our own obs.), but probably also *Ancistrocerus trifasciatus* (Müller) (Vespidae) (Morgan 1984, Pärn et al. 2014, our own obs.).

###### Chrysis borealis

Taxon classificationAnimaliaHymenopteraChrysididae

Paukkunen, Ødegaard & Soon sp. n.

http://zoobank.org/3DDF3EF4-6694-4586-B78E-FF79E321AB92

Figs 106 , 124 , 133 , 136 , 158 , 178 , 179 , 180–186 , 187–195

Chrysis mediadentata of authors, not Linsenmaier, 1951.
Chrysis sp. sensu Soon et al. (2014) and Paukkunen et al. (2014: 44).

####### Type material.

11 ♀♀ and 15 ♂♂. DNA barcode sequences of all type specimens are available at GenBank or Barcode of Life Data System (Ratnasingham and Hebert 2007).

####### Holotype.

**Figure 179.**
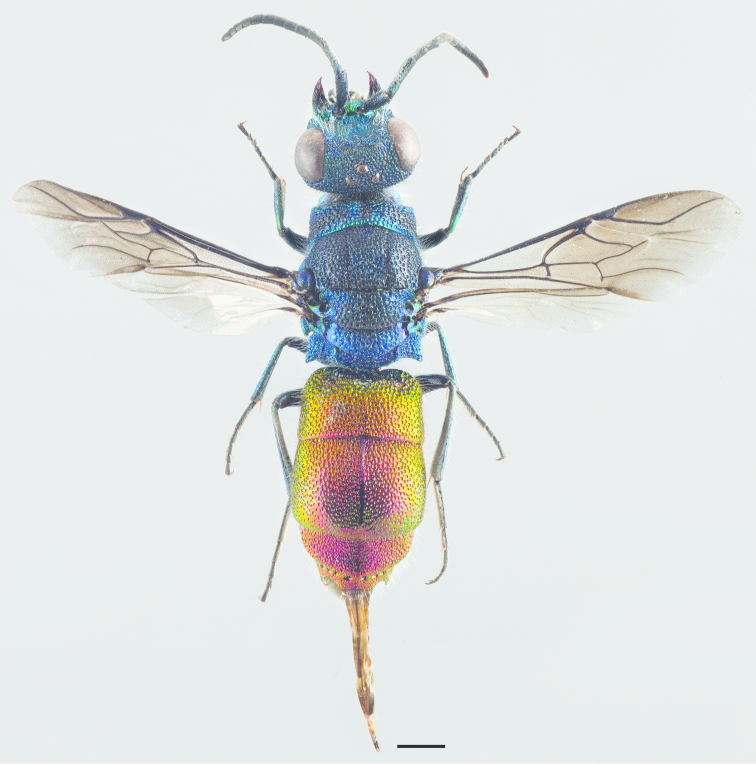
Holotype of *Chrysis borealis* sp. n. ♀ (HYMNI560) Collected from Ørin, Nord-Trøndelag, Norway. Scale 1 mm. (Photo: Arnstein Staverløkk).

**Norway**, ♀ (HYMNI560), Nord-Trøndelag, Ørin, 63.802°N, 11.459°E, 9.VII.2014, leg. F. Ødegaard (NUM) (Fig. 179).

####### Paratypes.

**Finland**, 1 ♂ (FACU-000396), Kittilän Lappi, Kolari, Ylläs, 67.586°N, 24.239°E, 9.VII.1989, leg. M. Koponen (MZH); 1 ♂ (MZH_GP.74723), Sompion Lappi, Sodankylä, 68.027°N, 27.413°E, 1.VII.2002, leg. J. Itämies (MZH); 1 ♀ (TUZ616001), Enontekiön Lappi, Malla Strict Nature Reserve, 69.060°N, 20.759°E, 15.VII.2009, leg. V. Soon (TUZ); 1 ♀ (FACU-000399), Enontekiön Lappi, Annjaloanji, 69.172°N, 21.439°E, 11.VII.2007, leg. R. Jussila (private collection of M. Raekunnas, Hämeenlinna, Finland); 2 ♂♂ (MZH_GP.92704, MZH_GP.92705), Inarin Lappi, Inari, Ivalo, 68.643°N, 27.524°E, 26.VI.2013, leg. T. Järveläinen (MZH and NMLS); **Norway**, 1 ♂ (Chrysis132), Buskerud, Hokksund, Lilleby, 59.779°, N 9.933°E, 1.V.2012, leg. F. Ødegaard (NUM); 1 ♂ (Chrysis004), Buskerud, Nedre Eiker, Solbergfjell, 59.759°N, 10.041°E, 28.VI.2012, leg. F. Ødegaard (NUM); 1 ♀ (NOCHR254), Hordaland, Masfjorden, Mjanger, 60.768°N, 5.348°E, 3.IX.2009, leg. A. Staverløkk (NUM); 1 ♂ (HYMNI559), Nord-Trøndelag, Ørin, 63.802°N, 11.459°E, 9.VII.2014, leg. F. Ødegaard (NUM); 1 ♂ (Chrysis034), Oppland, Nord-Fron, Stordalsberget, 61.587°N, 9.819°E, 9.V.2009, leg. F. Ødegaard (NUM); 1 ♂ (Chrysis089), Oppland, Nord-Fron, Stordalsberget, 61.587°N, 9.819°E, 1.VI.2009, leg. F. Ødegaard (NUM); 1 ♀ (Chrysis180), Oppland, Nord-Fron, Stordalsberget, 61.587°N, 9.819°E, 1.IX.2009, leg. F. Ødegaard (NUM); 1 ♂ (Chrysis183), Sogn og Fjordane, Luster, Ornes, 61.286°N, 7.341°E, 4.VII.2011, leg. F. Ødegaard (NUM); 1 ♂ (Chrysis021), Sør-Trøndelag, Røros, Småsetran, 62.573°N, 11.413°E, 11.VII.2010, leg. F. Ødegaard (NUM); 1 ♀ (Chrysis187), 1 ♂ (Chrysis189), Sør-Trøndelag, Røros, Småsetran, 62.573°N, 11.413°E, 23.VII.2007, leg. F. Ødegaard (NUM); 1 ♂ (Chrysis125), Sør-Trøndelag, Røros, Kvitsanden, 62.573°N, 11.412°E, 31.VII.2008, leg. F. Ødegaard (NUM); 1 ♂ (NOCHR267), Sør-Trøndelag, Trondheim, Lade, 63.447°N, 10.434°E, 27.V.2013, leg. F. Ødegaard (NUM); **Russia**, 1 ♀ (MZH_GP.78002), Lapponia tulomensis, 45 km east of Murmansk, 68.876°N, 34.196°E, 16.VII.2006, leg. M.V. Kozlov (MZH); **Sweden**, 1 ♀ (TUZ616002), Öland, Persnäs, 57.046°N, 16.931°E, 20.VII.2007, leg. J. Abenius (TUZ); 1 ♀ (MZH_GP.92690), Gotland, Stora Karlsö, Hien, 57.289°N, 17.964°E, 6.VII.2012, leg. N. Johansson (MZH); 1 ♀ (MZH_GP.92688), 1 ♂ (MZH_GP.92689), Gotland, Stora Karlsö, Hien, 57.289°N, 17.964°E, 8.VII.2012, leg. N. Johansson (MZH); 1 ♀ (MZH_GP.92691), Gotland, Fårö, Norsta Auren, 57.981°N, 19.326°E, 8.VII.2012, leg. N. Johansson (MZH).

####### Diagnosis.

Length 6–11 mm. The species is very similar to *Chrysis impressa* in shape and structure, but the colouration is darker and the length of F1 compared to F2 is larger (Table 1). The mesoscutum of the female is usually black, violet or dark blue with relatively fine and dense punctation (Figs 158, 179). The punctures are generally of the same colour as the interstices. The metasoma has golden red or reddish tergites (Figs 106, 179) and the sternites are dark green or bluish in the female (Fig. 124), but often with golden red colour in male (Fig. 133). Compared to *Chrysis corusca*, the body shape is stouter, the metasoma is notably swollen (Figs 106, 179), the black spots of S2 are broader (Figs 124, 133) and the mandible is thinner. Dark specimens of *Chrysis schencki* are also very similar, but have a thinner mandible and coarser punctation on the scapal basin, and are more slender in habitus. The males of *Chrysis borealis* in particular are difficult to distinguish from *Chrysis impressa* and *Chrysis schencki*. On average, the length ratio F1/F2 is larger (1.3–1.5:1) (Fig. 178, Table 1), the black spots of S2 are larger (Fig. 133) and the punctation of the mesoscutum is finer in *Chrysis borealis*. Identification of the males is not always possible with certainty.

####### Description of female.

Body length 7.8–10.3 mm, forewing length 5.1–6.6 mm (n = 12).

Head. Height 1.8–2.1 mm, width 2.3–2.6 mm, length 1.0–1.1 mm, shortest interocular distance 9.4–11.0 mm. Scapal basin green or greenish blue, usually becoming darker blue or violet dorsally below frontal carina. Punctation of scapal basin very dense and fine, partially coriaceous with rugae formed by the puncture margins. Transverse frontal carina well developed, usually relatively evenly arcuate or slightly notched medially. Vertex dark blue, dark violet or black. Pubescence on vertex light brownish. Malar space 1.4 times broader than high. Mandible blackish brown, apically and in inner margin light brown, without subapical tooth. In lateral view, mandible relatively thick (similar to *Chrysis impressa*), its sides medially almost parallel and basally only slightly concave. Scapus, pedicellus, and F1 with green, blue or violet metallic reflections. Relative lengths of P/F1/F2/F3 are 1/1.8/1.1/0.9. F1 usually 1.4–1.7 times as long as F2 (Table 1). F4–F10 approximately 1.2 times as long as broad.

Mesosoma. Length 3.0–3.8 mm, width anterior to tegulae 2.0–2.7 mm. Length of pronotum medially 0.5–0.6 mm and width at anterior margin 1.8–2.2 mm. Colour of pronotum medially black, dark violet or dark blue, on the margins lighter green, blue or violet, only rarely with golden reflections (Figs 158, 179). Medial groove relatively shallow and indistinctly delimited. Mesoscutum dark violet or black, sometimes with bluish reflections (Figs 158, 179). Punctures generally of the same colour as interstices. Punctation of mesoscutum relatively fine and dense with narrow interstices (Figs 158, 179). Size of punctures on average smaller than in *Chrysis impressa* and *Chrysis schencki*. Interstices with scattered small punctures. Tegula green, blue or violet, with paler colour laterally. Mesoscutellum black medially and violet, blue, or sometimes greenish laterally, with irregular large punctures. Metanotum and propodeum violet, blue or greenish. Outer margin of lateral propodeal teeth straight or slightly concave. Pubescence on mesosoma whitish. Legs violet, blue or greenish, but tarsal segments dark brown. Wing venation as in *Chrysis impressa* and *Chrysis schencki*.

**Figures 180–186.**
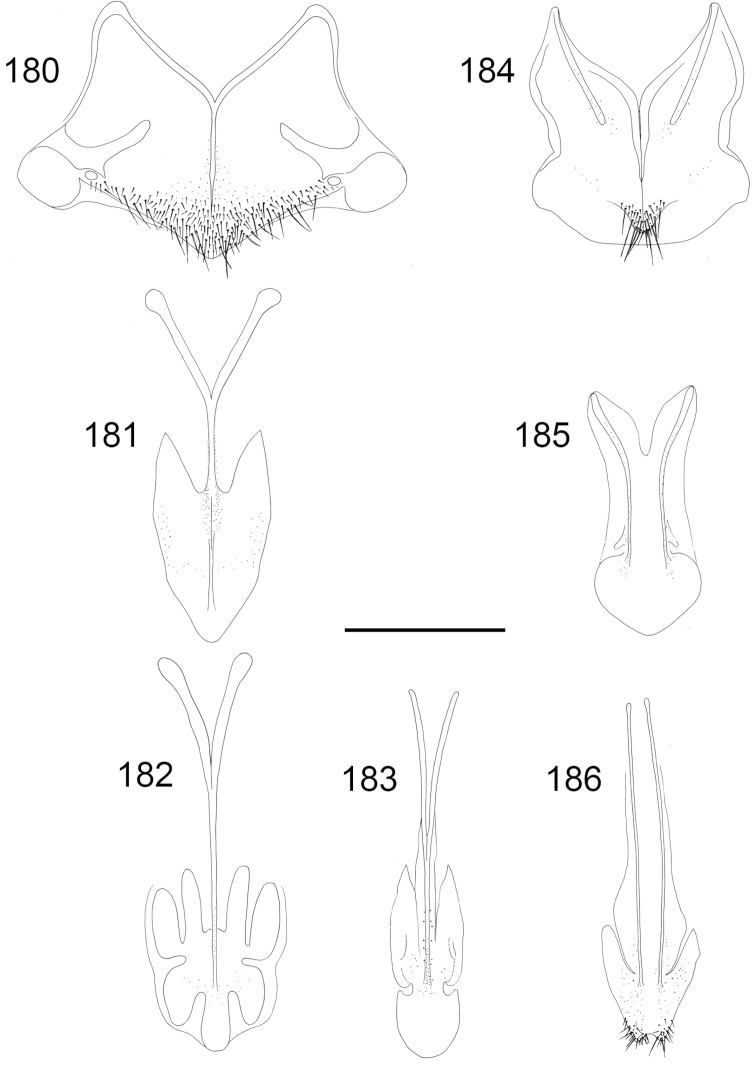
Internal tergites and sternites of *Chrysis borealis* sp. n. ♀: **180** T4 **181** T5 **182** T6 **183** T7 **184** S4 **185** S5 **186** S6. Illustrations based on a paratype from Sweden (MZH_GP.92691). Scale 1 mm. (Drawings: Juho Paukkunen)

Metasoma. Length 3.8–4.9 mm, maximum width 2.4–2.9 mm. Colour of tergites golden red or reddish, T1 anteriorly often greenish (Fig. 106). T1 with strong punctation, a weakly elevated medial line with sparser punctation, and very small and scattered punctures on interstices. Punctation of T2 anteriorly regular and dense, of the same strength as on T1 (Fig. 106). Punctation becoming weaker and more scattered laterally and posteriorly. T2 with prominent elevated medial line, anteriorly narrow and posteriorly broad, flat and shiny. T3 weakly saddle-shaped, with regular, strong and dense punctation, and often with elevated midline. Punctures of T3 on average as large as posteriorly on T2. Interstices usually with prominent microsculpture, whereby surface appears dull (Fig. 106). Posterior margin of T3 with four broadly separated teeth, intervals shallow. Medial interval usually about 1.5 times wider than lateral intervals. Subapical pit row with 15–20 black or bluish pits. Subapical lateral swellings relatively strong. Pubescence silvery white. Sternite colour green or blue, occasionally with golden reflections (Fig. 124). Black spots of S2 relatively large and subrectangular, their margins often vaguely delimited (Fig. 124). Ovipositor thin, similar to *Chrysis ignita* and *Chrysis impressa* (Fig. 92). Internal sternites and tergites similar to *Chrysis impressa* (Figs 180–186). T5 relatively narrow, about three times as long as broad, and tapering posteriorly, without lateral stigmae (Fig. 181).

####### Description of male.

Body length 6.7–9.0 mm, forewing length 4.7–6.0 mm (n = 15).

Head. Height 1.5–2.0 mm, width 1.9–2.4 mm, length 0.8–1.1 mm, shortest interocular distance 0.3–0.4 mm. Structure and colouration as in female, but scapal basin often slightly paler, shape of transverse frontal carina more variable, pubescence longer and mandible thicker. Sides of mandible basally slightly concave, gradually converging towards apex in lateral view. Relative lengths of P/F1/F2/F3 are 1/1.8/1.3/1.2 (Fig. 178). F1 usually 1.3–1.5 times as long as F2 (Table 1). F4–F10 as in female, or slightly shorter.

Mesosoma. Length 2.5–3.5 mm, width anterior to tegulae 1.7–2.4 mm. Length of pronotum medially 0.3–0.6 mm and width at anterior margin 1.5–2.1 mm. Structure as in female, but colouration usually somewhat lighter and pubescence longer. Margins of pronotum more often with golden reflections, and mesoscutum sometimes entirely blue. Mesoscutellum often medially violet, not always black, whereas mesoscutellum laterally, metanotum and propodeum violet, blue or golden green. Legs green, golden green or bluish with dark brown tibiae.

**Figures 187–195.**
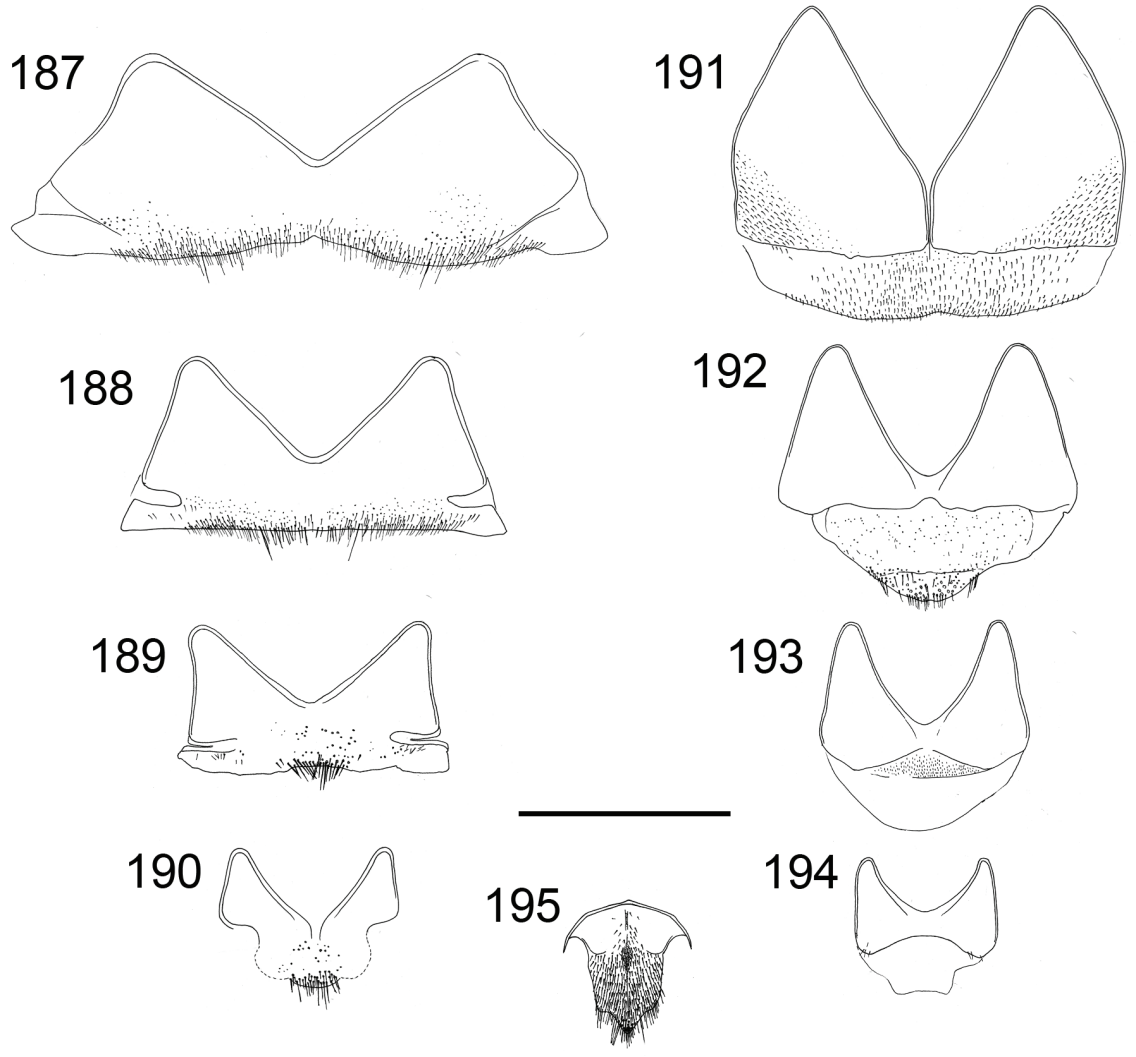
Internal tergites and sternites of *Chrysis borealis* sp. n. ♂: **187** T4 **188** T5 **189** T6 **190** T7 **191** S4 **192** S5 **193** S6 **194** S7 **195** S8. Illustrations based on a paratype from Finland (MZH_GP.92704). Scale 1 mm. (Drawings: Juho Paukkunen)

Metasoma. Length 3.3–4.4 mm, maximum width 2.1–2.8 mm. Colour of tergites as in female, but punctation of T1 and T2 usually denser and finer. T3 with very dense and homogenous punctation. Interstices shining without distinct microsculpture. T3 convex, not medially depressed as in female. Shape of apical teeth of T3 relatively variable. Medial interval narrower than or as wide as lateral intervals. Subapical pit row with 12–20 black pits. Subapical lateral swellings weak or nearly missing. Sternites with green, golden and reddish colour (Fig. 133), sometimes almost completely green. Black spots of S2 large and rounded (Fig. 133). Inner margin of paramere rounded (Fig. 136). Internal sternites and tergites similar to *Chrysis impressa* (Figs 187–195). S8 about 1.2 times as long as broad, posteriorly pointed and anteriorly rounded (Fig. 195).

####### Geographic variation.

Southern specimens from Estonia, Öland and Gotland are more uniform in colour than specimens from Finland, Norway and the Swedish mainland. The mesosoma of southern specimens is uniformly bright blue or violet with some greenish reflections, whereas in northern specimens, the mesoscutum and central part of the pronotum are commonly black or dark violet, and the margins of the pronotum and mesoscutellum are, in contrast, greenish or even golden green, especially in the males.

####### DNA analysis.

**Figure 196.**
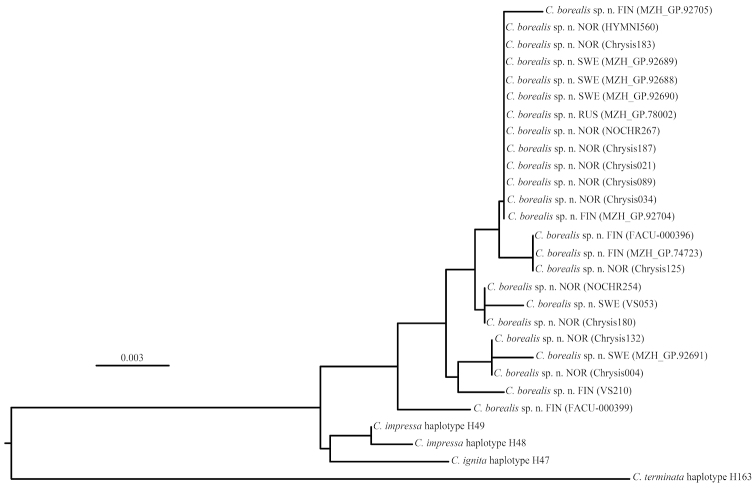
Kimura-2-parameter neighbour joining tree of all *Chrysis borealis* sp. n. samples and haplotypes of *Chrysis ignita* and *Chrysis impressa* (sensu Soon et al. 2014). The *Chrysis terminata* haplotype H163 (sensu Soon et al. 2014) is included as an outgroup.

Variable positions of the DNA barcode sequences of *Chrysis borealis* sp. n. and its sibling species, *Chrysis ignita* and *Chrysis impressa*, are presented in Table 2. Despite relatively high intraspecific variability, there are two diagnostic nucleotide mutations conserved in all sequences of *Chrysis borealis* sp. n. compared to *Chrysis ignita* and *Chrysis impressa*: G instead of A in position 241 and T instead of C in position 340. Additionally, there are three transitions shared with *Chrysis impressa*, but differing from *Chrysis ignita*, and one transition shared with *Chrysis ignita*, but differing from both haplotypes of *Chrysis impressa*. All samples of *Chrysis borealis* sp. n. cluster together indicating their closer relationship with each-other than the other two species (Fig. 196).

####### Distribution.

Denmark, Estonia, Finland, Norway, Sweden. Rare. – West Palearctic (?), general distribution poorly known. So far only known from the Nordic and Baltic countries and north-western Russia (Leningrad Oblast, Republic of Karelia, Murmansk Oblast) (Paukkunen et al. 2014).

####### Biology.

Habitat: rocky outcrops, cliffs, alpine meadows, forest margins. Often found on islands of the Baltic Sea and in Lapland, where other species of the *Chrysis ignita* group are uncommon. Adults have been found sitting on sun-exposed leaves of *Tussilago* and flowers of Apiaceae. They have also been collected using yellow pan traps. Flight period: late May to late August. A few specimens have been collected in early May and September. Host: *Ancistrocerus parietum* (Linnaeus) (Vespidae), based on records of *Chrysis borealis* sp. n. from islands and other coastal localities, where *Ancistrocerus parietum* is the only species of Eumeninae. In northern alpine areas, where *Ancistrocerus parietum* is not present, *Ancistrocerus scoticus* (Curtis) is the most likely host species.

####### Etymology.

The species epithet *borealis* is a Latin word derived from the Greek *boreas* which means north. We use it here as an adjective in the feminine case. The interpretation of borealis should be “northern”.

####### Remarks.

*Chrysis borealis* sp. n. is very closely related to *Chrysis impressa*, and cannot always be determined with certainty by morphological characters only. It is also easily confused with *Chrysis schencki*. The colouration of *Chrysis impressa* can sometimes be relatively dark and similar to *Chrysis borealis* n. sp., which possibly could be caused by cool weather during the larval and pupal development. Generally, the colouration of chrysidids becomes darker in northern and alpine localities with cool climatic conditions. *Chrysis borealis* n. sp. is mainly found in cooler habitats than *Chrysis impressa*, so it could be claimed to constitute only a dark ecological form of *Chrysis impressa* and not a distinct species. The slight, but constant divergence in the DNA barcode sequence (Table 2) and the statistically significant difference in the F1/F2 ratio (Table 1), however, supports the treatment of *Chrysis impressa* and *Chrysis borealis* n. sp. as distinct, but evolutionarily young sibling species. It is also noteworthy that the DNA barcode sequence of *Chrysis ignita* is very similar to *Chrysis borealis* n. sp. and *Chrysis impressa* (Table 2), but due to significant morphological and ecological differences *Chrysis ignita* undoubtedly forms a distinct species. Previous studies indicate that morphological and molecular differences between biologically well-defined species can be extremely small or even nonexistent in the *Chrysis ignita* group (Soon et al. 2014). An example is provided by *Chrysis mediata* and *Chrysis solida*, which are not always reliably separable by morphological characters or DNA barcodes, but represent ecologically well separated species with different hosts and habitats (Soon et al. 2014).

Several authors have earlier identified specimens of *Chrysis borealis* sp. n. erroneously as *Chrysis mediadentata* Linsenmaier, 1951 (Paukkunen et al. 2014). For example, Erlandsson (1971) reported *Chrysis mediadentata* from Sweden, Norway and eastern Fennoscandia based on such misidentified specimens. The dark colouration of *Chrysis mediadentata* can indeed resemble that of *Chrysis borealis* sp. n., but the species is easily distinguished by the breadth of the ovipositor, which in *Chrysis borealis* sp. n. is of the thin *ignita*-type (as in Fig. 92), and in *Chrysis mediadentata* of the thick *solida*-type (as in Fig. 91) (Linsenmaier 1987). Additionally, the apical teeth of *Chrysis mediadentata* have deeper intervals, and the two central teeth are more narrowly separated. The mandible of *Chrysis mediadentata* is thicker and the inner margin of the paramere of the male genitalia is angulate (as in Fig. 137). So far *Chrysis mediadentata* has not been found in the Nordic and Baltic countries. The northernmost confirmed records are from northern Germany (Jacobs and Kornmilch 2007).

**Table 1.** Length ratio of the first and second flagellomere in *Chrysis borealis* sp. n. and closely related species. All specimens of *Chrysis borealis* sp. n. and *Chrysis impressa*, and most of *Chrysis schencki* were identified by DNA barcoding. In *Chrysis ignita*, most specimens were identified by morphological characters only. A T-test was used for studying statistical differences between species.

**Species**	**Sex**	**N**	**F1/F2**				**P-value of T-test**		
			**Mean**	**Sd**	**Max**	**Min**	***Chrysis borealis* sp. n.**	***Chrysis ignita***	***Chrysis impressa***
*Chrysis borealis* sp. n.	male	16	1.38	0.07	1.52	1.25	-	-	-
*Chrysis ignita*	male	10	1.17	0.05	1.25	1.12	<0.0001	-	-
*Chrysis impressa*	male	18	1.27	0.07	1.41	1.14	<0.0001	0.0001	-
*Chrysis schencki*	male	10	1.26	0.09	1.39	1.11	0.0008	0.0078	0.3314
*Chrysis borealis* sp. n.	female	9	1.58	0.11	1.73	1.42	-	-	-
*Chrysis ignita*	female	10	1.41	0.07	1.50	1.29	0.0005	-	-
*Chrysis impressa*	female	13	1.44	0.11	1.63	1.25	0.0045	0.1582	-
*Chrysis schencki*	female	10	1.48	0.07	1.58	1.38	0.0128	0.0165	0.1916

**Table 2.** Variable positions in DNA barcode sequences of *Chrysis borealis* sp. n. (type specimens) compared with all known haplotypes of *Chrysis ignita* and *Chrysis impressa* (sensu Soon et al. 2014). Dots (.) indicate similarity with the reference sequence on the first line, hyphens (-) indicate missing data. Numbering corresponds to the COI barcode standard used for insects.

					1	1	2	2	2	3	3	3	3	3	4	6	6	6
	4	5	5	7	3	8	1	1	4	3	4	4	6	6	6	2	3	4
	3	0	5	7	3	4	2	4	1	7	0	3	1	4	9	8	4	6
*Chrysis borealis* sp. n. NOR (Chrysis183)	G	T	G	G	A	G	C	A	G	T	T	A	C	T	T	A	T	T
*Chrysis borealis* sp. n. NOR (HYMNI560)	.	.	.	.	.	.	.	.	.	.	.	.	.	.	.	-	-	-
*Chrysis borealis* sp. n. NOR (NOCHR267)	.	.	.	.	.	.	.	.	.	.	.	.	.	.	.	.	.	-
*Chrysis borealis* sp. n. NOR (Chrysis034)	.	.	.	.	.	.	.	.	.	.	.	.	.	.	.	.	.	-
*Chrysis borealis* sp. n. NOR (Chrysis021)	.	.	.	.	.	.	.	.	.	.	.	.	.	.	.	.	.	.
*Chrysis borealis* sp. n. NOR (Chrysis089)	.	.	.	.	.	.	.	.	.	.	.	.	.	.	.	.	.	.
*Chrysis borealis* sp. n. NOR (Chrysis187)	.	.	.	.	.	.	.	.	.	.	.	.	.	.	.	.	.	.
*Chrysis borealis* sp. n. SWE (MZH_GP.92690)	.	.	.	.	.	.	.	.	.	.	.	.	.	.	.	.	.	.
*Chrysis borealis* sp. n. SWE (MZH_GP.92688)	.	.	.	.	.	.	.	.	.	.	.	.	.	.	.	.	.	.
*Chrysis borealis* sp. n. SWE (MZH_GP.92689)	.	.	.	.	.	.	.	.	.	.	.	.	.	.	.	.	.	.
*Chrysis borealis* sp. n. FIN (MZH_GP.92704)	.	.	.	.	.	.	.	.	.	.	.	.	.	.	.	.	.	.
*Chrysis borealis* sp. n. RUS (MZH_GP.78002)	.	.	.	.	.	.	.	.	.	.	.	.	.	.	.	.	.	.
*Chrysis borealis* sp. n. FIN (MZH_GP.92705)	.	.	.	.	.	.	.	.	.	.	.	.	.	C	.	.	.	C
*Chrysis borealis* sp. n. NOR (Chrysis125)	.	.	.	.	.	.	.	T	.	.	.	.	.	.	.	.	.	C
*Chrysis borealis* sp. n. FIN (MZH_GP.74723)	.	.	.	.	.	.	.	T	.	.	.	.	.	.	.	.	.	C
*Chrysis borealis* sp. n. FIN (FACU-000396)	.	.	.	.	.	.	.	T	.	.	.	.	.	.	.	.	.	C
*Chrysis borealis* sp. n. SWE (TUZ616002)	.	C	.	.	.	.	.	.	.	.	.	.	T	.	.	.	.	.
*Chrysis borealis* sp. n. NOR (NOCHR254)	.	.	.	.	.	.	.	.	.	.	.	.	T	.	.	.	.	-
*Chrysis borealis* sp. n. NOR (Chrysis180)	.	.	.	.	.	.	.	.	.	.	.	.	T	.	.	.	.	.
*Chrysis borealis* sp. n. NOR (Chrysis132)	.	.	A	.	.	.	.	T	.	.	.	.	T	.	.	.	.	.
*Chrysis borealis* sp. n. NOR (Chrysis004)	.	.	A	.	.	.	.	T	.	.	.	.	T	.	.	.	.	.
*Chrysis borealis* sp. n. SWE (MZH_GP.92691)	.	.	A	.	.	.	.	T	.	C	.	.	T	.	.	.	.	.
*Chrysis borealis* sp. n. FIN (TUZ616001)	.	.	A	.	.	.	.	.	.	.	.	.	T	.	C	.	.	.
*Chrysis borealis* sp. n. FIN (FACU-000399)	.	.	A	.	.	.	T	.	.	.	.	T	.	.	.	.	C	.
*Chrysis ignita* haplotype H47	A	.	A	.	.	A	T	.	A	.	C	.	T	.	.	G	.	.
*Chrysis impressa* haplotype H48	.	.	A	A	G	.	T	.	A	.	C	.	T	.	.	.	.	.
*Chrysis impressa* haplotype H49	.	.	A	.	G	.	T	.	A	.	C	.	T	.	.	.	.	.

###### Chrysis terminata

Taxon classificationAnimaliaHymenopteraChrysididae

Dahlbom, 1854

Figs 153 , 154

Chrysis terminata Dahlbom, 1854: 261.
Chrysis ignita form A sensu Linsenmaier 1959: 156.

####### Diagnosis.

Length 6–10 mm. The species resembles closely *Chrysis ignita* by its colour, structure and habitus. However, the frontal carina has medially four tooth-like tubercles in both sexes (Figs 153, 154), the mesosoma is more homogeneously blue without extensive green areas on the pronotum and the mesoscutellum, and the body is slightly more elongate with more parallel sides.

####### Distribution.

Latvia, Lithuania, Norway, Sweden. Relatively common (especially in south-eastern Sweden, Öland and Gotland). – West Palearctic: from western Europe to central Asia (Linsenmaier 1997).

####### Biology.

Habitat: forest margins, clearings and gardens with sun-exposed dead wood, but also sparsely vegetated sandy areas. Flight period: late April to early August. The flight begins earlier than in other species of the *Chrysis ignita* group. Host: *Ancistrocerus nigricornis* (Curtis) (Vespidae) (van Lith 1954, Linsenmaier 1959, our own obs.).

##### *Chrysis smaragdula* group

###### Chrysis sexdentata

Taxon classificationAnimaliaHymenopteraChrysididae

Christ, 1791

Fig. 81

Chrysis sexdentata Christ, 1791: 404.

####### Diagnosis.

Length 7–11 mm. The species is easily recognised due to its unique combination of a red metasoma and six apical teeth. The head and the mesosoma are greenish, dark blue or nearly black with coppery reflections, whereas the metasoma is dorsally purple-red or coppery red. The tergites are coarsely punctured, and the posterior margin of T3 has six sharp teeth (Fig. 81). The shape of the body is robust and compact.

####### Distribution.

Latvia. Very rare. Only one male specimen is known from central Latvia (Ropaži, 1.VI.1961, leg. V. Tumšs) (Tumšs and Maršakovs 1970). – Trans-Palearctic: from Europe and northern Africa to western and central Asia (Linsenmaier 1959, 1999).

####### Biology.

Habitat: gardens with dead wood, old brick walls, old fences and/or stones (Trautmann 1927, 1930). Adults occasionally visit flowers of Apiaceae and Euphorbiaceae (Linsenmaier 1997, Rosa 2004). Flight period: June to July. Host: *Euodynerus dantici* (Rossi) (Martynova and Fateryga 2015) and possibly also *Ancistrocerus parietum* (Linnaeus) (Vespidae) (Mocsáry 1912, Berland and Bernard 1938). Host records implicating solitary bees (e.g. *Osmia brevicornis* (Fabricius), *Osmia caerulescens* (Linnaeus), *Hoplitis adunca* (Panzer) and *Megachile sicula* (Rossi)) (Trautmann 1927) are dubious, as pointed out by Kunz (1994).

###### Chrysis equestris

Taxon classificationAnimaliaHymenopteraChrysididae

Dahlbom, 1854

Figs 80 , 89 , 112 , 134 , 151

Chrysis sexdentata ? Dahlbom, 1831: 30, not Christ, 1791.
Chrysis zetterstedti of authors, not Dahlbom, 1845.
Chrysis equestris Dahlbom, 1854: 307.
Chrysis fasciata of authors, not Olivier, 1790.

####### Diagnosis.

Length 7–10 mm. Both sexes have a mostly dark blue or black, partially violet, body with green reflections on the frons, margins of the pronotum, mesoscutum, mesoscutellum and mesopleuron. The tergites have contrasting golden red or golden green bands posteriorly (except on the apical rim), which are especially wide laterally on T1 and T2. The colour and form of the bands is quite variable, usually they are wider and more reddish in the female than in the male. The species closely resembles *Chrysis zetterstedti*, but is characterised by the following differences: the black spots of S2 are narrower, usually not extending to the lateral margins of the sternite (Fig. 112), T5 of the female (on ovipositor) is broader and has a longitudinal medial groove (Fig. 89), the head is broader, especially in female (shortest distance between the compound eyes is slightly longer than the diameter of an eye) (Fig. 151), the gonostyle is shorter, the cuspis is apically curved (not straight) (Fig. 134), and the propodeal tooth is slightly convex or straight ventrally (not lobate).

####### Distribution.

Estonia, Finland, Lithuania, Norway, Sweden. Rare. – Trans-Palearctic: from western Europe to Russian Far East (Sakhalin).

####### Biology.

Habitat: forest margins, clearings and gardens with sun-exposed dead wood. Adults are usually found on sun-exposed dead tree trunks and stumps, most often of *Populus*, but also of *Salix*, *Betula* and *Alnus*, rarely *Picea* and *Pinus*. They also fly near log piles, telephone poles and walls of old wooden buildings (Frey 1915, Linsenmaier 1997). Flight period: mid-May to early August. Host: *Discoelius dufourii* Lepeletier and *Discoelius zonalis* (Panzer) (Vespidae) (Pärn et al. 2014, our own obs.).

###### Chrysis zetterstedti

Taxon classificationAnimaliaHymenopteraChrysididae

Dahlbom, 1845

Figs 90 , 113 , 135 , 152

Chrysis sexdentata ? Dahlbom, 1831: 30, not Christ, 1791.
Chrysis Zetterstedti Dahlbom, 1845: 11.
Chrysis fasciata of authors, not Olivier, 1790.

####### Diagnosis.

Length 6–9 mm. The species resembles *Chrysis equestris*, but differs from it by the following characters: the black spots of S2 are broader, extending to the lateral and anterior margins of the sternite (Fig. 113), T5 of the female is narrower and does not have a longitudinal medial groove (Fig. 90), the head is narrower (the shortest distance between the compound eyes is shorter or as long as the diameter of an eye) (Fig. 152), the gonostyle is more elongated, as long as the cuspis, the cuspis is apically straight (not curved) (Fig. 135), and the propodeal tooth is weakly lobate ventrally (not convex or straight).

####### Distribution.

Estonia, Latvia, Lithuania, Sweden. Rare. – Trans-Palearctic: from North Europe to Siberia. Records from the East Palearctic Region refer to *Chrysis fasciata daphne* Smith, 1874 (Rosa et al. 2014).

####### Biology.

Habitat: forest margins and clearings with sun-exposed dead tree trunks and stumps (e.g. *Quercus*). Flight period: probably similar to that of *Chrysis equestris*, most specimens have been collected in July. Host: unknown, possibly *Euodynerus notatus* (Jurine) (N. Johansson pers. obs.).

####### Remarks.

Several authors have considered *Chrysis zetterstedti* to be either a synonym (e.g. Trautmann 1927, Kimsey and Bohart 1991) or a subspecies (Linsenmaier 1959, 1997, Rosa and Soon 2012) of *Chrysis fasciata*. However, molecular and morphological studies have shown that *Chrysis zetterstedti* most likely represents a valid species (Paukkunen et al. 2014). The occurrence of *Chrysis zetterstedti* in central and southern Europe is still uncertain.

###### Genus Trichrysis

Taxon classificationAnimaliaHymenopteraChrysididae

Lichtenstein, 1876

Figs 197 , 198–199

Trichrysis Lichtenstein, 1876: 27.

####### Note.

**Figure 197.**
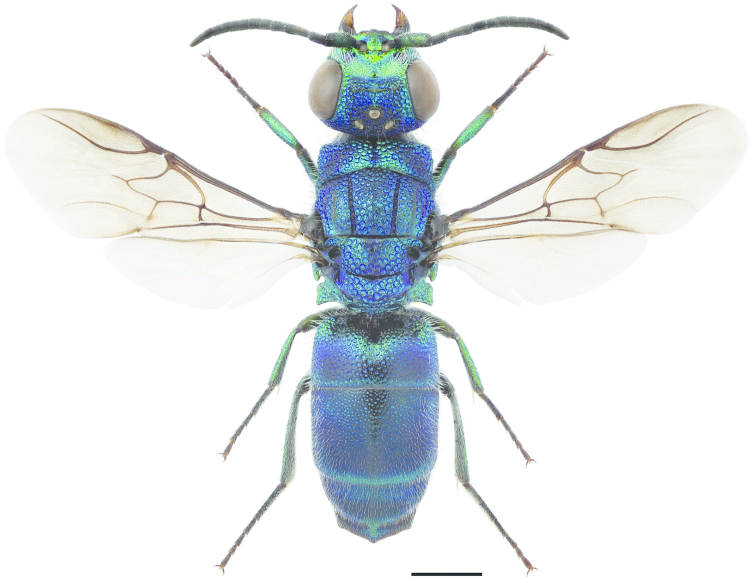
*Trichrysis cyanea* ♀. Scale 1 mm.

**Figures 198–199.**
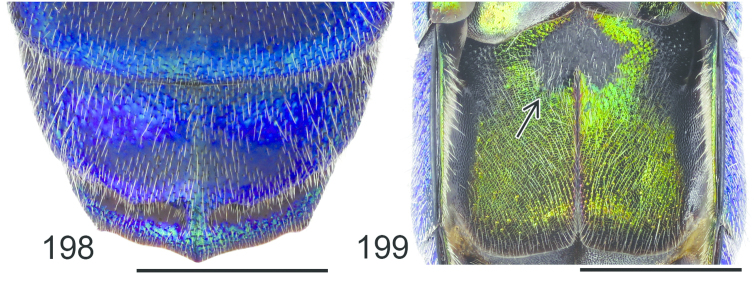
*Trichrysis cyanea* ♀: **198** T3, dorsal view **199** S2, ventral view. Scale 1 mm.

In Europe, this genus is characterised by the tridentate posterior margin of T3 (Figs 197, 198), the medially located small black spots of S2 (Fig. 199), and the simple metanotum (Kimsey and Bohart 1991). The European species are relatively small (body length ca 4–8 mm) and completely blue or green in colour. Small males are often blackish, at least dorsally. The hosts are cavity-nesting crabronid and pompilid wasps, and possibly also solitary vespid wasps and megachilid bees (Trautmann 1927, Pärn et al. 2014). The genus includes 27 species in the Palearctic, Afrotropical and Oriental Regions (Kimsey and Bohart 1991, Strumia 2009). Three species have been found in Europe (Rosa and Soon 2012), and one, *Trichrysis cyanea*, from the Nordic and Baltic countries (Paukkunen et al. 2014).

###### Trichrysis cyanea

Taxon classificationAnimaliaHymenopteraChrysididae

(Linnaeus, 1758)

Figs 197 , 198–199

Sphex cyanea Linnaeus, 1758: 572.
Trichrysis cyanea : Trautmann and Trautmann 1919: 34.

####### Diagnosis.

Length 4–8 mm. The species is characterised by its completely green, blue or violet body (Fig. 197) and the tridentate posterior margin of T3 (Fig. 198). The male in particular is often dorsally or rarely almost completely black. The lateral teeth of T3 are often more like angles, and sometimes all teeth can be small or rounded and inconspicuous. The black spots of S2 are small and located close together (Fig. 199).

####### Distribution.

Denmark, Estonia, Finland, Latvia, Lithuania, Norway, Sweden. Very common. – Trans-Palearctic: from Europe and northern Africa to central Asia, Siberia, Korea, China and Japan (Linsenmaier 1999, Kurzenko and Lelej 2007, Rosa et al. 2014).

####### Biology.

Habitat: forest margins, clearings and gardens with sun-exposed dead wood (e.g. dead tree trunks, log and branch piles, walls of wooden buildings or poles). Adults occasionally visit flowers of Apiaceae and Rosaceae (Kusdas 1956, Rosa 2004, our own obs.). Flight period: late May to early September. Host: primarily species of *Trypoxylon* Latreille (Crabronidae), but also *Auplopus carbonarius* (Scopoli) and species of *Dipogon* Fox (Pompilidae), and possibly other cavity-nesting crabronid wasps (Dufour and Perris 1840, Morgan 1984, Brechtel 1986, Asís et al. 1994, Gathmann and Tscharntke 1999, Tormos et al. 1996, Pärn et al. 2014, our own obs.). Also many cavity-nesting solitary bee species have been reported as hosts, but these records are rather unreliable due to the very different biology of bees compared to crabronid and pompilid hosts.

###### Genus Chrysura

Taxon classificationAnimaliaHymenopteraChrysididae

Dahlbom, 1845

Figs 200 , 201–208

Chrysura Dahlbom, 1845: 6.
Holochrysis Rye, 1878: 134.

####### Note.

Diagnostic characters of this genus include the nearly flat and densely punctate frons, the lack of a transverse frontal carina (Figs 201, 202), and the long malar space. Usually the mandible is also toothed subapically (Fig. 202), the proximal flagellomeres of the male are swollen ventrally (Fig. 204) and the pronotum is shorter than the mesoscutellum. The radial cell of the forewing is closed and the posterior margin of T3 is rounded without apical teeth (Figs 206–208). *Chrysura* is the second largest genus in the tribe Chrysidini. It includes 117 valid species, of which 106 are distributed in the Palearctic Region (Kimsey and Bohart 1991, Rosa and Lotfalizadeh 2013). The hosts consist of solitary bees of the family Megachilidae. The European fauna includes 50 species and several subspecies (Rosa and Soon 2012). Five species have been recorded in the Nordic and Baltic countries (Paukkunen et al. 2014). Species-groups below follow Kimsey and Bohart (1991).

**Figure 200.**
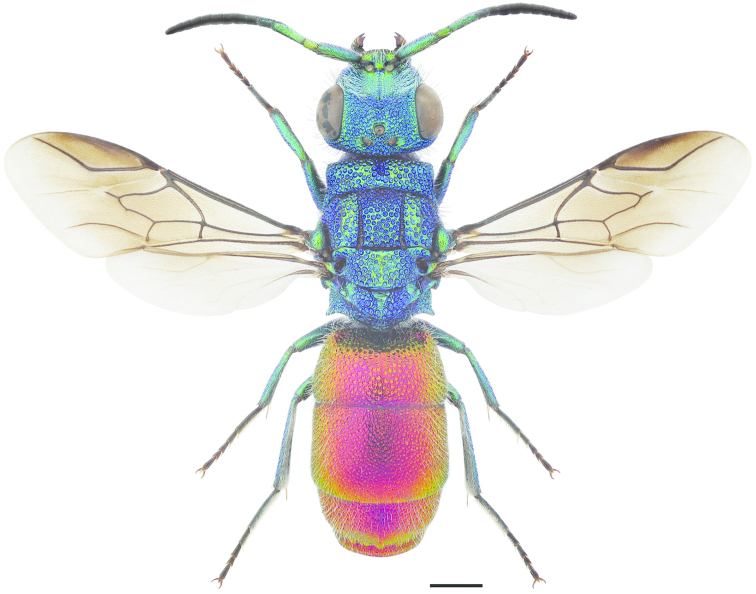
*Chrysura radians* ♀. Scale 1 mm.

**Figures 201–208.**
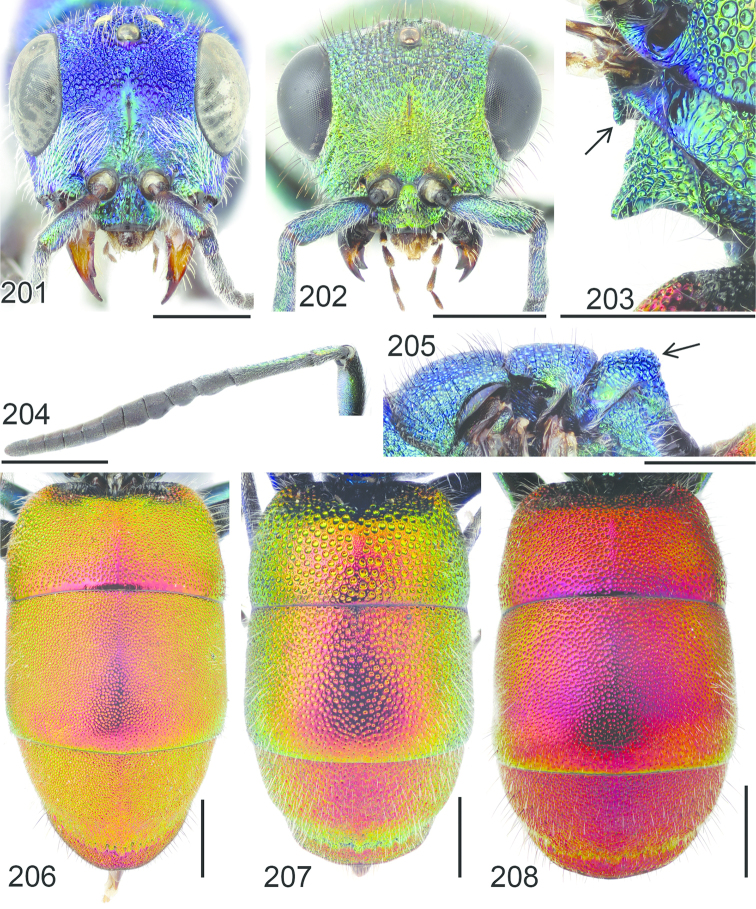
Head, frontal view: **201** *Chrysura austriaca* ♀ **202** *Chrysura hirsuta* ♀. Metanotal tooth, dorsal view: **203** *Chrysura hirsuta* ♀. Antenna: **204** *Chrysura radians* ♂. Mesoscutum, mesoscutellum, metanotum and propodeum, lateral view (arrow indicating metascutellum): **205** *Chrysura trimaculata* ♀. Metasoma, dorsal view: **206** *Chrysura trimaculata* ♀, **207** *Chrysura radians* ♀ **208** *Chrysura hirsuta* ♀. Scale 1 mm.

####### Key to *Chrysura* species of the Nordic and Baltic countries

**Table:** 

1	Mesosoma dorsally bright red	***Chrysura dichroa* (Dahlbom)**
–	Mesosoma entirely blue-green with golden (or rarely coppery) reflections, without red colour	**2**
2	Head broad ventrally, with nearly parallel malar spaces, width between mandible bases about 1.5 times as long as compound eye (Fig. 201). Mandibles without or with very small subapical tooth. Male with simple antennal segments. Metascutellum evenly rounded, not elevated medially	***Chrysis austriaca* (Fabricius)**
–	Head narrow ventrally, with convergent malar spaces, width between the mandible bases equivalent or slightly longer than length of compound eye (Figs 202). Mandibles with large subapical tooth (Fig. 202). Male with ventrally swollen antennal segments (Fig. 204). Metascutellum medially elevated or relatively rounded	**3**
3	T3 of female long and ovoid, posterior pit row very weakly developed (Fig. 206). Metascutellum sharply elevated medially (Fig. 205). Punctation of tergites very dense and homogeneous (Fig. 206). Setae on posterior margin of T3 brown	***Chrysura trimaculata* (Förster)**
–	T3 of female short and wide, with distinct pit row posteriorly (Figs 207, 208). Metascutellum relatively rounded, not sharply elevated medially. Punctation of tergites homogeneous or heterogeneous (Figs 207, 208). Setae on posterior margin T3 brown or white	**4**
4	Punctation of tergites heterogeneous, consisting of large punctures and interspersed small punctures (Fig. 207). Setae on T3 white. Metanotal tooth indistinct. Third antennal segment about 3.5 times as long as broad	***Chrysura radians* (Harris)**
–	Punctation of tergites homogeneous, consisting of only small punctures (Fig. 208). Setae on T3 brown. Metanotal tooth distinct (Fig. 203). Third antennal segment about 3 times as long as broad	***Chrysura hirsuta* (Gerstaecker)**

##### *Chrysura austriaca* group

###### Chrysura austriaca

Taxon classificationAnimaliaHymenopteraChrysididae

(Fabricius, 1804)

Fig. 201

Chrysis austriaca Fabricius, 1804: 173.
Chrysura austriaca : Dahlbom 1845: 6.

####### Diagnosis.

Length 8–12 mm. The species differs from other similarly coloured species of *Chrysura* by its ventrally broader head (Fig. 201) and simple mandible, which lacks or has only a very small subapical tooth (Fig. 201). The antennal segments of the male are not ventrally swollen. The head and mesosoma are blue, often with golden green reflections laterally on the mesoscutum, and the metasoma is golden red.

####### Distribution.

Lithuania. Very rare. The species is known from three localities in Lithuania (Puvočiai, Trakai, Vilnius) (Orlovskytė et al. 2010). – Trans-Palearctic: from Europe and northern Africa to Siberia and Japan (Linsenmaier 1997, Kurzenko and Lelej 2007).

####### Biology.

Habitat: forest margins and gardens. Often found from wooden poles, walls, fence posts, loess, clay or old brick walls or rocks (Trautmann 1927, Kunz 1994, Linsenmaier 1997). Adults occasionally visit flowers of Apiaceae and Euphorbiaceae (Linsenmaier 1997, Rosa 2004). Flight period: June to early August. In Lithuania, specimens have been collected in July and early August. Host: *Hoplitis adunca* (Panzer) (Megachilidae) (e.g. Frey-Gessner 1887, Trautmann and Trautmann 1919, Linsenmaier 1959), possibly also *Hoplitis anthocopoides* (Schenck) and *Osmia parietina* Curtis (Schenck 1856, Mocsáry 1879, Banaszak 1980).

##### *Chrysura dichroa* group

###### Chrysura dichroa

Taxon classificationAnimaliaHymenopteraChrysididae

(Dahlbom, 1854)

Chrysura nitidula Dahlbom, 1845: 7, nomen oblitum, not *Chrysis nitidula* Fabricius, 1775.
Chrysis Dahlbom, 1854: 143.
Chrysis dichroa Dahlbom, 1854: 146.
Chrysura dichroa : Kimsey and Bohart 1991: 488.

####### Diagnosis.

Length 5–9 mm. The species is easy to differentiate from other North European *Chrysura* species by its bright red pronotum, mesoscutum and mesoscutellum. The head, mesopleuron, metanotum, propodeum and legs (excluding tarsi) are green or blue, and the metasoma is golden red, as in other species of the genus. The tergites are very densely and finely punctured.

####### Distribution.

Sweden. Very rare. Only one female specimen is known from Västergötland, southern Sweden, collected probably in the 1830s (leg. L. Gyllenhal). – West Palearctic: southern and central Europe, south-eastern Asia (Linsenmaier 1997).

####### Biology.

Habitat: rock mounds, scree formations, rocky outcrops, rock walls and dry meadows, usually in areas with calcareous bedrock (Kunz 1994, Linsenmaier 1997). Adults occasionally visit flowers of Apiaceae, Asteraceae and Rosaceae (Linsenmaier 1997, Rosa 2004, 2006). Flight period: late May to mid-August. Host: species of *Osmia* Panzer (Megachilidae) which build nests in empty snail shells, primarily *Osmia rufohirta* (du Buysson 1891, Ferton 1905, Malyshev 1968, Bonelli 1974), but also *Osmia aurulenta* (Panzer), *Osmia versicolor* Latreille, *Osmia andrenoides* Spinola, *Osmia spinulosa* (Kirby), *Osmia ferruginea* Latreille and *Osmia caerulescens* (Linnaeus) (Dalla Torre 1892, Ferton 1905, Grandi 1959, Heinrich 1964).

##### *Chrysura radians* group

###### Chrysura hirsuta

Taxon classificationAnimaliaHymenopteraChrysididae

(Gerstaecker, 1869)

Figs 202 , 203 , 208

Chrysis hirsuta Gerstaecker, 1869: 185.
Chrysis bicolor Dahlbom, 1829: 10, in part, not Lepeletier, 1806.
Chrysis Thomson, 1870: 106.
Chrysura hirsuta : Morgan 1984: 19.

####### Diagnosis.

Length 7–11 mm. The species resembles other similarly coloured species of *Chrysura*, but the metascutellum is flatter (not sharply elevated as in *Chrysura trimaculata*), the punctation of the tergites is homogeneous and dense (Fig. 208) (not heterogeneous as in *Chrysura radians*) and the mandible has a large subapical tooth (Fig. 202) (tooth lacking or small in *Chrysis austriaca*). The head and mesosoma are dark green or blue, often with golden green reflections, whereas the metasoma is golden red or rarely golden greenish.

####### Distribution.

Estonia, Finland, Lithuania, Norway, Sweden. Relatively rare. – Trans-Palearctic: from western Europe to China, Korea and Japan (Linsenmaier 1959, Rosa et al. 2014).

####### Biology.

Habitat: dry meadows, forest margins and clearings. Adults are often found flying near the ground, rocks or dead wood (Linsenmaier 1959, 1997, Rosa 2006). Flight period: April to July. A female specimen, collected at the end of September in SW Finland, might belong to a second generation. Host: *Osmia inermis* (Zetterstedt), *Osmia nigriventris* (Zetterstedt), *Osmia parietina* Curtis, *Osmia spinulosa* (Kirby), *Osmia uncinata* Gerstaecker, and *Hoplitis tuberculata* (Nylander) (Megachilidae) (Smith 1862, Trautmann 1918, Trautmann 1927, Morgan 1984, our own obs.). Host records mentioning bees of other genera, e.g. *Chelostoma florisomne* (Linnaeus) (Megachilidae) (Frey-Gessner 1887), are questionable as supporting evidence is lacking.

###### Chrysura radians

Taxon classificationAnimaliaHymenopteraChrysididae

(Harris, 1776)

Figs 200 , 204 , 207

Chrysis radians Harris, 1776: 69.
Chrysis bicolor Dahlbom, 1829: 10, in part, not Lepeletier, 1806.
Chrysis pustulosa Abeille de Perrin, 1878: 6.
Chrysura radians : Morgan 1984: 19.

####### Diagnosis.

Length 8–11 mm. The species differs from other similarly coloured species of the genus by its heterogeneous tergal punctation, which consists of large punctures and interspersed small punctures (Figs 200, 207). The metascutellum is slightly elevated (but not as sharply as in *Chrysura trimaculata*) and has a large triangular fovea antero-medially. The head and mesosoma are mainly green or blue, whereas the metasoma is dorsally golden red or violet-red (Fig. 200). The punctures on the pronotum, mesoscutum and mesoscutellum are often contrastingly blue compared to the greenish interstices.

####### Distribution.

Denmark, Estonia, Latvia, Lithuania, Norway, Sweden. Relatively rare. – Trans-Palearctic: from western Europe and northern Africa to western Asia and Siberia (Linsenmaier 1959).

####### Biology.

Habitat: forest margins, clearings and gardens with sun-exposed dead wood. Occasionally also found on brick walls, clay walls or rocky outcrops (Trautmann 1927). Adults visit flowers of Apiaceae and Euphorbiaceae (Rosa 2004) and also feed on honeydew of aphids (Linsenmaier 1997). Flight period: June to August. Host: solitary bees of *Osmia* Panzer and *Hoplitis* Klug (Megachilidae), which usually nest in cavities in dead wood. In North Europe, probably mainly *Hoplitis adunca* (Panzer), *Hoplitis anthocopoides* (Schenck), *Osmia caerulescens* (Linnaeus) and/or *Osmia leaiana* (Kirby) (Frey-Gessner 1887, du Buysson 1891, Trautmann 1927, Stöckhert 1933).

###### Chrysura trimaculata

Taxon classificationAnimaliaHymenopteraChrysididae

(Förster, 1853)

Figs 205 , 206

Chrysis trimaculata Förster, 1853: 307.
Chrysura trimaculata : Kimsey and Bohart 1991: 497.

####### Diagnosis.

Length 9–11 mm. Compared to other similarly coloured species of *Chrysura*, the metascutellum is more sharply elevated (Fig. 205) and T3 of the female is longer and more ovoid in shape (Fig. 206). Punctation of the tergites is very dense and homogeneous (Fig. 206). The black spots on S2 are very large, and the eyes are strongly bulging above genae. The head and mesosoma are dark green or green-blue and the metasoma is golden red.

####### Distribution.

Sweden. Rare. Only found on the islands of Öland and Gotland. – West Palearctic: southern and central Europe, Asia Minor (Linsenmaier 1997).

####### Biology.

Habitat: sparsely vegetated sandy areas. Adults occasionally visit flowers of Apiaceae, Asteraceae, Euphorbiaceae, Rosaceae and Salicaceae (Kusdas 1956, Ressl 1966). Flight period: from April to June. Host: species of *Osmia* Panzer (Megachilidae) which construct nests in empty shells of larger terrestrial gastropods. In Sweden, the main hosts are probably *Osmia bicolor* (Schranck) and *Osmia aurulenta* (Panzer), possibly also *Osmia spinulosa* (Kirby) (Trautmann 1927, Berland and Bernard 1938, Heinrich 1964, Sörensson 2008).

#### Tribe Parnopini

This tribe has been treated as a valid subfamily, Parnopinae, by several authors (Mocsáry 1889, Linsenmaier 1959, Mingo 1994, Rosa 2006). We follow the classification of Kimsey and Bohart (1991) and include it in the Chrysidinae. Parnopini is characterised by several morphological features, such as the strongly developed mouthparts, the large and broad tegula which covers the wing basally, the irregularly and finely dentate posterior margin of the last external tergite, and the number of exposed metasomal tergites, which is four in the male and three in the female. The tribe consists of three genera, one of which, *Parnopes*, is found in Europe.

##### Genus Parnopes

Taxon classificationAnimaliaHymenopteraChrysididae

Latreille, 1797

Fig. 209

Parnopes Latreille, 1797: 126.

###### Note.

The genus can be distinguished from other genera of Parnopini by the reduced palpi, the large metascutellar projection and the larger body size. Members of the genus are parasites of ground-nesting solitary wasps of the tribe Bembicini (Crabronidae: Bembicinae). A total of 16 species are recognised, most of which occur in the Palearctic and Nearctic Regions (with 4 and 7 species, respectively) (Kimsey and Bohart 1991). A few species are known from India and Africa. Only one species, *Parnopes grandior*, is found in Europe (Rosa and Soon 2012). The genus has been divided into species-groups by Kimsey and Bohart (1991).

##### Parnopes grandior

Taxon classificationAnimaliaHymenopteraChrysididae

(Pallas, 1771)

Fig. 209

Chrysis grandior Pallas, 1771: 474.
Parnopes grandior : Mocsáry 1882: 74.

###### Diagnosis.

**Figure 209.**
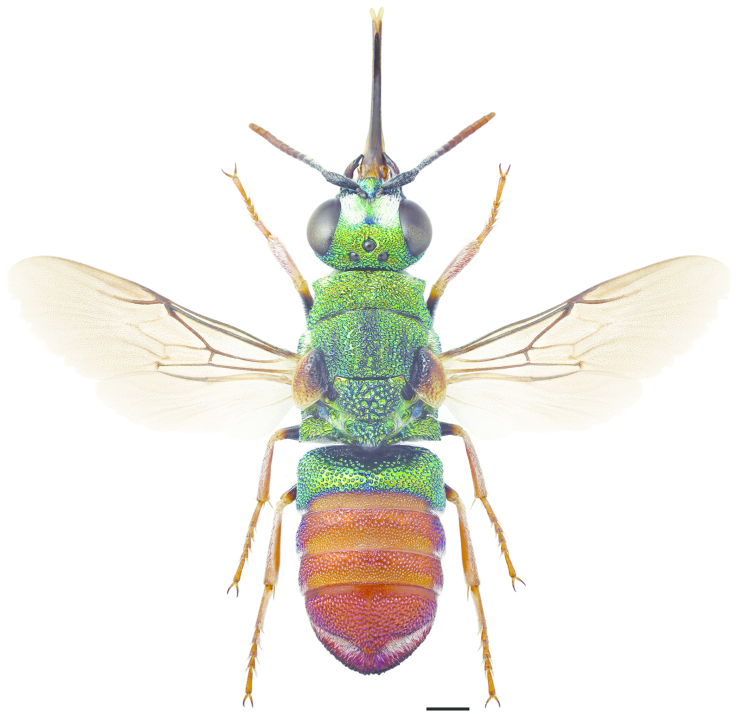
*Parnopes grandior* ♂. Scale 1 mm.

Length 8–12 mm. The species is easy to differentiate from other North European cuckoo wasps by its unique structure and colouration. The head, mesosoma and most of T1 are green or green-blue, with golden or coppery reflections, especially in the female. The metasoma behind T1, tegulae and tibiae are usually non-metallic red (Fig. 209). Sometimes also T2, T3 (and T4) have a metallic sheen laterally or even dorsally. The female has three and the male four external tergites, and the posterior margin of the last tergite is irregularly dentate (Fig. 209). The mouthparts are longer than the rest of the head, and the tegulae are large covering the wing bases (Fig. 209).

###### Distribution.

Lithuania. Very rare. The species has been recorded from five localities in southern Lithuania (Orlovskytė et al. 2010). – West Palearctic: from Europe and northern Africa to Yemen and southwestern Asia (Linsenmaier 1997, 1999).

###### Biology.

Habitat: xerothermic sparsely vegetated sandy areas. Adults often visit flowers of several different families (Bischoff 1923, Trautmann 1927, Molitor 1935, Schneid 1954, Linsenmaier 1997, Rosa 2004). Flight period: July to August. Host: *Bembix rostrata* (Linnaeus) (Crabronidae) (Gauss 1967), in southern Europe also other species of *Bembix* Fabricius (Linsenmaier 1968, Kunz 1994). The larva does not consume the host larva until it is fully grown (Malyshev 1968).

**Table 3.** Distribution of chrysidid species in the Nordic and Baltic countries. DK = Denmark, EE = Estonia, FI = Finland, LV = Latvia, LT = Lithuania, NO = Norway, SE = Sweden. 1 = recorded before 2000, 2 = recorded from 2000 onwards.

Species	DK	EE	FI	LV	LT	NO	SE
*Cleptes nitidulus* (Fabricius, 1793)	1, 2	2	1, 2	1			1, 2
*Cleptes semicyaneus* Tournier, 1879	1					2	2
*Cleptes semiauratus* (Linnaeus, 1761)	1, 2	1, 2	1, 2	1	1	1, 2	1, 2
*Omalus biaccinctus* (du Buysson, 1892)	1	1, 2	1, 2	1		2	1, 2
*Omalus aeneus* (Fabricius, 1787)	1, 2	1, 2	1, 2	1, 2	1, 2	1, 2	1, 2
*Omalus puncticollis* (Mocsáry, 1887)						2	2
*Pseudomalus pusillus* (Fabricius, 1804)	1			1, 2	1, 2		
*Pseudomalus auratus* (Linnaeus, 1758)	1, 2	1, 2	1, 2	1, 2	1, 2	1, 2	1, 2
*Pseudomalus triangulifer* (Abeille de Perrin, 1877)	1	1, 2	1, 2	1, 2	1, 2	1, 2	1, 2
*Pseudomalus violaceus* (Scopoli, 1763)	1, 2	1, 2	1, 2	1, 2	1	1, 2	1, 2
*Philoctetes truncatus* (Dahlbom, 1831)	1	1, 2		2			1
*Elampus constrictus* (Förster, 1853)	1	1	1, 2			1, 2	1, 2
*Elampus foveatus* (Mocsáry, 1914)		2	1, 2			2	1, 2
*Elampus panzeri* (Fabricius, 1804)	1, 2	1, 2	1, 2	1	1	1, 2	1, 2
*Holopyga fervida* (Fabricius, 1781)	1						
*Holopyga metallica* (Dahlbom, 1854)			1, 2				
*Holopyga generosa* (Förster, 1853)	1	2	1, 2	1	1, 2		1, 2
*Holopyga inflammata* (Förster, 1853)			1		1		
*Hedychrum gerstaeckeri* Chevrier, 1869	1	1, 2	1, 2	1, 2	1, 2		
*Hedychrum rutilans* Dahlbom, 1854	1, 2	1, 2	1, 2	1, 2	1, 2		
*Hedychrum nobile* (Scopoli, 1763)	1, 2	1, 2	1, 2	1, 2	1, 2	1, 2	1, 2
*Hedychrum niemelai* Linsenmaier, 1959	1, 2	1, 2	1, 2	1, 2	1, 2	1, 2	1, 2
*Hedychrum chalybaeum* Dahlbom, 1854				1	1		
*Hedychridium zelleri* (Dahlbom, 1845)			1, 2				
*Hedychridium ardens* (Coquebert, 1801)	1, 2	1, 2	1, 2	1, 2	1, 2	1, 2	1, 2
*Hedychridium coriaceum* (Dahlbom, 1854)	1	2	1, 2	1, 2	1, 2		1, 2
*Hedychridium cupreum* (Dahlbom, 1845)	1, 2	1, 2	1, 2	1, 2	1	1, 2	1, 2
*Hedychridium purpurascens* (Dahlbom, 1854)		2					
*Hedychridium caputaureum* Trautmann & Trautmann, 1919		2	1, 2	2	2		1, 2
*Hedychridium roseum* (Rossi, 1790)	1	1, 2	1, 2	1, 2	1, 2	1, 2	1, 2
*Pseudospinolia neglecta* (Shuckard, 1837)	1, 2	1, 2	1, 2	1, 2	1, 2	1	1, 2
*Spinolia unicolor* (Dahlbom, 1831)	1			1			1, 2
*Chrysis gracillima* Förster, 1853		2					
*Chrysis bicolor* Lepeletier, 1806	1, 2	1, 2	1, 2	1, 2	1		1, 2
*Chrysis westerlundi* Trautmann, 1927			1, 2				
*Chrysis illigeri* Wesmael, 1839	1, 2	1, 2	1, 2	1	1, 2	1, 2	1, 2
*Chrysis succincta* Linnaeus, 1767	1			1, 2	1		
*Chrysis leachii* Shuckard, 1837	1						
*Chrysis scutellaris* Fabricius, 1794	1				1		1, 2
*Chrysis splendidula* Rossi, 1790				1, 2			
*Chrysis rutilans* Olivier, 1791		1, 2	1, 2	1	1		1, 2
*Chrysis pulcherrima* Lepeletier, 1806	1						
*Chrysis viridula* Linnaeus, 1761	1, 2	1, 2	1, 2	1, 2	1, 2	1, 2	1, 2
*Chrysis graelsii* Guérin-Méneville, 1842		1, 2	1, 2	1, 2	1, 2		
*Chrysis indigotea* Dufour & Perris, 1840							1
*Chrysis fulgida* Linnaeus, 1761	1, 2	1, 2	1, 2	1, 2	1, 2	1, 2	1, 2
*Chrysis iris* Christ, 1791	1	1, 2	1, 2	1, 2	1, 2		1, 2
*Chrysis ruddii* Schuckard, 1837	1, 2	1, 2	1, 2	1, 2	1	1, 2	1, 2
*Chrysis corusca* Valkeila, 1971		1, 2	2	1	1, 2	2	1, 2
*Chrysis clarinicollis* Linsenmaier, 1951		2					
*Chrysis vanlithi* Linsenmaier, 1959	1, 2					2	1, 2
*Chrysis subcoriacea* Linsenmaier, 1959	1	1, 2	1, 2	1		1, 2	1, 2
*Chrysis angustula* Schenck, 1856	1, 2	1, 2	1, 2	1, 2	1, 2	1, 2	1, 2
*Chrysis longula* Abeille de Perrin, 1879	1, 2	1, 2	1, 2	1, 2	1, 2	1, 2	1, 2
*Chrysis brevitarsis* Thomson, 1870		1, 2	1, 2		1		1, 2
*Chrysis pseudobrevitarsis* Linsenmaier, 1951	1	1, 2	1, 2	1	1, 2	2	1, 2
*Chrysis mediata* Linsenmaier, 1951	2	1, 2		1	1, 2		1, 2
*Chrysis solida* Haupt, 1957	1	1, 2	1, 2	1	1, 2	1, 2	1, 2
*Chrysis leptomandibularis* Niehuis, 2000		1, 2	1	1	1, 2	2	
*Chrysis schencki* Linsenmaier, 1968	1, 2	1, 2	1, 2	1	1, 2	1, 2	1, 2
*Chrysis ignita* (Linnaeus, 1758)	1, 2	1, 2	1, 2	1, 2	1, 2	1, 2	1, 2
*Chrysis impressa* Schenck, 1856	1	1, 2	1, 2	1	1, 2	1, 2	1, 2
*Chrysis borealis* Paukkunen, Ødegaard & Soon, sp. n.	1	1, 2	1, 2			1, 2	1, 2
*Chrysis terminata* Dahlbom, 1854				1	1, 2	1, 2	1, 2
*Chrysis sexdentata* Christ, 1791				1			
*Chrysis equestris* Dahlbom, 1854		1, 2	1, 2		1, 2	1, 2	1, 2
*Chrysis zetterstedti* Dahlbom, 1845		1, 2		1	1, 2		1, 2
*Trichrysis cyanea* (Linnaeus, 1758)	1, 2	1, 2	1, 2	1, 2	1, 2	1, 2	1, 2
*Chrysura austriaca* (Fabricius, 1804)					1, 2		
*Chrysura dichroa* (Dahlbom, 1854)							1
*Chrysura hirsuta* (Gerstaecker, 1869)		1, 2	1, 2			1, 2	1, 2
*Chrysura radians* (Harris, 1776)	1	1, 2		1	1, 2	1, 2	1, 2
*Chrysura trimaculata* (Förster, 1853)							1, 2
*Parnopes grandior* (Pallas, 1771)					1, 2		
Number of species	47	51	48	49	48	38	53

## Supplementary Material

XML Treatment for Cleptes

XML Treatment for Cleptes nitidulus

XML Treatment for Cleptes semicyaneus

XML Treatment for Cleptes semiauratus

XML Treatment for Omalus

XML Treatment for Omalus biaccinctus

XML Treatment for Omalus aeneus

XML Treatment for Omalus puncticollis

XML Treatment for Pseudomalus

XML Treatment for Pseudomalus pusillus

XML Treatment for Pseudomalus auratus

XML Treatment for Pseudomalus triangulifer

XML Treatment for Pseudomalus violaceus

XML Treatment for Philoctetes

XML Treatment for Philoctetes truncatus

XML Treatment for Elampus

XML Treatment for Elampus constrictus

XML Treatment for Elampus foveatus

XML Treatment for Elampus panzeri

XML Treatment for Holopyga

XML Treatment for Holopyga fervida

XML Treatment for Holopyga metallica

XML Treatment for Holopyga generosa

XML Treatment for Holopyga inflammata

XML Treatment for Hedychrum

XML Treatment for Hedychrum gerstaeckeri

XML Treatment for Hedychrum rutilans

XML Treatment for Hedychrum nobile

XML Treatment for Hedychrum niemelai

XML Treatment for Hedychrum chalybaeum

XML Treatment for Hedychridium

XML Treatment for Hedychridium zelleri

XML Treatment for Hedychridium ardens

XML Treatment for Hedychridium coriaceum

XML Treatment for Hedychridium cupreum

XML Treatment for Hedychridium purpurascens

XML Treatment for Hedychridium caputaureum

XML Treatment for Hedychridium roseum

XML Treatment for Pseudospinolia

XML Treatment for Pseudospinolia neglecta

XML Treatment for Spinolia

XML Treatment for Spinolia unicolor

XML Treatment for Chrysis

XML Treatment for Chrysis gracillima

XML Treatment for Chrysis bicolor

XML Treatment for Chrysis westerlundi

XML Treatment for Chrysis illigeri

XML Treatment for Chrysis succincta

XML Treatment for Chrysis leachii

XML Treatment for Chrysis scutellaris

XML Treatment for Chrysis splendidula

XML Treatment for Chrysis rutilans

XML Treatment for Chrysis pulcherrima

XML Treatment for Chrysis viridula

XML Treatment for Chrysis graelsii

XML Treatment for Chrysis indigotea

XML Treatment for Chrysis fulgida

XML Treatment for Chrysis iris

XML Treatment for Chrysis ruddii

XML Treatment for Chrysis corusca

XML Treatment for Chrysis clarinicollis

XML Treatment for Chrysis vanlithi

XML Treatment for Chrysis subcoriacea

XML Treatment for Chrysis angustula

XML Treatment for Chrysis longula

XML Treatment for Chrysis brevitarsis

XML Treatment for Chrysis pseudobrevitarsis

XML Treatment for Chrysis mediata

XML Treatment for Chrysis solida

XML Treatment for Chrysis leptomandibularis

XML Treatment for Chrysis schencki

XML Treatment for Chrysis ignita

XML Treatment for Chrysis impressa

XML Treatment for Chrysis borealis

XML Treatment for Chrysis terminata

XML Treatment for Chrysis sexdentata

XML Treatment for Chrysis equestris

XML Treatment for Chrysis zetterstedti

XML Treatment for Trichrysis

XML Treatment for Trichrysis cyanea

XML Treatment for Chrysura

XML Treatment for Chrysura austriaca

XML Treatment for Chrysura dichroa

XML Treatment for Chrysura hirsuta

XML Treatment for Chrysura radians

XML Treatment for Chrysura trimaculata

XML Treatment for Parnopes

XML Treatment for Parnopes grandior
